# Putative causal inference for the relationship between obesity and sex hormones in males: a bidirectional Mendelian randomization study

**DOI:** 10.7717/peerj.15760

**Published:** 2023-07-19

**Authors:** Bangbei Wan, Ning Ma, Zhi Zhou, Cai Lv

**Affiliations:** 1 Department of Urology, Affiliated Haikou Hospital of Xiangya Medical College, Central South University, Haikou, Hainan, China; 2 Reproductive Medical Center, Hainan Women and Children’s Medical Center, Haikou, Hainan, China

**Keywords:** Sex hormone-binding globulin, Bioavailable testosterone, Body mass index, Mendelian randomization, Single-nucleotide polymorphism

## Abstract

**Background:**

Obesity is a chronic disease with a high prevalence rate and is an established risk factor for human health. Body mass index (BMI) is a common and primary indicator used in assessing obesity. This work aims to investigate the putative causal relationship among BMI, sex hormone-binding globulin (SHBG), bioavailable testosterone (BioT), and estradiol levels.

**Materials and Methods:**

We conducted a bidirectional Mendelian randomization study, using single-nucleotide polymorphisms (SNPs) strongly associated with BMI, SHBG, BioT, and estradiol as instrumental variables. All SNPs were identified from the genome-wide association study (GWAS) summary data of large sample studies recruiting more than 150,000 European adult male individuals. The inverse-variance-weighted (IVW) approach was used as a primary algorithm for putative causal estimation.

**Results:**

Genetically predicted elevated BMI was associated with decreased SHBG (IVW, β = −0.103, 95% confidence interval [CI] [−0.113 to −0.092], *P* = 1.50 × 10^−77^) and BioT levels (IVW, β = −0.139, 95% CI [−0.165 to −0.113], *P* = 9.54 × 10^−26^) and high estradiol levels (IVW, β = 0.014, 95% CI [0.009–0.019], *P* = 2.19 × 10^−7^). Increased SHBG levels were causally associated with low BMI (IVW, β = −0.051, 95% CI [−0.098 to −0.005], *P* = 0.030) and BioT (IVW, β = −0.126, 95% CI [−0.175 to −0.077], *P* = 5.97 × 10^−7^) and high estradiol levels (IVW, β = 0.046, 95% CI [0.035–0.056], *P* = 6.51 × 10^−17^). Conversely, no evidence of an effect of estradiol imbalance on SHBG levels (IVW, β = 1.035, 95% CI [−0.854 to 2.926], *P* = 0.283) and BMI (IVW, β = 0.091, 95% CI [−0.094 to 0.276], *P* = 0.336) was obtained. However, increased BioT levels were causally associated with lower SHBG levels (IVW, β = −0.044, 95% CI [−0.061 to −0.026], *P* = 8.76 × 10^−7^), not BMI (IVW, β = −0.006, 95% CI [−0.035 to 0.023], *P* = 0.679).

**Conclusions:**

The findings support a network putative causal relationship among BMI, SHBG, BioT, and estradiol. SHBG, BioT, and estradiol may partly mediate the effect of obesity on male health. Reasonably modulating BioT and estradiol, especially SHBG, facilitated the attenuation of the harmful effects of obesity on male health.

## Introduction

Obesity is a highly prevalent chronic disease (Burki, 2021) and seriously endangers public health (Kivimaki et al., 2022; Salah, Ghandour & Husseini, 2021). The worldwide prevalence of obesity has increased markedly during the past three decades (Ng et al., 2014; Pan, Wang & Pan, 2021; Withrow & Alter, 2011). According to the Global Burden of Disease Study, approximately 603.7 million adults were obese in 2015, double the number in 1980 (GBD 2015 Obesity Collaborators, 2017). The prevailing rate of obesity in adults was 39.8% in the United States in 2015–2016 (Hales et al., 2017). A demographic investigation indicated that the morbidity rate of obesity was approximately 16.4% for adults in China in 2015–2019 (Pan, Wang & Pan, 2021). Recent data from the WHO reported that the incidence was approximately 60% for overweight or obese individuals in the European population (Boutari & Mantzoros, 2022). Obesity is an established risk factor for many diseases, including cardiovascular diseases, diabetes, and cancer (Haslam & James, 2005; Kivimaki et al., 2022; Lingvay et al., 2022; Riaz et al., 2018), and body mass index (BMI) is a primary indicator for assessing obesity clinically (Smith & Smith, 2016). Although the prevalence rate of obesity varies by country or region, the overall trend is increasing yearly. Based on the harm of obesity to health, it is necessary to explore the potential mechanism of obesity to health to formulate public prevention policies.

Testosterone and estradiol play a crucial role in human growth and health (Kloner et al., 2016; McEwen & Milner, 2017; Mielke & Miller, 2021) and influence the development of the human nervous system by regulating many cellular and molecular processes (McEwen & Milner, 2017). They can affect human reproductive function by regulating spermatogenesis in males and follicular development in females (de Kretser et al., 1998; Roy, 1994). Imbalance in testosterone or estradiol level is associated with the risk of cardiovascular diseases (Kloner et al., 2016; Raparelli et al., 2022), diabetes (Saikia, Jabbar & Das, 2021; Vikan et al., 2010), and cancer (Ruth et al., 2020; Tin Tin, Reeves & Key, 2021). Sex hormone-binding globulin (SHBG) is a crucial protein that can bind to testosterone and estradiol in the blood and regulate the transport, tissue delivery, bioactivity, and metabolism of the hormones (Anderson, 1974; Bourebaba et al., 2022; Breckwoldt, Zahradnik & Wieacker, 1989; Winters, 2020). Obesity has a significant influence on male sex hormones and SHBG levels. Substantial differences in SHBG, testosterone, and estradiol levels have been reported among individuals with different BMIs (Grasa et al., 2017; Tchernof & Despres, 2000; Wei et al., 2009). Furthermore, obesity influences the specific binding of sex hormones to SHBG (Grasa et al., 2017). The effect of obesity on the risk of diseases can be mediated by sex hormones (Haffner et al., 1993). Therefore, understanding the potential causality between obesity and sex hormone level is essential to controlling the harmful effects of obesity on health.

Mendelian randomization (MR) is a powerful epidemiological method, and it mainly uses single-nucleotide polymorphisms (SNPs) from genome-wide association study (GWAS) data as instrumental variables to estimate the putative causal relationship between an exposure of interest and outcome of interest (Emdin, Khera & Kathiresan, 2017; Smith & Ebrahim, 2003). In this work, we investigated the potential causality among obesity (measured as BMI), sex hormones (bioavailable testosterone (BioT) and estradiol levels), and SHBG by using the MR method. The inverse-variance-weighted (IVW) method was utilized as a primary causal estimate algorithm. Given that obesity and sex hormone imbalance are hazardous to human health, further expounding the potential causality between obesity and sex hormone imbalance may facilitate the mapping of public health policy for promoting health.

## Materials and Methods

### Study design and data sources

**Figure 1:**
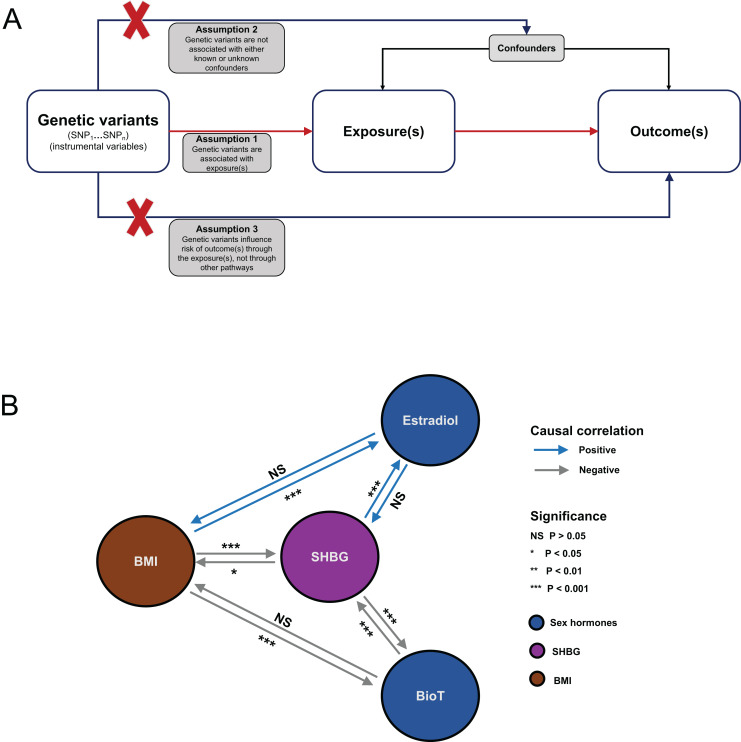
Flowchart of MR analysis and results summary. (A) MR causal schematic and three fundamental assumptions. (1) Relevance: genetic variants must be strongly associated with exposure of interest. (2) Independence: no unmeasured confounders of the associations between genetic variants and the outcome are found. (3) Exclusion restriction: genetic variants affect the outcome only through their effects on the exposure of interest. (B) Overview diagram of the MR investigation results. MR, Mendelian randomization; SNP, single-nucleotide polymorphism; BMI, body mass index; SHBG, sex hormone-binding globulin; BioT, bioavailable testosterone; NS, not significant.

The work is a bidirectional MR investigation. The study design is displayed in Fig. 1A. The GWAS summary-level data for SHBG, BioT, estradiol, and BMI were derived from the large sample studies of European ancestry. All GWAS data in this work are available in the IEU Open GWAS database (https://gwas.mrcieu.ac.uk/) and the Neale lab (http://www.nealelab.is/). BioT, estradiol, SHBG, and BMI were used as exposure data and outcomes in estimating the putative causal relationship between sex hormone-related traits and obesity. The IVW approach was used as the primary causal estimate algorithm in the bidirectional MR study.

### Data sets

GWAS summary-level data sample sizes for SHBG, BioT, and estradiol were 180,726; 178,782; and 206,927 European adult male participants, respectively (Ruth et al., 2020). The categories for SHBG and BioT levels were all continuous variables, whereas estradiol level was a binary variable. The unit for SHBG and BioT levels was standard deviation (SD). SHBG, BioT, and estradiol were all tested in serum. The GWAS summary-level data for BMI were obtained from the UK Biobank cohort and derived from a study with 166,413 European adult male individuals. In the study, BMI was a continuous variable, and its unit was 1-SD.

### Instrument selection

For each analysis, SNPs in the GWAS summary-level data were identified as instrumental variables according to a threshold of *P* < 5 × 10^−8^ for genome-wide significance. To ensure the independence of SNPs, we used the PLINK clumping method to eliminate the SNPs according to the parameters (*r*^2^ > 0.001 and clumping distance <10 Mb) in the 1,000 Genomes linkage disequilibrium reference panel (European population). To avoid potential pleiotropy, we utilized the PhenoScanner database (http://www.phenoscanner.medschl.cam.ac.uk/) to identify and eliminate SNPs that were associated with confounding factors or outcomes, with a threshold of *P* < 5 × 10^−8^ for genome-wide significance. The F statistic was used in assessing instrument strength. Its calculation method was reported in a previous study (Pierce, Ahsan & Vanderweele, 2011). An F statistic of >10 was regarded as the threshold that denotes the absence of weak instrument bias.

### Statistical analyses

All MR analyses were conducted using the packages TwoSampleMR (version 0.5.6), MRPRESSO (version 1.0), and mr.raps (version 0.4.1) in the statistical software R (version 4.1.2; R Core Team, 2021).

### Bidirectional univariable MR analyses

Sex hormones (BioT and estradiol), SHBG, and BMI were used as exposure data and outcomes in clarifying the putative causality between sex hormone levels and obesity. The multiplicative random effects IVW was utilized as a primary analysis method for calculating the combined effects of exposure-related SNPs on the outcomes. When no heterogeneity was found among the SNPs, the fixed effects IVW was utilized in calculating the combined effects.

### Sensitivity analyses

The reliance and robustness of putative causal estimation in univariable MR were examined *via* a series of sensitivity analysis methods. First, four other methods: MR–Egger (Bowden, Davey Smith & Burgess, 2015), maximum likelihood (Xue, Shen & Pan, 2021), MR–pleiotropy residual sum outlier (MR–PRESSO) (Verbanck et al., 2018), and robust adjusted profile score (MR–RAPS) (Zhao et al., 2020) methods, were used in examining the reliability of putative causal estimation from the IVW method. Second, heterogeneity among the SNPs was evaluated using Cochran’s Q value in the IVW and MR–Egger models. A *P* < 0.05 was regarded as the cutoff value of existing significant heterogeneity. Third, MR–Egger regression was used in assessing potential horizontal pleiotropy. The intercept of MR–Egger regression approximates 0, and a *P* > 0.05 indicated no pleiotropy for SNPs. Fourth, three methods, namely, MR–PRESSO, MR‒Egger, and IVW methods, were used in identifying and removing potential outliers that were invalid instruments and led to underlying pleiotropy. Fifth, leave-one-out iteration analysis was used in detecting whether a single SNP significantly altered the causal combined effects of IVW method estimation. Finally, the correctness of the putative causal direction from exposure to outcome was examined using the MR–Steiger test (Hemani, Tilling & Davey Smith, 2017). A *P* < 0.05 indicated that the putative causal assumption of the effect of exposure on outcome was correct.

## Results

A summary of the analysis results is shown in Fig. 1B. For each MR analysis, the F statistics of the instrumental variables were all greater than 10. Detailed information on SNPs used as instrumental variables can be found in Tables S1–S10 in the Supplemental Materials.

### Effect of BMI on BioT, estradiol, and SHBG

**Figure 2:**
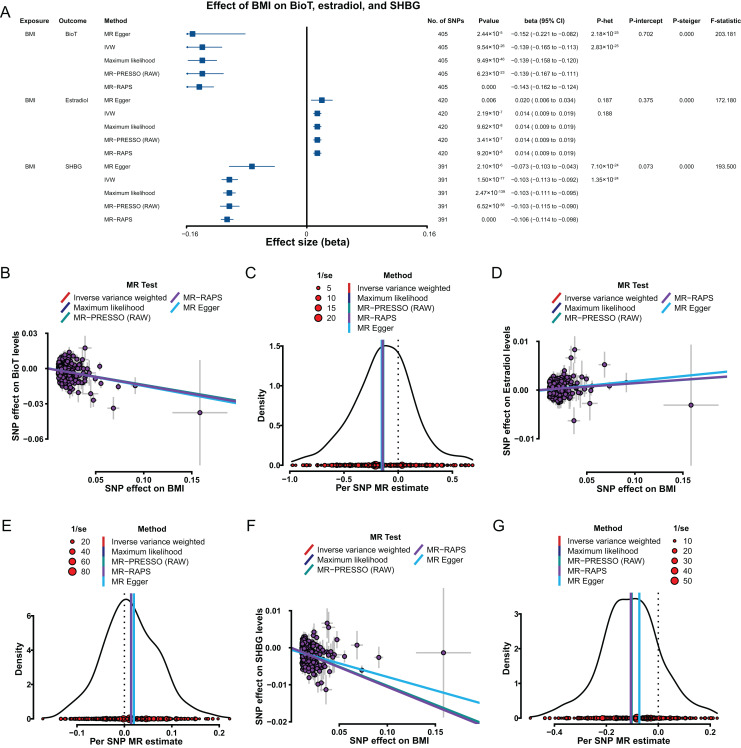
MR results of the effect of BMI on BioT, estradiol, and SHBG levels. (A) Forest plot shows MR results of the effect of BMI on BioT, estradiol, and SHBG levels; (B) scatter plots indicate the causal effect of BMI on BioT levels using five methods; (C) density plots denote the effect of BMI-related SNPs on BioT levels with five methods; (D) scatter plots indicate the causal effect of BMI on estradiol levels using five methods; (E) density plots denote the effect of BMI-related SNPs on estradiol levels with five methods; (F) scatter plots indicate the causal effect of BMI on SHBG levels using five methods; (G) density plots denote the effect of BMI-related SNPs on SHBG levels with five methods. MR, Mendelian randomization; SNP, single-nucleotide polymorphism; BMI, body mass index; SHBG, sex hormone-binding globulin; BioT, bioavailable testosterone; IVW, inverse-variance-weighted; MR–PRESSO, MR–pleiotropy residual sum outlier; MR–RAPS, robust adjusted profile score; CI, confidence interval; *P*-het, *P* value for heterogeneity based on Cochran Q test; *P*-intercept, *P* value for MR‒Egger intercept; *P*-Steiger, *P* value for MR–Steiger test.

Genetically predicted, increase in BMI was associated with a reduction in BioT (IVW, β = −0.139, 95% confidence interval [CI] [−0.165 to −0.113], *P* = 9.54 × 10^−26^) and SHBG (IVW, β = −0.103, 95% CI [−0.113 to −0.092], *P* = 1.50 × 10^−77^) levels (Fig. 2A). A genetically predicted increase in BMI was associated with high estradiol levels with a β of 0.014 (IVW, 95% CI [0.009–0.019], *P* = 2.19 × 10^−7^; Fig. 2A).

The robustness and reliability of the above putative causal estimations were tested and verified. The results of four methods (MR–Egger, maximum likelihood, MR–PRESSO, and MR–RAPS) consistently supported the putative causal direction of BMI influence on BioT, estradiol, and SHBG levels (Figs. 2B–2G). The results of heterogeneity tests showed existing heterogeneity for the effect of the BMI-related SNPs on BioT and SHBG (all *P* < 0.05), except estradiol (all *P* > 0.05; Fig. 2A). The results from pleiotropy tests indicated no pleiotropy for BMI-related SNPs above each analysis (all *P* > 0.05; Fig. 2A). The leave-one-out analyses showed that no single SNP could reverse for above each IVW putative causal estimation (all *P* < 0.05; Tables S11–S13 in Supplemental Materials). The results of the MR–Steiger test supported that the effects of BMI on BioT, estradiol, and SHBG levels were all correct putative causal directions (all *P* < 0.05; Fig. 2A).

### Effects of BioT, estradiol, and SHBG on BMI

**Figure 3:**
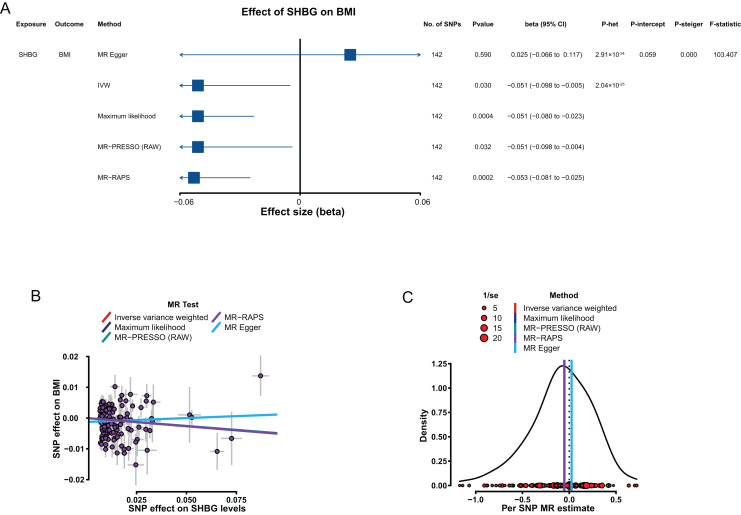
MR results of the effect of SHBG levels on BMI. (A) Forest plot shows MR results of the effect of SHBG levels on BMI; (B) scatter plots indicate the causal effect of SHBG levels on BMI using five methods; (C) density plots denote the effect of SHBG-related SNPs on BMI with five methods. MR, Mendelian randomization; SNP, single-nucleotide polymorphism; BMI, body mass index; SHBG, sex hormone-binding globulin; IVW, inverse-variance-weighted; MR–PRESSO, MR–pleiotropy residual sum outlier; MR–RAPS, robust adjusted profile score; CI, confidence interval; *P*-het, *P* value for heterogeneity based on Cochran Q test; *P*-intercept, *P* value for MR‒Egger intercept; *P*-Steiger, *P* value for MR–Steiger test.

The results from the IVW method showed that genetically predicted increased SHBG levels were associated with low BMI with a β of −0.051 (95% CI [−0.098 to −0.005], *P* = 0.030; Fig. 3A). However, the putative causality of the effect of BioT (IVW, β = −0.006, 95% CI [−0.035 to 0.023], *P* = 0.679) and estradiol (IVW, β = 0.091, 95% CI [−0.094 to 0.276], *P* = 0.336) on BMI was not statistically significant.

In sensitivity analyses, the four methods (MR–Egger, maximum likelihood, MR–PRESSO, and MR–RAPS) were consistent with IVW for estimating the effect of SHBG on BMI (Figs. 3B and 3C). The heterogeneity analysis results showed certain heterogeneity in the effects of the SHBG-related SNPs on BMI (*P* < 0.05; Fig. 3A). The results from the pleiotropy test indicated no pleiotropy for the putative causal estimation of the effect of the SHBG-related SNPs on BMI (*P* > 0.05; Fig. 3A). The leave-one-out analyses showed that no single SNP significantly altered the putative causal estimation of the effect of the SHBG-related SNPs on BMI (*P* < 0.05; Tables S14 in Supplemental Materials). The result of the MR–Steiger test indicated that the effect of SHBG on BMI was a correct putative causal assumption (*P* < 0.05; Fig. 3A).

### Effect of SHBG on BioT and estradiol

Genetically predicted elevated SHBG levels were associated with decrease in BioT level (IVW, β = −0.126, 95% CI [−0.175 to −0.077], *P* = 5.97 × 10^−7^) and high estradiol level (IVW, β = 0.046, 95% CI [0.035–0.056], *P* = 6.51 × 10^−17^).

**Figure 4:**
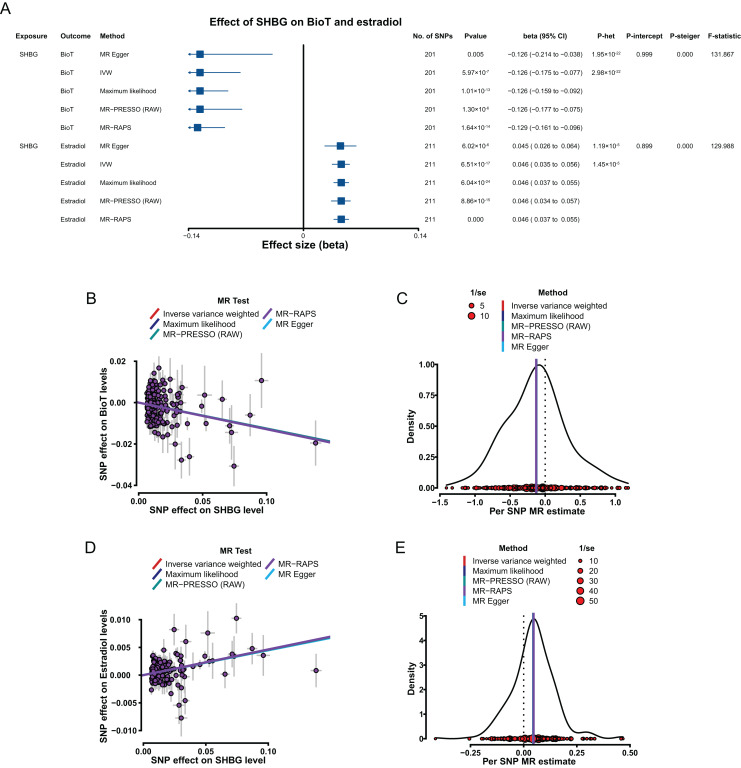
MR results of the effect of SHBG levels on BioT and estradiol levels. (A) Forest plot shows MR results of the effect of SHBG levels on BioT and estradiol levels; (B) scatter plots indicate the causal effect of SHBG levels on BioT levels using five methods; (C) density plots denote the effect of SHBG-related SNPs on BioT levels with five methods; (D) scatter plots indicate the causal effect of SHBG levels on estradiol levels using five methods; (E) density plots denote the effect of SHBG-related SNPs on estradiol levels with five methods. MR, Mendelian randomization; SNP, single-nucleotide polymorphism; SHBG, sex hormone-binding globulin; BioT, bioavailable testosterone; IVW, inverse-variance-weighted; MR–PRESSO, MR–pleiotropy residual sum outlier; MR–RAPS, robust adjusted profile score; CI, confidence interval; *P*-het, *P* value for heterogeneity based on Cochran Q test; *P*-intercept, *P* value for MR‒Egger intercept; *P*-Steiger, *P* value for MR–Steiger test.

In sensitivity analyses, the results, including the putative causal estimations of the four methods (MR–Egger, maximum likelihood, MR–PRESSO, and MR–RAPS; Figs. 4B–4E), heterogeneity analyses (Fig. 4A), pleiotropy tests (all *P* > 0.05; Fig. 4A), leave-one-out analyses (all *P* < 0.05; Tables S15–S16 in Supplemental Materials), and MR Steiger tests (all *P* < 0.05; Fig. 4A), all supported the putative causal assumption of the effect of SHBG on BioT and estradiol.

### Effects of BioT and estradiol on SHBG

**Figure 5:**
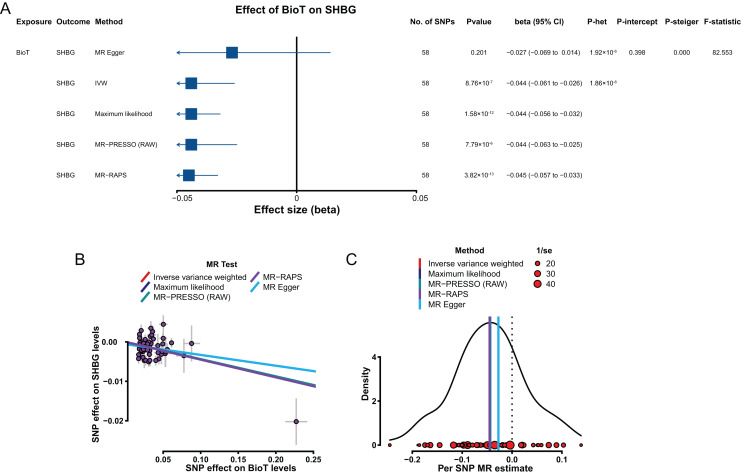
MR results of the effect of BioT levels on SHBG levels. (A) Forest plot shows MR results of the effect of BioT levels on SHBG levels; (B) scatter plots indicate the causal effect of BioT levels on SHBG levels using five methods; (C) density plots denote the effect of BioT-related SNPs on SHBG levels with five methods. MR, Mendelian randomization; SNP, single-nucleotide polymorphism; SHBG, sex hormone-binding globulin; BioT, bioavailable testosterone; IVW, inverse-variance-weighted; MR–PRESSO, MR–pleiotropy residual sum outlier; MR–RAPS, robust adjusted profile score; CI, confidence interval; *P*-het, *P* value for heterogeneity based on Cochran Q test; *P*-intercept, *P* value for MR‒Egger intercept; *P*-Steiger, *P* value for MR–Steiger test.

Genetically predicted elevated BioT levels were correlated with low SHBG levels with a β of −0.044 (IVW, 95% CI [−0.061 to −0.026], *P* = 8.76 × 10^−7^; Fig. 5A). However, the genetically predicted effect of estradiol levels on SHBG levels (IVW, β = 1.035, 95% CI [−0.854 to 2.926], *P* = 0.283) was not significantly putative causal.

In sensitivity analyses, the results, including the putative causal estimations of four methods (MR–Egger, maximum likelihood, MR–PRESSO, and MR–RAPS; Figs. 5B and 5C), heterogeneity analyses (Fig. 5A), pleiotropy tests (*P* > 0.05; Fig. 5A), leave-one-out analysis (*P* < 0.05; Table S17), and MR Steiger tests (*P* < 0.05; Fig. 5A), all supported the putative causal hypothesis of the effect of BioT on SHBG.

## Discussion

We comprehensively assessed the putative causal relationship among BMI, BioT, estradiol, and SHBG by using large-sample GWAS data. A summary of the findings is shown in Fig. 1B. We found that genetically predicted BMI was bidirectionally associated with SHBG levels and unidirectionally associated with BioT and estradiol levels. In addition, genetically predicted SHBG levels were bidirectionally correlated with BioT levels and unidirectionally correlated with estradiol levels.

Obesity, imbalance in sex hormones, and SHBG are all closely related to the risk of human diseases (Arnold et al., 2017; Boese et al., 2017; Guarner-Lans et al., 2011). The effects of obesity on human health may be partly mediated by sex hormones and SHBG levels (Grasa et al., 2017; Guarner-Lans et al., 2011; MacDonald et al., 2010). However, the correlation among BMI, sex hormones, and SHBG remains controversial. For example, some observational studies have reported that BMI is not associated with serum free testosterone and estradiol levels (Hofny et al., 2010; Winters et al., 2006). However, many observational data have indicated that BMI is negatively associated with serum free testosterone and SHBG levels (Foresta et al., 2009; Macdonald, Stewart & Farquhar, 2013; Yamacake et al., 2016) and positively associated with estradiol levels in males (Bieniek et al., 2016; Foresta et al., 2009; Jensen et al., 2004). Similarly, a recent meta-analysis including data from 28 articles revealed that increased BMI is associated with low serum testosterone and SHBG levels and high estradiol levels in males (Salas-Huetos et al., 2021). Additionally, MR investigation findings from Loh et al. (2022) showed that elevated BMI is causally associated with reduced serum BioT levels (β = −0.128) and high serum estradiol levels (β = 0.012). However, the putative causal effect of BioT and estradiol level imbalances on BMI was not statistically significant (Loh et al., 2022). Our results supported that the effect of BMI on serum BioT (β = −0.139) and estradiol levels (β = 0.014) is a unidirectional causality. Although the findings of Loh et al. (2022) were valuable, our study has more advantages. Importantly, our MR study used multiple methods to remove the pleiotropic and outlying SNPs that cause potential bias of causal estimation, thereby improving the accuracy of putative causal estimation. Moreover, our findings obtained more powerful support from statistical evidence of sensitivity analyses, including the putative causal estimations of the four methods (MR‒Egger, maximum likelihood, MR–PRESSO, and MR–RAPS), pleiotropy tests, leave-one-out analyses, and MR Steiger tests. Mechanistically, as previous research reported, obesity can increase the conversion of testosterone to estradiol in males, thereby causing reproductive axis suppression and reduced secretion of endogenous gonadotropin (Michalakis et al., 2013). Similarly, a recent study has shown that obesity reduces testosterone and elevated estradiol levels by mediating the hypothalamic–pituitary–gonadal axis in males (Leisegang et al., 2021). Additionally, in the present work, we analyzed the putative causal association between BMI and SHBG and found that they were a bidirectional putative causal relationship. Previous observational data revealed that individuals with a high BMI have lower SHBG levels than those with a low BMI (Allan & McLachlan, 2010). In addition, a recent meta-analysis revealed that BMI is negatively correlated with SHBG levels in males (Salas-Huetos et al., 2021). Our findings showed that genetically predicted elevated BMI is inversely associated with serum SHBG levels in males. We also observed that genetically determined increased SHBG levels are causally associated with low BMI. Similarly, observational studies have shown that high SHBG levels are associated with lowered risk of metabolic syndrome (Lewis et al., 2004; Salas-Huetos et al., 2021). Mechanistic findings from animal model studies have suggested that SHBG overexpression can reduce the risk of obesity by increasing insulin, leptin, and resistin levels in male rats (Chen et al., 2010; Saez-Lopez et al., 2020). According to the above evidence, the influence of genetically predicted high BMI on males’ health may be partly mediated by SHBG and sex hormones. Consequently, the healthy modulation of SHBG and sex hormones can attenuate the harmful effects of obesity on male health.

Given the importance of SHBG to sex hormone transport, tissue delivery, and bioactivity and metabolism regulation, we further examined the putative causal association between SHBG and the two sex hormones (BioT and estradiol). Our results suggested a bidirectional causal association between SHBG and BioT. However, a unidirectional causal association was found between SHBG and estradiol. Serum SHBG levels are associated with serum testosterone and estradiol concentrations in males (Wan et al., 2021; Yeap et al., 2021). High serum SHBG levels can cause a decline in androgen/estrogen ratio (Wan et al., 2021). In this work, we found that genetically determined raised SHBG levels are associated with high estradiol and low BioT levels in males. Additionally, we observed that elevated BioT levels were associated with decreased SHBG levels in males. A similar phenomenon was observed in previous observational studies in which patients with prostate cancer had lower SHBG levels and higher free testosterone levels than healthy individuals (Travis et al., 2007; Watts et al., 2021, 2022). Although our work preliminarily revealed the causal association between SHBG and sex hormones, the potential mechanism of interaction among them has been completely clarified. Therefore, we should cautiously comprehend the findings, and the underlying mechanism of SHBG and sex hormones interacting and affecting one another should be explored using molecular experiments. Given the putative causality of SHBG linked with BMI and sex hormones, SHBG could be a promising target for eliminating adverse effects caused by obesity and sex hormone imbalance on physical health.

Our work has some merits. First, all GWAS summary-level data were derived from recent extensive sample studies, ensuring our study’s statistical strength. Second, we used multiple methods to eliminate outlying and pleiotropic SNPs to guarantee instrumental variables’ validity. Third, multiple sensitivity analyses consistently supported our findings, suggesting the robustness and reliability of the results. Finally, our work solves the limitation of conventional observational research that does not explain the putative causality among obesity, SHBG, and sex hormones or handle unmeasured confounders well (Davies, Holmes & Davey Smith, 2018).

Inevitably, our work has some limitations. First, although our findings supported a putative causal association among BMI, sex hormones, and SHBG, the potential interaction mechanism remains unclear. Consequently, the underlying mechanism of their interactions and how they affect one another should be further investigated using molecular experiments. Next, whether and how much samples of the sex hormones-related GWAS overlap is unclear. Furthermore, given that all GWAS summary data were derived from European populations, generalizing to non-European populations may have some bias. Finally, all data were GWAS summary levels, and thus stratified analysis according to age was limited.

## Conclusion

In summary, using genetic data from large sample studies, we conducted an MR study to expound the bidirectional causal association among BMI, sex hormones, and SHBG in men. We then found a bidirectional causality between BMI and SHBG and a unidirectional causality between BMI and sex hormones. Additionally, genetically predicted SHBG levels were bidirectionally associated with BioT levels and unidirectionally associated with estradiol levels. The findings provide novel insights into interventions based on BMI, sex hormones, and SHBG, specifically interventions that promote men’s health.

## Supplemental Information

10.7717/peerj.15760/supp-1 Supplemental Information 1 Supplementary Tables. Details of instrumental variables for the MR study.

10.7717/peerj.15760/supp-2 Supplemental Information 2 Supplementary Tables. Results of leave-one-out analyses for the MR study.

10.7717/peerj.15760/supp-3 Supplemental Information 3 The STROBE-MR checklist, RAW data and R Code.

## Additional Information and Declarations

### Data Availability

The following information was supplied regarding data availability: The STROBE-MR checklist, RAW data and R Code are available in Supplemental Files.
