# Supplementary material for: Putative causal inference for the relationship between obesity and sex hormones in males: a bidirectional Mendelian randomization study
Source: PeerJ. 2023 Jul 19;11:e15760. doi: 10.7717/peerj.15760 (PMC10362853; doi:10.7717/peerj.15760)
Supplement: Supplemental Information 1 [file peerj-11-15760-s001.pdf]

Supplementary Table S1. Instruments for causal estimation from BMI to BioT level.

Exposure	Outcome	SNP	Effect allele	Other allele	Chromosome	Genetic position	Effect allele frequency	Beta	Standard error of beta	Pvalue	Sample size
Body mass index (BMI)	Bioavailable testosterone levels	rs10063055	T	C	5	140990108	0.253365	0.0136775	0.00226991	1.7e-09	461460
Body mass index (BMI)	Bioavailable testosterone levels	rs10099330	G	A	8	143383694	0.45285	0.0124247	0.00198907	4.20001e-10	461460
Body mass index (BMI)	Bioavailable testosterone levels	rs10160769	C	G	11	76474827	0.21751	-0.0155862	0.00242199	1.2e-10	461460
Body mass index (BMI)	Bioavailable testosterone levels	rs10169594	C	T	2	41637688	0.363139	0.0121875	0.00205506	2.99999e-09	461460
Body mass index (BMI)	Bioavailable testosterone levels	rs10182416	G	A	2	104242992	0.512223	0.0130378	0.00197134	3.69999e-11	461460
Body mass index (BMI)	Bioavailable testosterone levels	rs10423928	A	T	19	46182304	0.194358	-0.0340136	0.00249869	3.40017e-42	461460
Body mass index (BMI)	Bioavailable testosterone levels	rs10505836	C	A	12	19288508	0.860012	0.0184851	0.00287086	1.2e-10	461460
Body mass index (BMI)	Bioavailable testosterone levels	rs10510025	T	C	10	118650996	0.247013	0.0175643	0.00229886	2.19989e-14	461460
Body mass index (BMI)	Bioavailable testosterone levels	rs1064213	A	G	2	198950240	0.478377	0.0149301	0.00197196	3.69999e-14	461460
Body mass	Bioavailable	rs107427	C	T	11	45438374	0.612239	0.01179	0.00202961	6.1e-09	461460

index (BMI)	testosterone levels	52						79			
Body mass index (BMI)	Bioavailable testosterone levels	rs10756714	G	A	9	15885041	0.444196	-0.0207862	0.00199408	1.9002e-25	461460
Body mass index (BMI)	Bioavailable testosterone levels	rs10756792	T	C	9	16726119	0.742901	-0.0190582	0.00227201	4.90004e-17	461460
Body mass index (BMI)	Bioavailable testosterone levels	rs10760277	T	C	9	126093999	0.385049	0.0138734	0.00203825	1e-11	461460
Body mass index (BMI)	Bioavailable testosterone levels	rs10780248	A	G	9	81370555	0.559454	-0.0121232	0.00199415	1.2e-09	461460
Body mass index (BMI)	Bioavailable testosterone levels	rs10781401	T	C	8	142619393	0.383867	0.0142239	0.00205862	4.90004e-12	461460
Body mass index (BMI)	Bioavailable testosterone levels	rs10799778	G	T	1	23313353	0.833718	-0.0182237	0.00264876	6.00067e-12	461460
Body mass index (BMI)	Bioavailable testosterone levels	rs10809621	G	C	9	11859607	0.350143	-0.0125305	0.00207043	1.40001e-09	461460
Body mass index (BMI)	Bioavailable testosterone levels	rs10824211	T	C	10	76363107	0.139491	0.0207688	0.00286631	4.30031e-13	461460
Body mass index (BMI)	Bioavailable testosterone levels	rs10832778	G	C	11	17394073	0.623038	0.0115628	0.00203985	1.40001e-08	461460
Body mass index (BMI)	Bioavailable testosterone levels	rs10927006	C	T	1	243557659	0.143704	-0.0168156	0.00281467	2.30001e-09	461460
Body mass	Bioavailable	rs109656	T	C	9	23203619	0.369897	-0.0112	0.002052	3.89996e	461460

index	testosterone	98						741		-08	
(BMI)	levels										
Body	Bioavailabl										
mass	e	rs10						0.01		1.39	
index	testosterone	9890				10311		695	0.002123	991e	461
(BMI)	levels	67	A	G	9	9634	0.31593	14	49	-15	460
Body	Bioavailabl										
mass	e	rs11						0.01		7.29	
index	testosterone	0019				78760	0.58133	167	0.002019	995e	461
(BMI)	levels	63	T	C	10	959	8	94	21	-09	460
Body	Bioavailabl										
mass	e	rs11						-0.0		1.40	
index	testosterone	0096				34511	0.24409	130	0.002307	001e	461
(BMI)	levels	85	T	C	10	990	6	834	66	-08	460
Body	Bioavailabl										
mass	e	rs11						0.02		7.10	
index	testosterone	0127				21830	0.33168	164	0.002101	068e	461
(BMI)	levels	32	G	A	10	104	3	25	26	-25	460
Body	Bioavailabl										
mass	e	rs11						-0.0		1.80	
index	testosterone	0798				47090	0.32853	200	0.002111	011e	461
(BMI)	levels	49	T	C	17	785	1	93	63	-21	460
Body	Bioavailabl										
mass	e	rs11						-0.0		5.60	
index	testosterone	0990				13072	0.64060	142	0.002062	015e	461
(BMI)	levels	20	T	C	4	4902	9	038	02	-12	460
Body	Bioavailabl										
mass	e	rs11						-0.0		2.19	
index	testosterone	1151				82424	0.23779	130	0.002336	999e	461
(BMI)	levels	60	A	G	12	100	7	768	41	-08	460
Body	Bioavailabl										
mass	e	rs11						-0.0		9.09	
index	testosterone	1224				23030	0.61173	116	0.002024	997e	461
(BMI)	levels	50	G	T	1	1811	9	316	25	-09	460
Body	Bioavailabl										
mass	e	rs11						0.01		1.20	
index	testosterone	1346				17062	0.68475	824	0.002132	005e	461
(BMI)	levels	79	G	A	5	3391	3	59	86	-17	460
Body	Bioavailabl										
mass	e	rs11						-0.0		2.90	
index	testosterone	1507				78757	0.31771	211	0.002129	001e	461
(BMI)	levels	45	G	A	17	626	1	611	61	-23	460
Body	Bioavailabl	rs11				17163	0.20867	-0.0	0.002437	5.39	461
mass	e	1598	T	C	4	5471	1	142	98	995e	460

index	testosterone	585						245		-09	
(BMI)	levels										
Body	Bioavailabl										
mass	e	rs11						0.01		4.90	
index	testosterone	1656				96924	0.59010	933	0.002003	004e	461
(BMI)	levels	43	T	C	1	097	3	19	37	-22	460
Body	Bioavailabl										
mass	e	rs11						-0.0		4.70	
index	testosterone	1689				27175	0.28261	136	0.002195	002e	461
(BMI)	levels	389	C	G	5	962	4	706	22	-10	460
Body	Bioavailabl										
mass	e	rs11						-0.0		8.40	
index	testosterone	2185				12192		144	0.002021	04e-	461
(BMI)	levels	10	A	G	11	2587	0.40047	616	21	13	460
Body	Bioavailabl										
mass	e	rs11						0.03			
index	testosterone	2693				49399	0.03527	226	0.005358	1.7e-	461
(BMI)	levels	0	C	G	12	132	1	95	92	09	460
Body	Bioavailabl										
mass	e	rs11						-0.0		5.80	
index	testosterone	3079				14735		155	0.002515	003e	461
(BMI)	levels	574	T	C	4	4089	0.19279	896	98	-10	460
Body	Bioavailabl										
mass	e	rs11						0.01		1.80	
index	testosterone	3603				39564		860	0.002426	011e	461
(BMI)	levels	865	T	C	1	930	0.21205	13	89	-14	460
Body	Bioavailabl										
mass	e	rs11						0.01			
index	testosterone	3624				88326		504	0.002368	2.1e-	461
(BMI)	levels	107	A	G	14	386	0.22586	61	76	10	460
Body	Bioavailabl										
mass	e	rs11						-0.0		6.59	
index	testosterone	5258				13881	0.09770	239	0.003336	933e	461
(BMI)	levels	73	C	T	7	7193	1	781	03	-13	460
Body	Bioavailabl										
mass	e	rs11						0.01		3.10	
index	testosterone	6074				11503	0.48657	571	0.001991	027e	461
(BMI)	levels	76	C	A	11	7061	4	2	79	-15	460
Body	Bioavailabl										
mass	e	rs11						0.01		3.09	
index	testosterone	6106				12167	0.14817	648	0.002783	999e	461
(BMI)	levels	21	A	T	12	1133	4	69	18	-09	460
Body	Bioavailabl	rs11				99240	0.25173	-0.0	0.002280	2.69	461
mass	e	6306	A	G	15	947	9	126	82	998e	460

index (BMI)	testosterone levels	47						74		-08	
Body mass index (BMI)	Bioavailable testosterone levels	rs116374395	A	G	5	50723410	0.035444	0.0318817	0.00536236	2.80001e-09	461460
Body mass index (BMI)	Bioavailable testosterone levels	rs11642090	C	T	16	81730582	0.373536	0.0114184	0.00205925	2.90001e-08	461460
Body mass index (BMI)	Bioavailable testosterone levels	rs11656076	A	G	17	31464270	0.224774	-0.0154285	0.00237054	7.59976e-11	461460
Body mass index (BMI)	Bioavailable testosterone levels	rs1167311	A	G	1	49996959	0.681309	-0.0192649	0.00213109	1.59993e-19	461460
Body mass index (BMI)	Bioavailable testosterone levels	rs11675464	G	A	2	204053742	0.562988	0.0119751	0.00198558	1.6e-09	461460
Body mass index (BMI)	Bioavailable testosterone levels	rs11691869	A	C	2	100805996	0.362038	-0.0193158	0.00205562	5.60015e-21	461460
Body mass index (BMI)	Bioavailable testosterone levels	rs11699828	A	G	20	62157198	0.035832	-0.0335418	0.00582677	8.60003e-09	461460
Body mass index (BMI)	Bioavailable testosterone levels	rs11709402	G	A	3	131551027	0.278732	0.0228192	0.00220796	4.90004e-25	461460
Body mass index (BMI)	Bioavailable testosterone levels	rs117118217	C	G	10	131783328	0.017711	0.0448186	0.00788004	1.29999e-08	461460
Body mass index (BMI)	Bioavailable testosterone levels	rs117342986	T	C	16	54267868	0.026415	0.0365794	0.00646781	1.6e-08	461460
Body mass	Bioavailable	rs117572	C	T	6	13180454	0.303879	-0.0146	0.00214834	9.8992e-	461460

index	testosterone	78						269		12	
(BMI)	levels										
Body	Bioavailabl										
mass	e	rs11						0.01		4.20	
index	testosterone	7782				87762	0.16312	579	0.002687	001e	461
(BMI)	levels	19	G	A	8	607	3	6	4	-09	460
Body	Bioavailabl										
mass	e	rs11						-0.0			
index	testosterone	8136				21681	0.28108	132	0.002201	1.7e-	461
(BMI)	levels	827	T	G	17	04	6	582	9	09	460
Body	Bioavailabl										
mass	e	rs11						-0.0		1.40	
index	testosterone	9196				48085	0.67977	127	0.002112	001e	461
(BMI)	levels	65	T	A	3	349	5	854	92	-09	460
Body	Bioavailabl										
mass	e	rs12						0.01		3.29	
index	testosterone	0727				98315	0.22447	570	0.002366	989e	461
(BMI)	levels	39	G	A	1	893	8	08	91	-11	460
Body	Bioavailabl										
mass	e	rs12						0.01		8.90	
index	testosterone	0882				80798	0.30083	393	0.002148	02e-	461
(BMI)	levels	84	T	C	1	635	8	17	54	11	460
Body	Bioavailabl										
mass	e	rs12						-0.0		1.20	
index	testosterone	1401				62579	0.09425	330	0.003458	005e	461
(BMI)	levels	53	T	G	1	891	2	75	9	-21	460
Body	Bioavailabl										
mass	e	rs12						-0.0		2.99	
index	testosterone	1496				70309	0.11495	227	0.003115	985e	461
(BMI)	levels	60	A	G	16	237	6	338	86	-13	460
Body	Bioavailabl										
mass	e	rs12						0.01		4.49	
index	testosterone	2594				53680		308	0.001986	987e	461
(BMI)	levels	64	A	G	10	099	0.48447	95	86	-11	460
Body	Bioavailabl										
mass	e	rs12						0.02		6.80	
index	testosterone	2735				11691	0.05650	483	0.004285	002e	461
(BMI)	levels	45	T	C	11	1012	5	93	51	-09	460
Body	Bioavailabl										
mass	e	rs12						0.03		4.60	
index	testosterone	2998				10023	0.97277	735	0.005993	002e	461
(BMI)	levels	4	C	T	4	9319	5	7	85	-10	460
Body	Bioavailabl	rs12				13460	0.16455	0.01	0.002665	4.90	461
mass	e	3644	G	T	11	1012	6	927	8	004e	460

index	testosterone	70						05		-13	
(BMI)	levels										
Body	Bioavailabl										
mass	e	rs12						-0.0		2.49	
index	testosterone	4593				18459	0.26820	170	0.002232	977e	461
(BMI)	levels	68	G	A	19	377	3	141	35	-14	460
Body	Bioavailabl										
mass	e	rs12						0.01		2.60	
index	testosterone	4629				30272	0.32969	958	0.002120	016e	461
(BMI)	levels	75	A	G	19	202	2	22	71	-20	460
Body	Bioavailabl										
mass	e	rs12						-0.0		1.59	
index	testosterone	5414				95585		143	0.002127	993e	461
(BMI)	levels	08	C	T	8	807	0.31742	261	23	-11	460
Body	Bioavailabl										
mass	e	rs12						0.01			
index	testosterone	6687				51779	0.34961	409	0.002071	1e-1	461
(BMI)	levels	4	G	A	6	638	9	66	2	1	460
Body	Bioavailabl										
mass	e	rs12						0.01		3.50	
index	testosterone	6817				62054		486	0.002516	002e	461
(BMI)	levels	92	A	C	8	463	0.1926	93	93	-09	460
Body	Bioavailabl										
mass	e	rs12						0.01		1.29	
index	testosterone	6925				16126		308	0.002037	999e	461
(BMI)	levels	96	T	C	2	5910	0.37185	72	38	-10	460
Body	Bioavailabl										
mass	e	rs12						-0.0		3.50	
index	testosterone	6960				15630	0.14940	152	0.002770	002e	461
(BMI)	levels	39	G	A	3	4750	5	802	5	-08	460
Body	Bioavailabl										
mass	e	rs12						0.01		6.20	
index	testosterone	8605				91458	0.70374	491	0.002169	012e	461
(BMI)	levels	8	A	T	14	523	7	26	22	-12	460
Body	Bioavailabl										
mass	e	rs12						0.02		7.69	
index	testosterone	8816				10114		207	0.003589	999e	461
(BMI)	levels	29	G	A	14	6413	0.08265	48	29	-10	460
Body	Bioavailabl										
mass	e	rs12						0.02		3.79	
index	testosterone	9219				72312	0.07798	033	0.003698	997e	461
(BMI)	levels	86	G	A	16	727	4	27	3	-08	460
Body	Bioavailabl	rs12				34950	0.40816	-0.0	0.002013	1.39	461
mass	e	9374	T	C	17	239	7	171	83	991e	460

index	testosterone	11						87		-17	
(BMI)	levels										
Body	Bioavailabl										
mass	e	rs12						-0.0		3.90	
index	testosterone	9632				13708	0.55903	188		032e	461
(BMI)	levels	8	C	A	4	3193	2	62	0.001999	-21	460
Body	Bioavailabl										
mass	e	rs12						0.01		2.29	
index	testosterone	9744				18661	0.54317	524	0.001997	985e	461
(BMI)	levels	58	T	C	19	15	5	66	17	-14	460
Body	Bioavailabl										
mass	e	rs13						-0.0			
index	testosterone	0120				35447	0.22838	136	0.002347	6.1e-	461
(BMI)	levels	70	A	G	2	243	8	501	95	09	460
Body	Bioavailabl										
mass	e	rs13						0.01		3.29	
index	testosterone	0333				13352		261	0.002282	997e	461
(BMI)	levels	10	A	G	2	3605	0.25276	1	6	-08	460
Body	Bioavailabl										
mass	e	rs13						0.01			
index	testosterone	0979				35676	0.21231	455	0.002416	1.7e-	461
(BMI)	levels	18	A	T	3	330	5	61	1	09	460
Body	Bioavailabl										
mass	e	rs13						0.04		8.49	
index	testosterone	1073				10318		757	0.003754	963e	461
(BMI)	levels	25	T	C	4	8709	0.07492	99	79	-37	460
Body	Bioavailabl										
mass	e	rs13						0.01		3.10	
index	testosterone	1764				43152	0.68760	415	0.002131	027e	461
(BMI)	levels	29	C	T	5	216	9	55	22	-11	460
Body	Bioavailabl										
mass	e	rs13						-0.0		1.50	
index	testosterone	2025				21264	0.45479	180	0.001994	003e	461
(BMI)	levels	1	T	C	17	396	1	321	07	-19	460
Body	Bioavailabl										
mass	e	rs13						-0.0		5.79	
index	testosterone	2183				12017	0.33513	144	0.002092	963e	461
(BMI)	levels	83	G	C	6	3501	2	025	36	-12	460
Body	Bioavailabl										
mass	e	rs13						-0.0		1.09	
index	testosterone	2284				20488	0.60927	131	0.002035	999e	461
(BMI)	levels	2	G	A	6	897	4	292	39	-10	460
Body	Bioavailabl	rs13				14336	0.26855	0.01	0.002241	1.99	461
mass	e	2481	C	T	8	834	7	576	55	986e	460

index	testosterone	87						4		-12	
(BMI)	levels										
Body	Bioavailabl										
mass	e	rs13						-0.0		2.80	
index	testosterone	2725				51177	0.38772	148	0.002033	027e	461
(BMI)	levels	9	G	A	6	811	2	532	63	-13	460
Body	Bioavailabl										
mass	e	rs13						0.01		3.40	
index	testosterone	2917				80510	0.57068	103	0.001998	001e	461
(BMI)	levels	23	A	G	9	077	3	18	76	-08	460
Body	Bioavailabl										
mass	e	rs13						-0.0		3.29	
index	testosterone	3019				27760	0.48313	117	0.001986	997e	461
(BMI)	levels	9	T	G	9	946	7	509	42	-09	460
Body	Bioavailabl										
mass	e	rs13						-0.0		4.60	
index	testosterone	4200				50751	0.36503	154	0.002052	045e	461
(BMI)	levels	48	A	C	2	414	7	817	72	-14	460
Body	Bioavailabl										
mass	e	rs13						-0.0		5.60	
index	testosterone	4278				21341	0.27119	181	0.002241	015e	461
(BMI)	levels	22	G	A	2	4265	8	515	42	-16	460
Body	Bioavailabl										
mass	e	rs13						-0.0		9.70	
index	testosterone	4684				65651	0.40504	130	0.002017	063e	461
(BMI)	levels	1	A	G	4	730	1	56	2	-11	460
Body	Bioavailabl										
mass	e	rs13						0.01		4.79	
index	testosterone	6020				73796	0.48154	300	0.001977	954e	461
(BMI)	levels	1	T	C	9	450	6	75	47	-11	460
Body	Bioavailabl										
mass	e							-0.0		4.30	
index	testosterone	rs13				30432	0.36065	161	0.002056	031e	461
(BMI)	levels	642	T	A	11	220	4	363	95	-15	460
Body	Bioavailabl										
mass	e	rs14						-0.0		2.80	
index	testosterone	0159				73765	0.08229	246	0.003709	027e	461
(BMI)	levels	717	T	C	15	586	2	882	67	-11	460
Body	Bioavailabl										
mass	e	rs14						-0.0		1.09	
index	testosterone	3894				15251	0.71511	133	0.002197	999e	461
(BMI)	levels	5	A	T	5	0937	9	824	91	-09	460
Body	Bioavailabl	rs14				79580	0.59368	0.01	0.002058	3.40	461
mass	e	4126	A	G	13	919	1	790	54	017e	460

index	testosterone	4						33		-18	
(BMI)	levels										
Body	Bioavailabl										
mass	e	rs14						0.02		7.39	
index	testosterone	5196				41350	0.08226	220	0.003605	997e	461
(BMI)	levels	3	T	G	14	367	6	1	85	-10	460
Body	Bioavailabl										
mass	e	rs14						0.01		1.20	
index	testosterone	5815				41887	0.48843	407	0.001979	005e	461
(BMI)	levels	6	T	C	12	940	1	5	51	-12	460
Body	Bioavailabl										
mass	e	rs14						0.02			
index	testosterone	5981				74714	0.06371	271	0.004045	2e-0	461
(BMI)	levels	104	G	A	8	869	1	1	52	8	460
Body	Bioavailabl										
mass	e	rs14						0.01			
index	testosterone	6569				21996	0.20074	395	0.002485	2e-0	461
(BMI)	levels	428	A	G	11	86	9	33	71	8	460
Body	Bioavailabl										
mass	e	rs14						0.01		4.10	
index	testosterone	7109				10803	0.61666	346	0.002039	015e	461
(BMI)	levels	3	A	G	3	1094	3	19	96	-11	460
Body	Bioavailabl										
mass	e	rs14						0.01		8.90	
index	testosterone	7174				13632		935	0.002254	02e-	461
(BMI)	levels	0	C	T	3	8270	0.7405	76	04	18	460
Body	Bioavailabl										
mass	e	rs14						-0.0		1.09	
index	testosterone	7568				93061		133	0.002330	999e	461
(BMI)	levels	678	C	T	10	851	0.23807	327	34	-08	460
Body	Bioavailabl										
mass	e	rs14						0.03		2.19	
index	testosterone	7729				87988	0.13694	377	0.002898	989e	461
(BMI)	levels	0	C	T	5	934	7	72	15	-31	460
Body	Bioavailabl										
mass	e	rs14						-0.0		1.29	
index	testosterone	7730				12302	0.08724	350	0.003583	987e	461
(BMI)	levels	268	T	G	12	4476	3	799	57	-22	460
Body	Bioavailabl										
mass	e	rs15						0.01		5.90	
index	testosterone	0352				63020	0.48007	543	0.001976	065e	461
(BMI)	levels	6	C	T	5	706	7	08	81	-15	460
Body	Bioavailabl	rs15				10484	0.75345	0.01	0.002289	8.30	461
mass	e	6201	C	G	6	7441	5	318	32	004e	460

index (BMI)	testosterone levels							99		-09	
Body mass index (BMI)	Bioavailable testosterone levels	rs156914	A	G	1	16848652	0.491786	0.0111552	0.00197308	1.6e-08	461460
Body mass index (BMI)	Bioavailable testosterone levels	rs1582931	A	G	5	122657199	0.473242	-0.0133419	0.0019956	2.29985e-11	461460
Body mass index (BMI)	Bioavailable testosterone levels	rs1608113	T	A	3	157815217	0.364998	-0.0117766	0.00205086	9.29994e-09	461460
Body mass index (BMI)	Bioavailable testosterone levels	rs1609010	G	A	8	77227464	0.565728	0.0209773	0.0019963	7.89951e-26	461460
Body mass index (BMI)	Bioavailable testosterone levels	rs16916303	G	A	9	30823761	0.119736	-0.0192369	0.00307931	4.20001e-10	461460
Body mass index (BMI)	Bioavailable testosterone levels	rs17056301	C	T	5	158271680	0.256425	0.0135831	0.0022697	2.19999e-09	461460
Body mass index (BMI)	Bioavailable testosterone levels	rs17132130	C	G	7	2108036	0.221467	-0.0178418	0.00238564	7.50067e-14	461460
Body mass index (BMI)	Bioavailable testosterone levels	rs17149254	C	T	7	76634463	0.804854	-0.0213507	0.00255804	7.00003e-17	461460
Body mass index (BMI)	Bioavailable testosterone levels	rs17289010	G	A	4	140774684	0.32787	-0.0134661	0.00210438	1.6e-10	461460
Body mass index (BMI)	Bioavailable testosterone levels	rs17399739	G	A	10	87490850	0.068893	0.0270713	0.00391006	4.40048e-12	461460
Body mass	Bioavailable	rs174462	G	C	13	40762556	0.165544	0.01532	0.00266515	8.9e-09	461460

index	testosterone	99						35			
(BMI)	levels										
Body	Bioavailabl										
mass	e	rs17						0.01		5.30	
index	testosterone	5443				11529	0.21086	409	0.002414	005e	461
(BMI)	levels	84	C	T	1	5160	4	31	34	-09	460
Body	Bioavailabl										
mass	e	rs17						-0.0		1.50	
index	testosterone	6683				61208	0.14602	230	0.002793	003e	461
(BMI)	levels	56	G	C	3	619	9	543	18	-16	460
Body	Bioavailabl										
mass	e	rs17						0.02		1.29	
index	testosterone	7703				28414	0.32243	429	0.002111	987e	461
(BMI)	levels	36	T	C	9	625	7	31	61	-30	460
Body	Bioavailabl										
mass	e	rs17						0.01		7.50	
index	testosterone	7883				15648	0.36216	407	0.002055	067e	461
(BMI)	levels	0	A	G	1	9974	5	75	96	-12	460
Body	Bioavailabl										
mass	e	rs17						-0.0		7.70	
index	testosterone	8880				21090		204	0.001982	016e	461
(BMI)	levels	8	G	A	18	023	0.49478	04	55	-25	460
Body	Bioavailabl										
mass	e	rs17						-0.0		4.70	
index	testosterone	9363				13193	0.30914	133	0.002140	002e	461
(BMI)	levels	6	C	G	11	4926	4	343	8	-10	460
Body	Bioavailabl										
mass	e	rs18						-0.0		2.99	
index	testosterone	0512				15064	0.24530	167	0.002297	985e	461
(BMI)	levels	3	G	T	7	5534	1	569	11	-13	460
Body	Bioavailabl										
mass	e	rs18						-0.0		8.60	
index	testosterone	3414				40744	0.37319	140	0.002052	003e	461
(BMI)	levels	4	A	C	18	790	2	112	13	-12	460
Body	Bioavailabl										
mass	e	rs18						-0.0		1.20	
index	testosterone	6141				58933	0.55542	212	0.001989	005e	461
(BMI)	levels	0	T	C	2	591	3	558	18	-26	460
Body	Bioavailabl										
mass	e	rs18						0.02		2.29	
index	testosterone	8489				66128	0.62737	000	0.002056	985e	461
(BMI)	levels	7	G	A	20	32	8	1	34	-22	460
Body	Bioavailabl	rs19				88778	0.48740	0.01	0.002001	5.49	461
mass	e	1924	C	T	5	861	6	167	75	997e	460

index (BMI)	testosterone levels	3						59		-09	
Body mass index (BMI)	Bioavailable testosterone levels	rs1967772	A	G	13	28036062	0.285117	-0.0170434	0.00220377	1e-14	461460
Body mass index (BMI)	Bioavailable testosterone levels	rs2035936	T	G	3	141298124	0.055892	0.0370863	0.00435778	1.69981e-17	461460
Body mass index (BMI)	Bioavailable testosterone levels	rs2051559	C	T	4	3298800	0.13254	0.0204078	0.00291963	2.80027e-12	461460
Body mass index (BMI)	Bioavailable testosterone levels	rs2075466	C	G	16	4872970	0.267182	0.0132903	0.00223627	2.80001e-09	461460
Body mass index (BMI)	Bioavailable testosterone levels	rs2102278	G	A	4	52818664	0.322486	0.0118583	0.0021139	2e-08	461460
Body mass index (BMI)	Bioavailable testosterone levels	rs2133561	T	A	5	139086651	0.611084	-0.014097	0.00204744	5.79963e-12	461460
Body mass index (BMI)	Bioavailable testosterone levels	rs213518	C	T	7	26941065	0.145608	0.0157894	0.00280491	1.79999e-08	461460
Body mass index (BMI)	Bioavailable testosterone levels	rs2153740	G	A	20	2126089	0.479914	-0.0112551	0.00199313	1.6e-08	461460
Body mass index (BMI)	Bioavailable testosterone levels	rs2172131	C	T	10	133978962	0.578723	-0.0149382	0.00200386	8.99912e-14	461460
Body mass index (BMI)	Bioavailable testosterone levels	rs217672	C	A	14	62361021	0.271741	0.0170155	0.00223046	2.39994e-14	461460
Body mass	Bioavailable	rs219215	G	A	4	55505360	0.553308	-0.0150	0.00198302	3.69999e	461460

index (BMI)	testosterone levels	8						12		-14	
Body mass index (BMI)	Bioavailable testosterone levels	rs2216931	A	C	2	181599070	0.661966	0.0169109	0.00208587	5.19996e-16	461460
Body mass index (BMI)	Bioavailable testosterone levels	rs2234458	T	C	11	65639374	0.639519	-0.0203841	0.00205579	3.59998e-23	461460
Body mass index (BMI)	Bioavailable testosterone levels	rs2253310	G	C	6	108888593	0.626099	0.0173213	0.00204115	2.09991e-17	461460
Body mass index (BMI)	Bioavailable testosterone levels	rs2271189	A	G	12	56494991	0.40272	-0.0163086	0.00201869	6.4998e-16	461460
Body mass index (BMI)	Bioavailable testosterone levels	rs2289379	T	C	7	44804225	0.395648	-0.0152765	0.00202952	5.19996e-14	461460
Body mass index (BMI)	Bioavailable testosterone levels	rs2307111	C	T	5	75003678	0.395025	-0.0280042	0.00202203	1.29987e-43	461460
Body mass index (BMI)	Bioavailable testosterone levels	rs2342892	G	T	16	24540806	0.516212	-0.0126972	0.00197715	1.29999e-10	461460
Body mass index (BMI)	Bioavailable testosterone levels	rs2381404	C	T	2	144035442	0.243837	0.0139664	0.00229974	1.29999e-09	461460
Body mass index (BMI)	Bioavailable testosterone levels	rs2383377	A	G	14	33257914	0.130687	0.0161287	0.00293564	3.89996e-08	461460
Body mass index (BMI)	Bioavailable testosterone levels	rs2398861	G	A	9	96430747	0.259169	0.0179932	0.00226733	2.09991e-15	461460
Body mass	Bioavailable	rs242581	A	G	20	44895075	0.415111	0.01222	0.00201106	1.2e-09	461460

index	testosterone	6						84			
(BMI)	levels										
Body	Bioavailabl										
mass	e	rs24						-0.0		3.80	
index	testosterone	3373				23081	0.67765	171	0.002109	014e	461
(BMI)	levels	3	A	G	2	6703	9	805	07	-16	460
Body	Bioavailabl										
mass	e	rs24						0.01		5.30	
index	testosterone	3982				99778	0.54561	919	0.001991	029e	461
(BMI)	levels	3	G	A	10	226	2	98	08	-22	460
Body	Bioavailabl										
mass	e	rs24						-0.0		1.29	
index	testosterone	8235				94178	0.42901	113	0.001995	999e	461
(BMI)	levels	6	C	T	9	371	8	511	06	-08	460
Body	Bioavailabl										
mass	e	rs25						0.01		9.20	
index	testosterone	1289				13145	0.56608	294	0.001998	026e	461
(BMI)	levels	2	C	T	11	1862	5	68	19	-11	460
Body	Bioavailabl										
mass	e							-0.0			
index	testosterone	rs25				77380	0.58796	115	0.002017	1.2e-	461
(BMI)	levels	2761	T	G	5	723	6	046	69	08	460
Body	Bioavailabl										
mass	e	rs25						0.02		1.59	
index	testosterone	6895				72765	0.60365	229	0.002012	993e	461
(BMI)	levels	8	A	G	1	116	6	28	04	-28	460
Body	Bioavailabl										
mass	e	rs25								2.39	
index	testosterone	6999				12926	0.32032	0.01	0.002122	999e	461
(BMI)	levels	3	C	T	3	096	7	267	02	-09	460
Body	Bioavailabl										
mass	e	rs26						-0.0		2.70	
index	testosterone	0622				18353	0.64636	138	0.002083	023e	461
(BMI)	levels	8	C	A	3	7759	8	791	79	-11	460
Body	Bioavailabl										
mass	e	rs26						-0.0		6.70	
index	testosterone	1614				20632	0.31986	138	0.002125	039e	461
(BMI)	levels	3	A	G	8	022	2	738	47	-11	460
Body	Bioavailabl										
mass	e	rs26						0.01		1.39	
index	testosterone	1803				11232	0.38148	439	0.002031	991e	461
(BMI)	levels	9	T	A	1	4111	1	63	7	-12	460
Body	Bioavailabl	rs26				20180	0.34016	0.02	0.002081	3.80	461
mass	e	7820	G	T	1	0511	2	416	58	014e	460

index	testosterone	4						1		-31	
(BMI)	levels										
Body	Bioavailabl										
mass	e	rs27						-0.0		1.10	
index	testosterone	2537				30854	0.69611	160	0.002158	002e	461
(BMI)	levels	1	G	A	8	033	5	301	45	-13	460
Body	Bioavailabl										
mass	e	rs27						-0.0		7.19	
index	testosterone	9164				11207	0.76179	133	0.002313	996e	461
(BMI)	levels	3	T	C	1	269	4	837	32	-09	460
Body	Bioavailabl										
mass	e							-0.0		2.90	
index	testosterone	rs28				42418	0.82068	180	0.002582	001e	461
(BMI)	levels	350	G	A	3	446	4	335	34	-12	460
Body	Bioavailabl										
mass	e	rs28						-0.0		1.59	
index	testosterone	3661				31671	0.13059	264	0.002929	993e	461
(BMI)	levels	56	C	T	6	498	5	826	78	-19	460
Body	Bioavailabl										
mass	e	rs28						0.01		1.09	
index	testosterone	3799				42626	0.65140	266	0.002078	999e	461
(BMI)	levels	6	C	T	21	706	8	47	59	-09	460
Body	Bioavailabl										
mass	e	rs28						-0.0			
index	testosterone	4046				80874	0.36604	117	0.002055	1.2e-	461
(BMI)	levels	39	T	C	5	229	1	129	33	08	460
Body	Bioavailabl										
mass	e	rs28						-0.0		2.80	
index	testosterone	4896				41804	0.29034	153	0.002200	027e	461
(BMI)	levels	20	A	G	22	716	9	737	25	-12	460
Body	Bioavailabl										
mass	e	rs28						-0.0			
index	testosterone	5684				53462	0.10763	182	0.003208	1.2e-	461
(BMI)	levels	18	A	G	15	969	6	825	12	08	460
Body	Bioavailabl										
mass	e	rs28						-0.0			
index	testosterone	6168				67837	0.41198	171	0.001997	1e-1	461
(BMI)	levels	5	C	T	2	553	4	274	63	7	460
Body	Bioavailabl										
mass	e	rs28						-0.0		3.69	
index	testosterone	6706				14036	0.28602	124	0.002264	999e	461
(BMI)	levels	71	C	T	9	3045	4	638	26	-08	460
Body	Bioavailabl	rs28				79403	0.41206	-0.0	0.002019	7.10	461
mass	e	7011	T	C	15	585	6	157	12	068e	460

index (BMI)	testosterone levels	1						151		-15	
Body mass index (BMI)	Bioavailable testosterone levels	rs2875762	C	G	6	124925032	0.242953	0.015339	0.0023121	3.29989e-11	461460
Body mass index (BMI)	Bioavailable testosterone levels	rs2899644	T	C	15	59470366	0.229995	0.0149659	0.0023609	2.30001e-10	461460
Body mass index (BMI)	Bioavailable testosterone levels	rs2920503	T	C	3	12324230	0.285413	-0.0140307	0.00219666	1.7e-10	461460
Body mass index (BMI)	Bioavailable testosterone levels	rs2962334	T	G	5	86879056	0.020065	0.0432552	0.00703547	7.79992e-10	461460
Body mass index (BMI)	Bioavailable testosterone levels	rs317656	A	T	12	69681101	0.724452	-0.0144763	0.00221303	6.09958e-11	461460
Body mass index (BMI)	Bioavailable testosterone levels	rs3213943	A	C	2	136389840	0.131525	-0.0179406	0.00288163	4.79999e-10	461460
Body mass index (BMI)	Bioavailable testosterone levels	rs32421	T	A	5	167362416	0.224315	0.0132315	0.00237757	2.59998e-08	461460
Body mass index (BMI)	Bioavailable testosterone levels	rs329118	T	C	5	133861663	0.419409	-0.0165734	0.0020038	1.29987e-16	461460
Body mass index (BMI)	Bioavailable testosterone levels	rs329651	T	G	11	133767622	0.803977	0.0157218	0.00250202	3.29997e-10	461460
Body mass index (BMI)	Bioavailable testosterone levels	rs34045288	T	C	6	40369081	0.334435	0.0234784	0.00209371	3.50026e-29	461460
Body mass	Bioavailable	rs341530	C	T	15	41339697	0.022158	-0.0389	0.00678155	9.59997e	461460

index	testosterone	25						151		-09	
(BMI)	levels										
Body	Bioavailabl										
mass	e	rs34						-0.0		2.39	
index	testosterone	2342				17516	0.39243	149	0.002040	994e	461
(BMI)	levels	96	A	G	2	6636	7	47	32	-13	460
Body	Bioavailabl										
mass	e	rs34						-0.0		7.80	
index	testosterone	4817				47501	0.16532	185	0.002704	01e-	461
(BMI)	levels	51	A	C	20	038	9	029	04	12	460
Body	Bioavailabl										
mass	e	rs34						0.03		3.59	
index	testosterone	5174				78450	0.12178	884	0.003049	998e	461
(BMI)	levels	39	A	C	1	517	7	8	83	-37	460
Body	Bioavailabl										
mass	e	rs34						0.01		8.10	
index	testosterone	6961				93096	0.47608	143	0.001982	009e	461
(BMI)	levels	81	C	T	7	635	4	45	83	-09	460
Body	Bioavailabl										
mass	e	rs34						-0.0		4.10	
index	testosterone	8114				25408	0.23075	285	0.002342	015e	461
(BMI)	levels	74	A	G	4	838	6	293	69	-34	460
Body	Bioavailabl										
mass	e							-0.0		1.90	
index	testosterone	rs34				84776	0.50038	133	0.001982	02e-	461
(BMI)	levels	9071	A	G	11	849	8	062	31	11	460
Body	Bioavailabl										
mass	e	rs35						-0.0		5.30	
index	testosterone	1543				24862	0.27404	130	0.002234	005e	461
(BMI)	levels	26	G	A	16	414	4	427	42	-09	460
Body	Bioavailabl										
mass	e	rs35						0.02		9.20	
index	testosterone	3644				74278	0.10973	170	0.003183	026e	461
(BMI)	levels	49	T	C	15	126	9	69	35	-12	460
Body	Bioavailabl										
mass	e							0.01		3.29	
index	testosterone	rs35				15403	0.40753	527	0.002013	989e	461
(BMI)	levels	5777	C	G	3	4950	7	15	14	-14	460
Body	Bioavailabl										
mass	e	rs35						-0.0		9.20	
index	testosterone	6975				47298	0.50812	164	0.001980	026e	461
(BMI)	levels	87	A	G	14	505	1	677	67	-17	460
Body	Bioavailabl	rs35				52353	0.08934	0.02	0.003522	6.20	461
mass	e	6976	G	C	15	498	6	303	23	012e	460

index	testosterone	91						05		-11	
(BMI)	levels										
Body	Bioavailabl										
mass	e	rs35						-0.0		9.09	
index	testosterone	8090				47019	0.36320	171	0.002056	913e	461
(BMI)	levels	07	A	G	2	521	5	009	27	-17	460
Body	Bioavailabl										
mass	e	rs35						-0.0		1.10	
index	testosterone	9575				73440	0.57431	196	0.002004	002e	461
(BMI)	levels	44	T	G	8	371	6	429	38	-22	460
Body	Bioavailabl										
mass	e	rs36						-0.0		2.19	
index	testosterone	0076				16300	0.13769	210	0.002867	989e	461
(BMI)	levels	35	A	G	6	9335	8	45	88	-13	460
Body	Bioavailabl										
mass	e	rs36						0.01			
index	testosterone	0619				38329	0.39874	284	0.002019	2e-1	461
(BMI)	levels	54	T	C	8	650	7	45	28	0	460
Body	Bioavailabl										
mass	e	rs37						-0.0		5.19	
index	testosterone	6462				49649	0.58759	117	0.002015	996e	461
(BMI)	levels	5	G	T	19	051	4	714	23	-09	460
Body	Bioavailabl										
mass	e	rs37						-0.0		2.49	
index	testosterone	8471				68072	0.22660	297	0.002360	977e	461
(BMI)	levels	0	C	T	15	458	2	067	42	-36	460
Body	Bioavailabl										
mass	e	rs38						-0.0		6.40	
index	testosterone	0328				10324	0.66681	186	0.002098	03e-	461
(BMI)	levels	6	G	A	14	6470	5	417	28	19	460
Body	Bioavailabl										
mass	e	rs38						-0.0			
index	testosterone	0756				50564		120	0.001995	1.5e-	461
(BMI)	levels	6	T	G	7	204	0.43832	727	83	09	460
Body	Bioavailabl										
mass	e	rs38						0.02			
index	testosterone	1488				29994	0.48240	401	0.001984	1e-3	461
(BMI)	levels	3	T	C	16	922	2	07	24	3	460
Body	Bioavailabl										
mass	e	rs38						-0.0			
index	testosterone	5199				13187	0.74306	136	0.002268	2e-0	461
(BMI)	levels	8	G	C	3	6605	4	071	74	9	460
Body	Bioavailabl	rs38				66574	0.35568	0.01	0.002065	1.2e-	461
mass	e	6680	A	C	1	24	3	178	46	08	460

index (BMI)	testosterone levels	5						35			
Body mass index (BMI)	Bioavailable testosterone levels	rs3897102	T	C	12	123492112	0.411157	0.0120838	0.00202716	2.5e-09	461460
Body mass index (BMI)	Bioavailable testosterone levels	rs3901286	A	C	7	99107727	0.15244	-0.0225553	0.00275602	2.70023e-16	461460
Body mass index (BMI)	Bioavailable testosterone levels	rs3902951	G	T	14	69789755	0.237151	0.0141019	0.00235267	2e-09	461460
Body mass index (BMI)	Bioavailable testosterone levels	rs3935190	A	G	17	79084367	0.536784	-0.0144864	0.00199701	4.00037e-13	461460
Body mass index (BMI)	Bioavailable testosterone levels	rs394608	C	T	21	46581798	0.537697	0.0186353	0.00199484	9.49948e-21	461460
Body mass index (BMI)	Bioavailable testosterone levels	rs40071	C	T	5	107496102	0.179536	-0.0261666	0.00258163	3.80014e-24	461460
Body mass index (BMI)	Bioavailable testosterone levels	rs4017425	T	C	3	44028764	0.470208	-0.0125817	0.00198035	2.1e-10	461460
Body mass index (BMI)	Bioavailable testosterone levels	rs4055791	T	C	13	59266053	0.416804	-0.0178082	0.00200801	7.39946e-19	461460
Body mass index (BMI)	Bioavailable testosterone levels	rs406388	G	C	22	18226997	0.177266	0.015973	0.00260181	8.30004e-10	461460
Body mass index (BMI)	Bioavailable testosterone levels	rs41279738	G	T	1	110082551	0.025988	0.0684263	0.0062225	4.00037e-28	461460
Body mass	Bioavailable e	rs414815	G	A	4	89054667	0.113268	-0.0229	0.00310659	1.39991e	461460

index	testosterone	5						681		-13	
(BMI)	levels										
Body	Bioavailabl										
mass	e	rs42						0.01		1.59	
index	testosterone	6194				31003	0.36493	385	0.002056	993e	461
(BMI)	levels	4	G	T	4	636	5	53	52	-11	460
Body	Bioavailabl										
mass	e	rs42						0.01		1.40	
index	testosterone	6710				60966	0.18634	539	0.002543	001e	461
(BMI)	levels	3	C	T	12	740	7	16	12	-09	460
Body	Bioavailabl										
mass	e	rs42						0.01			
index	testosterone	8460				31843	0.46712	196	0.001995	2e-0	461
(BMI)	levels	0	C	T	15	528	2	73	06	9	460
Body	Bioavailabl										
mass	e							-0.0		3.29	
index	testosterone	rs42				14790	0.57657	173	0.001997	989e	461
(BMI)	levels	9343	G	A	2	3382	3	795	29	-18	460
Body	Bioavailabl										
mass	e							-0.0		2.39	
index	testosterone	rs42				45411	0.15416	266	0.002743	994e	461
(BMI)	levels	9358	C	T	19	941	6	723	73	-22	460
Body	Bioavailabl										
mass	e	rs43						0.01			
index	testosterone	0723				24354	0.45894	214	0.001988	1e-0	461
(BMI)	levels	9	G	A	7	300	1	13	16	9	460
Body	Bioavailabl										
mass	e	rs44						0.01		1.09	
index	testosterone	1947				96150	0.40732	148	0.002010	999e	461
(BMI)	levels	5	T	A	4	044	7	7	6	-08	460
Body	Bioavailabl										
mass	e	rs44						-0.0		2.49	
index	testosterone	4431				92573	0.21614	161	0.002421	977e	461
(BMI)	levels	7	G	A	15	234	1	604	04	-11	460
Body	Bioavailabl										
mass	e	rs44						0.01		3.59	
index	testosterone	5676				25190	0.33344	461	0.002101	998e	461
(BMI)	levels	9	T	C	20	777	4	11	38	-12	460
Body	Bioavailabl										
mass	e	rs44						0.02		2.90	
index	testosterone	7756				54104	0.12864	961	0.002980	001e	461
(BMI)	levels	2	T	C	13	968	4	18	11	-23	460
Body	Bioavailabl	rs44				20537	0.92300	-0.0	0.003706	2.99	461
mass	e	8246	A	C	2	5909	2	313	92	985e	460

index (BMI)	testosterone levels	3						175		-17	
Body mass index (BMI)	Bioavailable testosterone levels	rs45486197	A	G	19	2244849	0.065685	0.025766	0.00403737	1.7e-10	461460
Body mass index (BMI)	Bioavailable testosterone levels	rs4648450	A	C	1	2723214	0.466824	-0.014837	0.00198775	8.4004e-14	461460
Body mass index (BMI)	Bioavailable testosterone levels	rs4672338	T	C	2	60217457	0.336228	0.0135646	0.00208669	8.00018e-11	461460
Body mass index (BMI)	Bioavailable testosterone levels	rs4722398	T	C	7	3125220	0.136137	0.0186993	0.00287545	7.89951e-11	461460
Body mass index (BMI)	Bioavailable testosterone levels	rs4764949	G	A	12	103658096	0.325892	-0.0183924	0.00211197	3.10027e-18	461460
Body mass index (BMI)	Bioavailable testosterone levels	rs4790292	A	C	17	1824305	0.153693	-0.0254509	0.00275603	2.60016e-20	461460
Body mass index (BMI)	Bioavailable testosterone levels	rs4820410	G	A	22	40690385	0.345317	-0.0177457	0.00208527	1.69981e-17	461460
Body mass index (BMI)	Bioavailable testosterone levels	rs4832298	T	C	2	86764004	0.686142	-0.015965	0.00212268	5.40008e-14	461460
Body mass index (BMI)	Bioavailable testosterone levels	rs4876611	G	A	8	116671848	0.720243	0.0197549	0.00220502	3.29989e-19	461460
Body mass index (BMI)	Bioavailable testosterone levels	rs5011579	G	C	16	69187318	0.715152	0.0140268	0.00219322	1.6e-10	461460
Body mass	Bioavailable	rs512121	C	T	18	7548501	0.192054	-0.0159	0.00252176	2.59998e	461460

index (BMI)	testosterone levels							359		-10	
Body mass index (BMI)	Bioavailable testosterone levels	rs529200	G	A	3	173114305	0.527829	0.0168965	0.0019785	1.29987e-17	461460
Body mass index (BMI)	Bioavailable testosterone levels	rs539515	C	A	1	177889025	0.204942	0.0495291	0.0024426	1.99986e-91	461460
Body mass index (BMI)	Bioavailable testosterone levels	rs55714539	C	A	19	18207397	0.343587	0.0175719	0.00210043	6.00067e-17	461460
Body mass index (BMI)	Bioavailable testosterone levels	rs55726687	A	G	12	991306	0.209709	0.02483	0.00242628	1.39991e-24	461460
Body mass index (BMI)	Bioavailable testosterone levels	rs55769038	A	G	11	13331808	0.590384	0.0160961	0.00201096	1.20005e-15	461460
Body mass index (BMI)	Bioavailable testosterone levels	rs558887	G	A	11	28712741	0.307507	-0.0129976	0.00214881	1.5e-09	461460
Body mass index (BMI)	Bioavailable testosterone levels	rs559231	T	G	18	39644247	0.393053	0.0134907	0.00203562	3.40017e-11	461460
Body mass index (BMI)	Bioavailable testosterone levels	rs56038322	A	G	3	69925128	0.31062	0.0139276	0.00214833	8.99912e-11	461460
Body mass index (BMI)	Bioavailable testosterone levels	rs56094641	G	A	16	53806453	0.404564	0.0734967	0.00201412	1e-200	461460
Body mass index (BMI)	Bioavailable testosterone levels	rs56133507	G	T	2	172818467	0.196875	0.0137622	0.00247385	2.69998e-08	461460
Body mass	Bioavailable e	rs561432	T	C	3	157020444	0.256388	0.01266	0.00226533	2.30001e	461460

index	testosterone	36						67		-08	
(BMI)	levels										
Body	Bioavailabl										
mass	e	rs56						0.02		1.39	
index	testosterone	1618				46288	0.13284	245	0.002917	991e	461
(BMI)	levels	55	T	A	17	649	7	75	84	-14	460
Body	Bioavailabl										
mass	e	rs56						0.01		1.40	
index	testosterone	2036				13104	0.14552	797	0.002801	001e	461
(BMI)	levels	22	C	T	9	0874	9	69	83	-10	460
Body	Bioavailabl										
mass	e	rs56						-0.0		2.90	
index	testosterone	3523				19352	0.15489	163	0.002750	001e	461
(BMI)	levels	36	C	T	19	155	4	27	21	-09	460
Body	Bioavailabl										
mass	e	rs56						-0.0		6.40	
index	testosterone	3997				33381	0.44912	161	0.001996	03e-	461
(BMI)	levels	37	T	C	13	721	4	325	46	16	460
Body	Bioavailabl										
mass	e	rs56						0.01		2.39	
index	testosterone	8587				86511	0.29686	591	0.002173	994e	461
(BMI)	levels	68	A	G	13	730	1	88	78	-13	460
Body	Bioavailabl										
mass	e	rs56						0.01		6.19	
index	testosterone	8930				25662	0.30334	251	0.002153	998e	461
(BMI)	levels	62	G	T	8	655	2	41	83	-09	460
Body	Bioavailabl										
mass	e	rs56						0.01		3.69	
index	testosterone	9301				10982	0.13929	575	0.002861	999e	461
(BMI)	levels	05	T	C	2	487	5	57	87	-08	460
Body	Bioavailabl										
mass	e	rs57						-0.0		1.10	
index	testosterone	6363				58048		412	0.003583	002e	461
(BMI)	levels	86	C	T	18	295	0.08383	553	26	-30	460
Body	Bioavailabl										
mass	e	rs57						0.01			
index	testosterone	9897				10062	0.24499	334	0.002363	1.6e-	461
(BMI)	levels	73	C	T	6	9078	5	88	15	08	460
Body	Bioavailabl										
mass	e	rs58						-0.0		2.39	
index	testosterone	8620				75081	0.41926	229	0.002007	994e	461
(BMI)	levels	95	T	C	7	418	6	877	86	-30	460
Body	Bioavailabl	rs59				11325	0.41023	0.01	0.002010	3.79	461
mass	e	0680	T	G	4	6737	2	105	48	997e	460

index	testosterone	84						52		-08	
(BMI)	levels										
Body	Bioavailabl										
mass	e	rs59						0.02		1.50	
index	testosterone	2278				43692		296	0.002153	003e	461
(BMI)	levels	42	G	A	11	423	0.31149	75	13	-26	460
Body	Bioavailabl										
mass	e							-0.0		1.69	
index	testosterone	rs59				69443	0.55416	146	0.001990	981e	461
(BMI)	levels	4024	C	T	11	822	2	771	81	-13	460
Body	Bioavailabl										
mass	e	rs60						-0.0		3.59	
index	testosterone	2365				53479	0.76574	147	0.002349	998e	461
(BMI)	levels	5	G	A	20	658	1	346	97	-10	460
Body	Bioavailabl										
mass	e	rs60								1.10	
index	testosterone	7646				18399	0.14488	0.02	0.002826	002e	461
(BMI)	levels	13	T	G	18	11	3	099	88	-13	460
Body	Bioavailabl										
mass	e	rs61						-0.0		5.60	
index	testosterone	7404				19934	0.23720	135	0.002318	003e	461
(BMI)	levels	66	A	G	1	900	3	153	85	-09	460
Body	Bioavailabl										
mass	e	rs61						0.02		2.80	
index	testosterone	8133				15604	0.13572	902	0.002920	027e	461
(BMI)	levels	24	T	C	1	9877	8	59	38	-23	460
Body	Bioavailabl										
mass	e	rs61						0.02		1.20	
index	testosterone	8286				17432		245	0.003159	005e	461
(BMI)	levels	41	A	G	1	1997	0.10925	85	13	-12	460
Body	Bioavailabl										
mass	e	rs61						-0.0			
index	testosterone	8716				10248	0.09155	267	0.003592	1e-1	461
(BMI)	levels	15	T	C	10	7140	1	159	69	3	460
Body	Bioavailabl										
mass	e	rs61						0.01		2.49	
index	testosterone	9036				89922	0.25496	662	0.002271	977e	461
(BMI)	levels	95	G	A	11	417	4	35	15	-13	460
Body	Bioavailabl										
mass	e	rs61						-0.0		5.10	
index	testosterone	9926				10153		161	0.002069	035e	461
(BMI)	levels	71	G	A	14	1854	0.49195	925	24	-15	460
Body	Bioavailabl	rs62				78029	0.26509	-0.0	0.002242	9.39	461
mass	e	0077	A	G	15	797	1	167	15	94e-	460

index	testosterone	82						007		14	
(BMI)	levels										
Body	Bioavailabl										
mass	e	rs62						-0.0		7.19	
index	testosterone	0207				89960		165	0.002863	996e	461
(BMI)	levels	75	A	T	15	286	0.14168	665	5	-09	460
Body	Bioavailabl										
mass	e	rs62						0.01		2.99	
index	testosterone	0720				52938	0.14454	564	0.002823	999e	461
(BMI)	levels	06	C	A	17	468	1	92	74	-08	460
Body	Bioavailabl										
mass	e	rs62						-0.0		4.60	
index	testosterone	1072				42214	0.04832	911	0.004608	045e	461
(BMI)	levels	61	C	T	2	4	7	559	82	-87	460
Body	Bioavailabl										
mass	e	rs62						-0.0		6.09	
index	testosterone	1762				16619	0.24498	149	0.002288	958e	461
(BMI)	levels	43	T	A	2	0881	7	699	78	-11	460
Body	Bioavailabl										
mass	e	rs62						-0.0			
index	testosterone	1900				18256	0.39044	111	0.002036	4e-0	461
(BMI)	levels	49	C	G	2	6998	6	798	14	8	460
Body	Bioavailabl										
mass	e	rs62						-0.0		5.30	
index	testosterone	2418				20466	0.31444	124	0.002128	005e	461
(BMI)	levels	47	G	A	3	465	3	263	97	-09	460
Body	Bioavailabl										
mass	e	rs62						0.02		1.79	
index	testosterone	2463				94981	0.10236	075	0.003254	999e	461
(BMI)	levels	11	A	G	3	43	4	39	87	-10	460
Body	Bioavailabl										
mass	e	rs62						0.01			
index	testosterone	3792				10587	0.57851	172	0.002005	5e-0	461
(BMI)	levels	71	G	T	5	0033	1	37	57	9	460
Body	Bioavailabl										
mass	e	rs62						0.01		8.49	
index	testosterone	4075				33530	0.26858	443	0.002224	963e	461
(BMI)	levels	62	A	T	6	346	4	93	63	-11	460
Body	Bioavailabl										
mass	e							-0.0		3.29	
index	testosterone	rs62				27679	0.18847	399	0.002527	989e	461
(BMI)	levels	65	T	C	11	916	2	185	19	-56	460
Body	Bioavailabl	rs64				14562	0.10855	0.01	0.003188	5.30	461
mass	e	3006	A	G	2	7927	9	860	2	005e	460

index	testosterone	8						66		-09	
(BMI)	levels										
Body	Bioavailabl										
mass	e	rs64						0.01		8.30	
index	testosterone	4495				17060	0.23747	585	0.002320	042e	461
(BMI)	levels	0	A	G	3	2073	4	25	07	-12	460
Body	Bioavailabl										
mass	e	rs65						-0.0		2.39	
index	testosterone	4571				59307	0.60144	205	0.002015	994e	461
(BMI)	levels	4	A	G	2	725	8	219	73	-24	460
Body	Bioavailabl										
mass	e	rs65						-0.0			
index	testosterone	6090				13341	0.69187	121	0.002141	1.2e-	461
(BMI)	levels	6	C	T	12	4054	4	98	36	08	460
Body	Bioavailabl										
mass	e	rs65						-0.0		4.60	
index	testosterone	6193				58257	0.75358	159	0.002303	045e	461
(BMI)	levels	7	A	T	13	667	7	34	46	-12	460
Body	Bioavailabl										
mass	e	rs65						0.05		2.30	
index	testosterone	6716				57829	0.23271	417	0.002342	144e	461
(BMI)	levels	0	C	T	18	135	4	23	13	-118	460
Body	Bioavailabl										
mass	e	rs65						0.02		8.69	
index	testosterone	7534				94023	0.63603	073	0.002061	961e	461
(BMI)	levels	0	A	G	14	972	8	33	89	-24	460
Body	Bioavailabl										
mass	e	rs66						0.01		6.89	
index	testosterone	6792				18351	0.44582	488	0.001987	922e	461
(BMI)	levels	56	T	C	4	898	6	64	59	-14	460
Body	Bioavailabl										
mass	e	rs66						-0.0		1.59	
index	testosterone	6934				47678	0.58267	170	0.001998	993e	461
(BMI)	levels	1	G	A	1	458	9	287	6	-17	460
Body	Bioavailabl										
mass	e	rs66						0.01		3.69	
index	testosterone	8243				33784	0.67308	315	0.002100	999e	461
(BMI)	levels	8	C	T	1	146	1	86	47	-10	460
Body	Bioavailabl										
mass	e	rs67						-0.0		9.79	
index	testosterone	0556				55320		146	0.002049	941e	461
(BMI)	levels	7	C	T	2	173	0.37596	168	19	-13	460
Body	Bioavailabl	rs67				10012	0.70362	0.01	0.002173	3.89	461
mass	e	0782	G	A	2	3030	7	194	32	996e	460

index	testosterone	7						37		-08	
(BMI)	levels										
Body	Bioavailabl										
mass	e	rs67						-0.0			
index	testosterone	1009				23959	0.34817	116	0.002067	1.6e-	461
(BMI)	levels	1	G	C	2	7	4	835	07	08	460
Body	Bioavailabl										
mass	e	rs67						-0.0		2.19	
index	testosterone	1378				40291	0.40188	135	0.002027	989e	461
(BMI)	levels	1	C	G	2	940	2	736	99	-11	460
Body	Bioavailabl										
mass	e	rs67						0.01		3.29	
index	testosterone	2593				22020	0.84765	910	0.002744	989e	461
(BMI)	levels	1	T	C	2	5146	8	46	03	-12	460
Body	Bioavailabl										
mass	e	rs67						0.05		4.49	
index	testosterone	4464				62850	0.82829	546	0.002612	987e	461
(BMI)	levels	6	G	A	2	4	9	84	13	-100	460
Body	Bioavailabl										
mass	e	rs67						0.01		3.29	
index	testosterone	5297				81741		252	0.002117	997e	461
(BMI)	levels	9	A	G	2	750	0.31688	35	21	-09	460
Body	Bioavailabl										
mass	e	rs67						-0.0		8.10	
index	testosterone	6961				62687		135	0.002089	028e	461
(BMI)	levels	7	T	A	3	746	0.66386	754	02	-11	460
Body	Bioavailabl										
mass	e	rs67						0.01		9.70	
index	testosterone	7489				19611	0.35814	330	0.002056	063e	461
(BMI)	levels	4	A	T	3	6393	8	88	74	-11	460
Body	Bioavailabl										
mass	e	rs67						0.01			
index	testosterone	7778				62376		161	0.002027	1e-0	461
(BMI)	levels	4	T	G	3	645	0.61678	25	53	8	460
Body	Bioavailabl										
mass	e	rs68						-0.0		2.19	
index	testosterone	3108				20257	0.64013	115	0.002059	999e	461
(BMI)	levels	8	A	G	4	769	9	222	1	-08	460
Body	Bioavailabl										
mass	e	rs68						0.01		3.50	
index	testosterone	4385				16213	0.50786	308	0.001976	026e	461
(BMI)	levels	2	T	C	4	2758	1	97	07	-11	460
Body	Bioavailabl	rs69				97753	0.32680	-0.0	0.002113	4.70	461
mass	e	0968	T	C	6	952	9	146	52	002e	460

index	testosterone	5						12		-12	
(BMI)	levels										
Body	Bioavailabl										
mass	e	rs69						0.01			
index	testosterone	2260				14270	0.18984	488	0.002514	3.2e-	461
(BMI)	levels	7	G	A	6	3483	7	48	79	09	460
Body	Bioavailabl										
mass	e	rs69						0.01		1.80	
index	testosterone	3897				98421	0.60145	822	0.002018	011e	461
(BMI)	levels	3	C	T	6	721	6	23	72	-19	460
Body	Bioavailabl										
mass	e	rs69						0.01		2.30	
index	testosterone	5038				12706	0.79508	552	0.002448	001e	461
(BMI)	levels	8	A	G	7	99	6	19	06	-10	460
Body	Bioavailabl										
mass	e	rs69						-0.0			
index	testosterone	6298				11345	0.55601	159	0.001988	1e-1	461
(BMI)	levels	0	C	A	7	2183	9	568	14	5	460
Body	Bioavailabl										
mass	e							-0.0		9.60	
index	testosterone	rs69				35134	0.54359	128	0.001985	064e	461
(BMI)	levels	8147	G	A	5	85	6	512	14	-11	460
Body	Bioavailabl										
mass	e	rs70						0.01		3.10	
index	testosterone	2730				12940	0.65266	454	0.002087	027e	461
(BMI)	levels	4	T	C	9	8290	1	92	03	-12	460
Body	Bioavailabl										
mass	e	rs70						-0.0			
index	testosterone	3455				37081	0.37382	129	0.002042	2.1e-	461
(BMI)	levels	4	G	A	9	301	5	826	52	10	460
Body	Bioavailabl										
mass	e	rs70						-0.0		1.80	
index	testosterone	3894				12037		140	0.002086	011e	461
(BMI)	levels	3	C	T	9	7178	0.33879	202	41	-11	460
Body	Bioavailabl										
mass	e							0.01		1.69	
index	testosterone	rs70				89771		463	0.001985	981e	461
(BMI)	levels	4061	C	T	12	903	0.45505	74	52	-13	460
Body	Bioavailabl										
mass	e	rs70						-0.0		5.99	
index	testosterone	7067				61842	0.32792	123	0.002119	998e	461
(BMI)	levels	0	T	C	10	645	3	274	28	-09	460
Body	Bioavailabl	rs70				13295	0.20573	-0.0	0.002452	6.19	461
mass	e	8125	C	T	10	5696	9	142	16	998e	460

index	testosterone	4						516		-09	
(BMI)	levels										
Body	Bioavailabl										
mass	e	rs71						0.02		1.50	
index	testosterone	2468				47529	0.40835	569	0.002006	003e	461
(BMI)	levels	1	A	C	11	947	3	77	3	-37	460
Body	Bioavailabl										
mass	e	rs71						0.02		1.39	
index	testosterone	3290				50263	0.38445	979	0.002033	991e	461
(BMI)	levels	8	A	G	12	148	8	04	63	-48	460
Body	Bioavailabl										
mass	e	rs71						0.02		6.70	
index	testosterone	4950				33971	0.07693	779	0.003709	039e	461
(BMI)	levels	38	A	G	10	383	6	98	69	-14	460
Body	Bioavailabl										
mass	e	rs72						-0.0		6.00	
index	testosterone	0189				40772	0.35429	149	0.002079	067e	461
(BMI)	levels	5	A	G	16	3	1	781	99	-13	460
Body	Bioavailabl										
mass	e	rs72						0.01		1.79	
index	testosterone	0660				82872		351	0.002117	999e	461
(BMI)	levels	8	G	C	16	628	0.32162	22	97	-10	460
Body	Bioavailabl										
mass	e	rs72						0.01		2.90	
index	testosterone	1801				65832	0.19730	895	0.002492	001e	461
(BMI)	levels	4	C	T	17	016	5	24	16	-14	460
Body	Bioavailabl										
mass	e	rs72						0.01			
index	testosterone	3217				31251		234	0.002008	8e-1	461
(BMI)	levels	1	T	G	18	221	0.58263	04	21	0	460
Body	Bioavailabl										
mass	e							0.01		2.99	
index	testosterone	rs72				21615		112	0.002006	999e	461
(BMI)	levels	3672	T	C	12	61	0.43151	73	98	-08	460
Body	Bioavailabl										
mass	e	rs72						0.01		6.69	
index	testosterone	5083				33937	0.28886	350		993e	461
(BMI)	levels	3	T	C	19	277	6	41	0.002188	-10	460
Body	Bioavailabl										
mass	e	rs72						0.02		6.59	
index	testosterone	5907				47562	0.59606	186	0.002036	933e	461
(BMI)	levels	0	C	T	19	509	2	9	28	-27	460
Body	Bioavailabl	rs72				16010	0.25985	-0.0	0.002280	1e-2	461
mass	e	6348	A	G	1	52	8	212	01	0	460

index	testosterone	26						77			
(BMI)	levels										
Body	Bioavailabl										
mass	e	rs72						0.01			
index	testosterone	6493				80609	0.14318	780	0.002878	6.1e-	461
(BMI)	levels	73	C	T	4	966	8	62	05	10	460
Body	Bioavailabl										
mass	e	rs72						0.02		9.89	
index	testosterone	6739				11888	0.10703	188	0.003214	92e-	461
(BMI)	levels	47	G	A	8	4379	1	64	96	12	460
Body	Bioavailabl										
mass	e	rs72						0.03		1.50	
index	testosterone	8929				50816	0.17223	877	0.002620	003e	461
(BMI)	levels	10	T	G	6	887	2	98	53	-49	460
Body	Bioavailabl										
mass	e	rs72						-0.0		7.59	
index	testosterone	9769				40504	0.19012	232	0.002547	976e	461
(BMI)	levels	86	A	G	19	24	3	257	12	-20	460
Body	Bioavailabl										
mass	e	rs73						-0.0		4.90	
index	testosterone	0267				31017	0.15353	223	0.002750	004e	461
(BMI)	levels	25	A	C	19	686	4	18	52	-16	460
Body	Bioavailabl										
mass	e	rs73						-0.0		7.70	
index	testosterone	0520				18582		303	0.002546	016e	461
(BMI)	levels	33	C	T	3	8465	0.18493	929	34	-33	460
Body	Bioavailabl										
mass	e	rs73						-0.0		3.09	
index	testosterone	1243				71579	0.20514	154	0.002454	999e	461
(BMI)	levels	96	C	T	7	606	4	472	8	-10	460
Body	Bioavailabl										
mass	e	rs73						-0.0		3.59	
index	testosterone	1428				51195	0.19229	266	0.002522	998e	461
(BMI)	levels	79	T	C	20	932	7	936	57	-26	460
Body	Bioavailabl										
mass	e	rs73						-0.0		2.19	
index	testosterone	1937				10829	0.24393	177	0.002320	989e	461
(BMI)	levels	36	G	A	12	4381	4	165	02	-14	460
Body	Bioavailabl										
mass	e	rs73						-0.0		1.69	
index	testosterone	2134				28489	0.14123	225	0.002836	981e	461
(BMI)	levels	84	T	A	4	339	6	766	4	-15	460
Body	Bioavailabl	rs73				99236	0.28527	-0.0	0.002200	6.59	461
mass	e	3142	A	G	13	471	6	143	81	933e	460

index	testosterone	0						702		-11	
(BMI)	levels										
Body	Bioavailabl										
mass	e	rs73						0.01		1.10	
index	testosterone	5775				92207	0.50013	411	0.001983	002e	461
(BMI)	levels	4	G	A	9	308	9	46	62	-12	460
Body	Bioavailabl										
mass	e	rs73						0.01		1.29	
index	testosterone	6015				18549	0.11452	773	0.003116	999e	461
(BMI)	levels	48	T	C	10	889	7	54	39	-08	460
Body	Bioavailabl										
mass	e	rs73						0.01			
index	testosterone	9854				21229	0.30729	366	0.002140	1.7e-	461
(BMI)	levels	39	C	A	2	9249	9	16	59	10	460
Body	Bioavailabl										
mass	e	rs74						-0.0		3.19	
index	testosterone	4288				87682	0.21404	228	0.002412	963e	461
(BMI)	levels	5	G	C	5	877	9	163	51	-21	460
Body	Bioavailabl										
mass	e							0.01		1.10	
index	testosterone	rs74				10546	0.28194	761	0.002197	002e	461
(BMI)	levels	5249	T	C	2	0333	5	18	24	-15	460
Body	Bioavailabl										
mass	e	rs74						-0.0			
index	testosterone	7502				11474	0.08662	196	0.003525	2.5e-	461
(BMI)	levels	82	C	T	7	4463	3	382	61	08	460
Body	Bioavailabl										
mass	e	rs75						0.01		2.59	
index	testosterone	1655				21030	0.39994	200	0.002015	998e	461
(BMI)	levels	4	T	C	1	1331	1	66	65	-09	460
Body	Bioavailabl										
mass	e	rs75						0.01		1.69	
index	testosterone	1925				66434		400	0.001983	981e	461
(BMI)	levels	9	A	G	1	743	0.52836	28	81	-12	460
Body	Bioavailabl										
mass	e							0.02		1.50	
index	testosterone	rs75				42305	0.88658	199	0.003109	003e	461
(BMI)	levels	4635	G	C	3	131	2	25	3	-12	460
Body	Bioavailabl										
mass	e	rs75						-0.0		8.90	
index	testosterone	4995				26145	0.22010	180	0.002418	02e-	461
(BMI)	levels	03	T	C	6	217	1	331	45	14	460
Body	Bioavailabl	rs75				61693	0.26054	-0.0	0.002254	1.80	461
mass	e	7149	G	A	2	51	5	158	54	011e	460

index (BMI)	testosterone levels	6						916		-12	
Body mass index (BMI)	Bioavailable testosterone levels	rs76183894	C	T	3	114371939	0.080747	-0.0219584	0.00364437	1.7e-09	461460
Body mass index (BMI)	Bioavailable testosterone levels	rs7619139	A	T	3	25110415	0.588616	0.0134537	0.00201061	2.19989e-11	461460
Body mass index (BMI)	Bioavailable testosterone levels	rs76702514	G	C	1	195148296	0.210623	-0.0164862	0.00243278	1.20005e-11	461460
Body mass index (BMI)	Bioavailable testosterone levels	rs7683836	A	G	4	180167906	0.557215	-0.0122469	0.00199427	8.19993e-10	461460
Body mass index (BMI)	Bioavailable testosterone levels	rs7708584	G	A	5	153543466	0.572344	-0.0159321	0.0019949	1.39991e-15	461460
Body mass index (BMI)	Bioavailable testosterone levels	rs7761673	A	T	6	70357368	0.21988	-0.0135915	0.0023901	1.29999e-08	461460
Body mass index (BMI)	Bioavailable testosterone levels	rs7762794	G	A	6	153380228	0.285408	0.0149077	0.00218606	9.09913e-12	461460
Body mass index (BMI)	Bioavailable testosterone levels	rs7774	A	C	17	4801163	0.310467	0.014988	0.00215169	3.29989e-12	461460
Body mass index (BMI)	Bioavailable testosterone levels	rs7776021	A	G	6	73742152	0.287599	0.0123701	0.00218251	1.40001e-08	461460
Body mass index (BMI)	Bioavailable testosterone levels	rs78002342	G	T	7	137435925	0.288566	0.0122748	0.00218145	1.79999e-08	461460
Body mass	Bioavailable	rs780544	T	C	7	78121458	0.502261	0.01337	0.00198884	1.80011e	461460

index	testosterone	1						47		-11	
(BMI)	levels										
Body	Bioavailabl										
mass	e	rs78						0.03		3.59	
index	testosterone	0866				24024	0.03983	183	0.005076	998e	461
(BMI)	levels	98	C	T	12	639	8	57	1	-10	460
Body	Bioavailabl										
mass	e							0.01		1.99	
index	testosterone	rs78				53397	0.81254	793	0.002549	986e	461
(BMI)	levels	4257	C	T	18	199	1	15	68	-12	460
Body	Bioavailabl										
mass	e	rs78						-0.0		1.80	
index	testosterone	6058				83631		327	0.004446	011e	461
(BMI)	levels	11	C	A	3	491	0.05405	37	73	-13	460
Body	Bioavailabl										
mass	e	rs78						0.01		2.29	
index	testosterone	9357				16750	0.66589	405	0.002101	985e	461
(BMI)	levels	1	T	G	10	129	7	53	52	-11	460
Body	Bioavailabl										
mass	e	rs79						-0.0		5.10	
index	testosterone	2403				65191	0.50326	142	0.001977	035e	461
(BMI)	levels	6	T	G	10	645	5	836	8	-13	460
Body	Bioavailabl										
mass	e	rs79						0.01		3.29	
index	testosterone	2510				11894	0.39611	472	0.002022	989e	461
(BMI)	levels	0	A	G	11	1596	7	5	1	-13	460
Body	Bioavailabl										
mass	e	rs79						0.01		2.19	
index	testosterone	4478				13079	0.50978	576	0.001987	989e	461
(BMI)	levels	2	G	T	11	5698	4	06	48	-15	460
Body	Bioavailabl										
mass	e	rs79						-0.0		8.30	
index	testosterone	4714				64090	0.16348	182	0.002675	042e	461
(BMI)	levels	3	A	G	11	422	3	782	22	-12	460
Body	Bioavailabl										
mass	e	rs79						0.02			
index	testosterone	7809				10495	0.07742	368	0.003695	1.5e-	461
(BMI)	levels	63	T	C	10	2499	8	03	91	10	460
Body	Bioavailabl										
mass	e	rs79						0.01		3.59	
index	testosterone	9663				97019	0.44935	455	0.002002	998e	461
(BMI)	levels	9	A	G	13	090	4	59	45	-13	460
Body	Bioavailabl	rs80				52862	0.07032	0.02	0.003881	3.79	461
mass	e	1352	T	A	17	77	4	133	13	997e	460

index	testosterone	74						9		-08	
(BMI)	levels										
Body	Bioavailabl										
mass	e	rs80						0.02		6.70	
index	testosterone	1540				25930	0.67709	134	0.002117	039e	461
(BMI)	levels	0	A	C	14	988	7	22	09	-24	460
Body	Bioavailabl										
mass	e	rs80						0.02			
index	testosterone	2036				79937	0.22046	510	0.002395	1e-2	461
(BMI)	levels	5	A	T	14	216	4	68	17	5	460
Body	Bioavailabl										
mass	e	rs80						0.01			
index	testosterone	2413				35837	0.84820	563	0.002767	1.6e-	461
(BMI)	levels	7	T	A	15	297	7	29	78	08	460
Body	Bioavailabl										
mass	e	rs80						-0.0		1.69	
index	testosterone	2551				95271	0.64592	146	0.002075	981e	461
(BMI)	levels	6	G	T	15	872	8	558	78	-12	460
Body	Bioavailabl										
mass	e	rs80						0.01		1.80	
index	testosterone	7666				15888	0.56156	406	0.001996	011e	461
(BMI)	levels	9	C	T	17	448	1	81	01	-12	460
Body	Bioavailabl										
mass	e	rs80						0.01		3.89	
index	testosterone	8951				69224		298	0.002075	996e	461
(BMI)	levels	4	A	T	18	478	0.36868	8	88	-10	460
Body	Bioavailabl										
mass	e	rs81						-0.0		1.80	
index	testosterone	1281				18812	0.40032	206	0.002027	011e	461
(BMI)	levels	8	G	A	19	785	9	96	12	-24	460
Body	Bioavailabl										
mass	e	rs81						-0.0		2.39	
index	testosterone	3249				40288	0.31302	153	0.002193	994e	461
(BMI)	levels	1	A	G	21	577	3	75	17	-12	460
Body	Bioavailabl										
mass	e							-0.0			
index	testosterone	rs81				19029	0.56314	164	0.001985	1e-1	461
(BMI)	levels	5163	C	T	1	4726	8	85	82	6	460
Body	Bioavailabl										
mass	e							-0.0		1.40	
index	testosterone	rs85				17091	0.75857	131	0.002313	001e	461
(BMI)	levels	2042	G	A	20	233	5	207	09	-08	460
Body	Bioavailabl	rs86				69651		-0.0	0.002013	1.20	461
mass	e	2320	T	C	16	866	0.40965	231	45	005e	460

index	testosterone							703		-30	
(BMI)	levels										
Body	Bioavailabl										
mass	e							0.02		2.29	
index	testosterone	rs87				40157	0.61323	411	0.002035	985e	461
(BMI)	levels	9620	T	C	16	29	7	36	95	-32	460
Body	Bioavailabl										
mass	e							-0.0		2.59	
index	testosterone	rs90				41982	0.13481	184	0.002912	998e	461
(BMI)	levels	9892	A	G	20	698	7	105	55	-10	460
Body	Bioavailabl										
mass	e							-0.0		1.29	
index	testosterone	rs92				67802	0.78319	145	0.002402	999e	461
(BMI)	levels	3994	G	A	4	992	5	761	19	-09	460
Body	Bioavailabl										
mass	e	rs92						-0.0		8.60	
index	testosterone	9182				64076	0.51486	142	0.001994	003e	461
(BMI)	levels	2	T	C	5	515	8	63	64	-13	460
Body	Bioavailabl										
mass	e	rs92						0.01			
index	testosterone	9426				83433	0.47655	478	0.001988	1e-1	461
(BMI)	levels	0	A	G	6	228	9	2	18	3	460
Body	Bioavailabl										
mass	e	rs93						0.01		2.59	
index	testosterone	4923				42516	0.41063	118	0.002009	998e	461
(BMI)	levels	5	T	C	6	718	5	09	24	-08	460
Body	Bioavailabl										
mass	e							-0.0		3.19	
index	testosterone	rs93				26949	0.50680	161	0.001972	963e	461
(BMI)	levels	5166	A	G	2	366	5	066	92	-16	460
Body	Bioavailabl										
mass	e	rs94								4.39	
index	testosterone	6317				95100	0.33895	-0.0	0.002101	997e	461
(BMI)	levels	5	T	C	6	30	8	115	15	-08	460
Body	Bioavailabl										
mass	e	rs94						0.01		1.10	
index	testosterone	7849				15433	0.16422	818	0.002674	002e	461
(BMI)	levels	6	C	T	6	3183	5	36	93	-11	460
Body	Bioavailabl										
mass	e	rs95						0.01		3.19	
index	testosterone	1544				11221	0.44770	510	0.001990	963e	461
(BMI)	levels	6	G	A	13	7108	9	51	52	-14	460
Body	Bioavailabl	rs95				11197	0.55339	-0.0	0.001993	1.50	461
mass	e	2218	T	C	13	0212	2	141	25	003e	460

index (BMI)	testosterone levels	0						095		-12	
Body mass index (BMI)	Bioavailable testosterone levels	rs9571687	A	C	13	67472713	0.329363	-0.0135388	0.0021094	1.40001e-10	461460
Body mass index (BMI)	Bioavailable testosterone levels	rs9638713	G	A	7	14645949	0.97477	-0.0361735	0.00635399	1.2e-08	461460
Body mass index (BMI)	Bioavailable testosterone levels	rs9673839	G	A	16	76895693	0.490967	0.0130337	0.00198783	5.50047e-11	461460
Body mass index (BMI)	Bioavailable testosterone levels	rs9674487	G	C	17	80191995	0.001338	0.158445	0.0286171	3.09999e-08	461460
Body mass index (BMI)	Bioavailable testosterone levels	rs9830592	A	C	3	104631603	0.582421	0.0154849	0.00200164	1e-14	461460
Body mass index (BMI)	Bioavailable testosterone levels	rs9839081	A	G	3	123051230	0.325232	-0.011701	0.00214045	4.60002e-08	461460
Body mass index (BMI)	Bioavailable testosterone levels	rs9876664	T	G	3	85806313	0.375389	-0.0180478	0.00204174	9.60064e-19	461460
Body mass index (BMI)	Bioavailable testosterone levels	rs9885533	T	C	13	107854612	0.538079	0.0120038	0.0020182	2.69998e-09	461460
Body mass index (BMI)	Bioavailable testosterone levels	rs9926784	C	T	16	19941968	0.184576	-0.023818	0.00254677	8.60003e-21	461460
Body mass index (BMI)	Bioavailable testosterone levels	rs9951619	G	T	18	56882326	0.767369	0.0144231	0.00235777	9.49992e-10	461460

Supplementary Table S2. Instruments for causal estimation from BMI to estradiol level.

Exposure	Outcome	SNP	Effect allele	Other allele	Chromosome	Genetic position	Effect allele frequency	Beta	Standard error of beta	Pvalue	Sample size
Body mass index (BMI)	Estradiol levels	rs10063055	T	C	5	140990108	0.253365	0.0136775	0.00226991	1.7e-09	461460
Body mass index (BMI)	Estradiol levels	rs10099330	G	A	8	143383694	0.45285	0.0124247	0.00198907	4.20001e-10	461460
Body mass index (BMI)	Estradiol levels	rs10160769	C	G	11	76474827	0.21751	-0.0155862	0.00242199	1.2e-10	461460
Body mass index (BMI)	Estradiol levels	rs10169594	C	T	2	41637688	0.363139	0.0121875	0.00205506	2.99999e-09	461460
Body mass index (BMI)	Estradiol levels	rs10182416	G	A	2	104242992	0.512223	0.0130378	0.00197134	3.69999e-11	461460
Body mass index (BMI)	Estradiol levels	rs10423928	A	T	19	46182304	0.194358	-0.0340136	0.00249869	3.40017e-42	461460
Body mass index (BMI)	Estradiol levels	rs10505836	C	A	12	19288508	0.860012	0.0184851	0.00287086	1.2e-10	461460
Body mass index (BMI)	Estradiol levels	rs10510025	T	C	10	118650996	0.247013	0.0175643	0.00229886	2.19989e-14	461460
Body mass index (BMI)	Estradiol levels	rs1064213	A	G	2	198950240	0.478377	0.0149301	0.00197196	3.69999e-14	461460
Body mass	Estradiol	rs10742	C	T	11	45438374	0.612239	0.0117	0.00202961	6.1e-09	461460

index (BMI)	levels	752						979			
Body mass index (BMI)	Estradiol levels	rs10756714	G	A	9	15885041	0.444196	-0.0207862	0.00199408	1.9002e-25	461460
Body mass index (BMI)	Estradiol levels	rs10756792	T	C	9	16726119	0.742901	-0.0190582	0.00227201	4.90004e-17	461460
Body mass index (BMI)	Estradiol levels	rs10760277	T	C	9	126093999	0.385049	0.0138734	0.00203825	1e-11	461460
Body mass index (BMI)	Estradiol levels	rs10780248	A	G	9	81370555	0.559454	-0.0121232	0.00199415	1.2e-09	461460
Body mass index (BMI)	Estradiol levels	rs1078141	T	C	8	142619393	0.383867	0.0142239	0.00205862	4.90004e-12	461460
Body mass index (BMI)	Estradiol levels	rs10799778	G	T	1	233313353	0.833718	-0.0182237	0.00264876	6.00067e-12	461460
Body mass index (BMI)	Estradiol levels	rs10809621	G	C	9	118599607	0.350143	-0.0125305	0.00207043	1.40001e-09	461460
Body mass index (BMI)	Estradiol levels	rs10824211	T	C	10	76363107	0.139491	0.0207688	0.00286631	4.30031e-13	461460
Body mass index (BMI)	Estradiol levels	rs10832778	G	C	11	17394073	0.623038	0.0115628	0.00203985	1.40001e-08	461460
Body mass index (BMI)	Estradiol levels	rs10927006	C	T	1	243557659	0.143704	-0.0168156	0.00281467	2.30001e-09	461460
Body mass	Estradiol	rs10965	T	C	9	23203619	0.369897	-0.011	0.002052	3.89996	461460

index	level	698						274		e-08	
(BMI)	s							1			
Body	Estra										
mass	diol	rs10				1031		0.0		1.39	
index	level	989				1963	0.3159	169	0.00212	991	461
(BMI)	s	067	A	G	9	4	3	514	349	e-15	460
Body	Estra										
mass	diol	rs11						0.0		7.29	
index	level	001				7876	0.5813	116	0.00201	995	461
(BMI)	s	963	T	C	10	0959	38	794	921	e-09	460
Body	Estra							-0.			
mass	diol	rs11						013		1.40	
index	level	009				3451	0.2440	083	0.00230	001	461
(BMI)	s	685	T	C	10	1990	96	4	766	e-08	460
Body	Estra										
mass	diol	rs11						0.0		7.10	
index	level	012				2183	0.3316	216	0.00210	068	461
(BMI)	s	732	G	A	10	0104	83	425	126	e-25	460
Body	Estra										
mass	diol	rs11						-0.		1.80	
index	level	079				4709	0.3285	020	0.00211	011	461
(BMI)	s	849	T	C	17	0785	31	093	163	e-21	460
Body	Estra							-0.			
mass	diol	rs11				1307		014		5.60	
index	level	099				2490	0.6406	203	0.00206	015	461
(BMI)	s	020	T	C	4	2	09	8	202	e-12	460
Body	Estra							-0.			
mass	diol	rs11						013		2.19	
index	level	115				8242	0.2377	076	0.00233	999	461
(BMI)	s	160	A	G	12	4100	97	8	641	e-08	460
Body	Estra							-0.			
mass	diol	rs11				2303		011		9.09	
index	level	122				0181	0.6117	631	0.00202	997	461
(BMI)	s	450	G	T	1	1	39	6	425	e-09	460
Body	Estra										
mass	diol	rs11				1706		0.0		1.20	
index	level	134				2339	0.6847	182	0.00213	005	461
(BMI)	s	679	G	A	5	1	53	459	286	e-17	460
Body	Estra							-0.			
mass	diol	rs11						021		2.90	
index	level	150				7875	0.3177	161	0.00212	001	461
(BMI)	s	745	G	A	17	7626	11	1	961	e-23	460
Body	Estra	rs11				1716	0.2086	-0.	0.00243	5.39	461
mass	diol	159	T	C	4	3547	71	014	798	995	460

index	level	858				1		224		e-09	
(BMI)	s	5						5			
Body	Estra										
mass	diol	rs11						0.0		4.90	
index	level	165				9692	0.5901	193	0.00200	004	461
(BMI)	s	643	T	C	1	4097	03	319	337	e-22	460
Body	Estra	rs11						-0.			
mass	diol	168						013		4.70	
index	level	938				2717	0.2826	670	0.00219	002	461
(BMI)	s	9	C	G	5	5962	14	6	522	e-10	460
Body	Estra							-0.			
mass	diol	rs11				1219		014		8.40	
index	level	218				2258	0.4004	461	0.00202	04e-	461
(BMI)	s	510	A	G	11	7	7	6	121	13	460
Body	Estra										
mass	diol	rs11						0.0			
index	level	269				4939	0.0352	322	0.00535	1.7e	461
(BMI)	s	30	C	G	12	9132	71	695	892	-09	460
Body	Estra	rs11						-0.			
mass	diol	307				1473		015		5.80	
index	level	957				5408	0.1927	589	0.00251	003	461
(BMI)	s	4	T	C	4	9	9	6	598	e-10	460
Body	Estra	rs11									
mass	diol	360						0.0		1.80	
index	level	386				3956	0.2120	186	0.00242	011	461
(BMI)	s	5	T	C	1	4930	5	013	689	e-14	460
Body	Estra	rs11									
mass	diol	362						0.0			
index	level	410				8832	0.2258	150	0.00236	2.1e	461
(BMI)	s	7	A	G	14	6386	6	461	876	-10	460
Body	Estra							-0.			
mass	diol	rs11				1388		023		6.59	
index	level	525				1719	0.0977	978	0.00333	933	461
(BMI)	s	873	C	T	7	3	01	1	603	e-13	460
Body	Estra										
mass	diol	rs11				1150		0.0		3.10	
index	level	607				3706	0.4865	157	0.00199	027	461
(BMI)	s	476	C	A	11	1	74	12	179	e-15	460
Body	Estra										
mass	diol	rs11				1216		0.0		3.09	
index	level	610				7113	0.1481	164	0.00278	999	461
(BMI)	s	621	A	T	12	3	74	869	318	e-09	460
Body	Estra	rs11				9924	0.2517	-0.	0.00228	2.69	461
mass	diol	630	A	G	15	0947	39	012	082	998	460

index (BMI)	levels	647						674		e-08	
Body mass index (BMI)	Estradiol levels	rs116374395	A	G	5	50723410	0.035444	0.0318817	0.00536236	2.80001e-09	461460
Body mass index (BMI)	Estradiol levels	rs11642090	C	T	16	81730582	0.373536	0.0114184	0.00205925	2.90001e-08	461460
Body mass index (BMI)	Estradiol levels	rs11656076	A	G	17	31464270	0.224774	-0.0154285	0.00237054	7.59976e-11	461460
Body mass index (BMI)	Estradiol levels	rs1167311	A	G	1	49996959	0.681309	-0.0192649	0.00213109	1.59993e-19	461460
Body mass index (BMI)	Estradiol levels	rs11675464	G	A	2	204053742	0.562988	0.0119751	0.00198558	1.6e-09	461460
Body mass index (BMI)	Estradiol levels	rs11691869	A	C	2	100805996	0.362038	-0.0193158	0.00205562	5.60015e-21	461460
Body mass index (BMI)	Estradiol levels	rs11699828	A	G	20	62157198	0.035832	-0.0335418	0.00582677	8.60003e-09	461460
Body mass index (BMI)	Estradiol levels	rs11709402	G	A	3	131551027	0.278732	0.0228192	0.00220796	4.90004e-25	461460
Body mass index (BMI)	Estradiol levels	rs117118217	C	G	10	131783328	0.017711	0.0448186	0.00788004	1.29999e-08	461460
Body mass index (BMI)	Estradiol levels	rs117342986	T	C	16	54267868	0.026415	0.0365794	0.00646781	1.6e-08	461460
Body mass	Estradiol	rs11757	C	T	6	13180454	0.303879	-0.014	0.00214834	9.8992e-	461460

index (BMI)	levels	278						6269		12	
Body mass index (BMI)	Estradiol levels	rs11778219	G	A	8	87762607	0.163123	0.015796	0.0026874	4.20001e-09	461460
Body mass index (BMI)	Estradiol levels	rs118136827	T	G	17	2168104	0.281086	-0.0132582	0.0022019	1.7e-09	461460
Body mass index (BMI)	Estradiol levels	rs11919665	T	A	3	48085349	0.679775	-0.0127854	0.00211292	1.40001e-09	461460
Body mass index (BMI)	Estradiol levels	rs12001437	C	T	9	34074476	0.367678	0.0121836	0.00205069	2.80001e-09	461460
Body mass index (BMI)	Estradiol levels	rs12072739	G	A	1	98315893	0.224478	0.0157008	0.00236691	3.29989e-11	461460
Body mass index (BMI)	Estradiol levels	rs12088284	T	C	1	80798635	0.300838	0.0139317	0.00214854	8.9002e-11	461460
Body mass index (BMI)	Estradiol levels	rs12089815	A	G	1	91189933	0.54861	-0.0123121	0.00198606	5.69994e-10	461460
Body mass index (BMI)	Estradiol levels	rs12140153	T	G	1	62579891	0.094252	-0.033075	0.0034589	1.20005e-21	461460
Body mass index (BMI)	Estradiol levels	rs12149660	A	G	16	70309237	0.114956	-0.0227338	0.00311586	2.99985e-13	461460
Body mass index (BMI)	Estradiol levels	rs12259464	A	G	10	53680099	0.48447	0.0130895	0.00198686	4.49987e-11	461460
Body mass	Estradiol	rs12273	T	C	11	11691101	0.056505	0.0248	0.00428551	6.80002	461460

index (BMI)	levels	545				2		393		e-09	
Body mass index (BMI)	Estradiol levels	rs1229984	C	T	4	100239319	0.972775	0.037357	0.00599385	4.60002e-10	461460
Body mass index (BMI)	Estradiol levels	rs12364470	G	T	11	134601012	0.164556	0.0192705	0.0026658	4.90004e-13	461460
Body mass index (BMI)	Estradiol levels	rs12440603	T	C	15	46585722	0.433693	0.0139285	0.0020011	3.40017e-12	461460
Body mass index (BMI)	Estradiol levels	rs12459368	G	A	19	18459377	0.268203	-0.0170141	0.00223235	2.49977e-14	461460
Body mass index (BMI)	Estradiol levels	rs12462975	A	G	19	30272202	0.329692	0.0195822	0.00212071	2.60016e-20	461460
Body mass index (BMI)	Estradiol levels	rs12541408	C	T	8	95585807	0.31742	-0.0143261	0.00212723	1.59993e-11	461460
Body mass index (BMI)	Estradiol levels	rs1266874	G	A	6	51779638	0.349619	0.0140966	0.0020712	1e-11	461460
Body mass index (BMI)	Estradiol levels	rs12681792	A	C	8	62054463	0.1926	0.0148693	0.00251693	3.50002e-09	461460
Body mass index (BMI)	Estradiol levels	rs12692596	T	C	2	161265910	0.37185	0.0130872	0.00203738	1.29999e-10	461460
Body mass index (BMI)	Estradiol levels	rs12696039	G	A	3	156304750	0.149405	-0.0152802	0.0027705	3.50002e-08	461460
Body mass	Estradiol	rs12860	A	T	14	91458523	0.703747	0.0149	0.00216922	6.20012	461460

index (BMI)	levels	58						126		e-12	
Body mass index (BMI)	Estradiol levels	rs12881629	G	A	14	101146413	0.08265	0.0220748	0.00358929	7.69999e-10	461460
Body mass index (BMI)	Estradiol levels	rs12921986	G	A	16	72312727	0.077984	0.0203327	0.0036983	3.79997e-08	461460
Body mass index (BMI)	Estradiol levels	rs12937411	T	C	17	34950239	0.408167	-0.017187	0.00201383	1.39991e-17	461460
Body mass index (BMI)	Estradiol levels	rs1296328	C	A	4	137083193	0.559032	-0.018862	0.001999	3.90032e-21	461460
Body mass index (BMI)	Estradiol levels	rs12974458	T	C	19	1866115	0.543175	0.0152466	0.00199717	2.29985e-14	461460
Body mass index (BMI)	Estradiol levels	rs13012070	A	G	2	35447243	0.228388	-0.0136501	0.00234795	6.1e-09	461460
Body mass index (BMI)	Estradiol levels	rs13033310	A	G	2	133523605	0.25276	0.012611	0.0022826	3.29997e-08	461460
Body mass index (BMI)	Estradiol levels	rs13097918	A	T	3	35676330	0.212315	0.0145561	0.0024161	1.7e-09	461460
Body mass index (BMI)	Estradiol levels	rs13107325	T	C	4	103188709	0.07492	0.0475799	0.00375479	8.49963e-37	461460
Body mass index (BMI)	Estradiol levels	rs13176429	C	T	5	43152216	0.687609	0.0141555	0.00213122	3.10027e-11	461460
Body mass	Estradiol	rs13202	T	C	17	21264396	0.454791	-0.018	0.00199407	1.50003	461460

index	level	51						032		e-19	
(BMI)	s							1			
Body	Estra							-0.			
mass	diol	rs13				1201		014		5.79	
index	level	218				7350	0.3351	402	0.00209	963	461
(BMI)	s	383	G	C	6	1	32	5	236	e-12	460
Body	Estra							-0.			
mass	diol	rs13						013		1.09	
index	level	228				2048	0.6092	129	0.00203	999	461
(BMI)	s	42	G	A	6	8897	74	2	539	e-10	460
Body	Estra										
mass	diol	rs13						0.0		1.99	
index	level	248				1433	0.2685	157	0.00224	986	461
(BMI)	s	187	C	T	8	6834	57	64	155	e-12	460
Body	Estra							-0.			
mass	diol	rs13						014		2.80	
index	level	272				5117	0.3877	853	0.00203	027	461
(BMI)	s	59	G	A	6	7811	22	2	363	e-13	460
Body	Estra										
mass	diol	rs13						0.0		3.40	
index	level	291				8051	0.5706	110	0.00199	001	461
(BMI)	s	723	A	G	9	0077	83	318	876	e-08	460
Body	Estra							-0.			
mass	diol	rs13						011		3.29	
index	level	301				2776	0.4831	750	0.00198	997	461
(BMI)	s	99	T	G	9	0946	37	9	642	e-09	460
Body	Estra							-0.			
mass	diol	rs13						015		4.60	
index	level	420				5075	0.3650	481	0.00205	045	461
(BMI)	s	048	A	C	2	1414	37	7	272	e-14	460
Body	Estra							-0.			
mass	diol	rs13				2134		018		5.60	
index	level	427				1426	0.2711	151	0.00224	015	461
(BMI)	s	822	G	A	2	5	98	5	142	e-16	460
Body	Estra										
mass	diol	rs13						-0.		9.70	
index	level	468				6565	0.4050	013	0.00201	063	461
(BMI)	s	41	A	G	4	1730	41	056	72	e-11	460
Body	Estra										
mass	diol	rs13						0.0		4.79	
index	level	602				7379	0.4815	130	0.00197	954	461
(BMI)	s	01	T	C	9	6450	46	075	747	e-11	460
Body	Estra	rs13				3043	0.3606	-0.	0.00205	4.30	461
mass	diol	642	T	A	11	2220	54	016	695	031	460

index (BMI)	levels							1363		e-15	
Body mass index (BMI)	Estradiol levels	rs140159717	T	C	15	73765586	0.082292	-0.0246882	0.00370967	2.80027e-11	461460
Body mass index (BMI)	Estradiol levels	rs1438945	A	T	5	152510937	0.715119	-0.0133824	0.00219791	1.09999e-09	461460
Body mass index (BMI)	Estradiol levels	rs1441264	A	G	13	79580919	0.593681	0.0179033	0.00205854	3.40017e-18	461460
Body mass index (BMI)	Estradiol levels	rs1451963	T	G	14	41350367	0.082266	0.022201	0.00360585	7.39997e-10	461460
Body mass index (BMI)	Estradiol levels	rs1458156	T	C	12	41887940	0.488431	0.014075	0.00197951	1.20005e-12	461460
Body mass index (BMI)	Estradiol levels	rs145981104	G	A	8	74714869	0.063711	0.022711	0.00404552	2e-08	461460
Body mass index (BMI)	Estradiol levels	rs146569428	A	G	11	2199686	0.200749	0.0139533	0.00248571	2e-08	461460
Body mass index (BMI)	Estradiol levels	rs1471093	A	G	3	108031094	0.616663	0.0134619	0.00203996	4.10015e-11	461460
Body mass index (BMI)	Estradiol levels	rs1471740	C	T	3	136328270	0.7405	0.0193576	0.00225404	8.9002e-18	461460
Body mass index (BMI)	Estradiol levels	rs147568678	C	T	10	93061851	0.23807	-0.0133327	0.00233034	1.09999e-08	461460
Body mass	Estradiol	rs14772	C	T	5	87988934	0.136947	0.0337	0.00289815	2.19989	461460

index (BMI)	levels	90						772		e-31	
Body mass index (BMI)	Estradiol levels	rs147730268	T	G	12	123024476	0.087243	-0.0350799	0.00358357	1.29987e-22	461460
Body mass index (BMI)	Estradiol levels	rs1503526	C	T	5	63020706	0.480077	0.0154308	0.00197681	5.90065e-15	461460
Body mass index (BMI)	Estradiol levels	rs1562016	C	G	6	104847441	0.753455	0.0131899	0.00228932	8.30004e-09	461460
Body mass index (BMI)	Estradiol levels	rs156914	A	G	1	16848652	0.491786	0.0111552	0.00197308	1.6e-08	461460
Body mass index (BMI)	Estradiol levels	rs1582931	A	G	5	122657199	0.473242	-0.0133419	0.0019956	2.29985e-11	461460
Body mass index (BMI)	Estradiol levels	rs1608113	T	A	3	157815217	0.364998	-0.0117766	0.00205086	9.29994e-09	461460
Body mass index (BMI)	Estradiol levels	rs1609010	G	A	8	77227464	0.565728	0.0209773	0.0019963	7.89951e-26	461460
Body mass index (BMI)	Estradiol levels	rs16916303	G	A	9	30823761	0.119736	-0.0192369	0.00307931	4.20001e-10	461460
Body mass index (BMI)	Estradiol levels	rs17056301	C	T	5	158271680	0.256425	0.0135831	0.0022697	2.19999e-09	461460
Body mass index (BMI)	Estradiol levels	rs17132130	C	G	7	2108036	0.221467	-0.0178418	0.00238564	7.50067e-14	461460
Body mass	Estradiol	rs17149	C	T	7	76634463	0.804854	-0.021	0.00255804	7.00003	461460

index (BMI)	levels	254						3507		e-17	
Body mass index (BMI)	Estradiol levels	rs17289010	G	A	4	140774684	0.32787	-0.0134661	0.00210438	1.6e-10	461460
Body mass index (BMI)	Estradiol levels	rs17399739	G	A	10	87490850	0.068893	0.0270713	0.00391006	4.40048e-12	461460
Body mass index (BMI)	Estradiol levels	rs17446299	G	C	13	40762556	0.165544	0.0153235	0.00266515	8.9e-09	461460
Body mass index (BMI)	Estradiol levels	rs17544384	C	T	1	115295160	0.210864	0.0140931	0.00241434	5.30005e-09	461460
Body mass index (BMI)	Estradiol levels	rs17668356	G	C	3	61208619	0.146029	-0.0230543	0.00279318	1.50003e-16	461460
Body mass index (BMI)	Estradiol levels	rs17770336	T	C	9	28414625	0.322437	0.0242931	0.00211161	1.29987e-30	461460
Body mass index (BMI)	Estradiol levels	rs1778830	A	G	1	156489974	0.362165	0.0140775	0.00205596	7.50067e-12	461460
Body mass index (BMI)	Estradiol levels	rs1788808	G	A	18	21090023	0.49478	-0.020404	0.00198255	7.70016e-25	461460
Body mass index (BMI)	Estradiol levels	rs1793636	C	G	11	131934926	0.309144	-0.0133343	0.0021408	4.70002e-10	461460
Body mass index (BMI)	Estradiol levels	rs1805123	G	T	7	150645534	0.245301	-0.0167569	0.00229711	2.99985e-13	461460
Body mass	Estradiol	rs18341	A	C	18	40744790	0.373192	-0.014	0.00205213	8.60003	461460

index (BMI)	levels	44						0112		e-12	
Body mass index (BMI)	Estradiol levels	rs1861410	T	C	2	58933591	0.555423	-0.0212558	0.00198918	1.20005e-26	461460
Body mass index (BMI)	Estradiol levels	rs1884897	G	A	20	6612832	0.627378	0.020001	0.00205634	2.29985e-22	461460
Body mass index (BMI)	Estradiol levels	rs1919243	C	T	5	88778861	0.487406	0.0116759	0.00200175	5.49997e-09	461460
Body mass index (BMI)	Estradiol levels	rs1967772	A	G	13	28036062	0.285117	-0.0170434	0.00220377	1e-14	461460
Body mass index (BMI)	Estradiol levels	rs2035936	T	G	3	141298124	0.055892	0.0370863	0.00435778	1.69981e-17	461460
Body mass index (BMI)	Estradiol levels	rs2051559	C	T	4	3298800	0.13254	0.0204078	0.00291963	2.80027e-12	461460
Body mass index (BMI)	Estradiol levels	rs2075466	C	G	16	4872970	0.267182	0.0132903	0.00223627	2.80001e-09	461460
Body mass index (BMI)	Estradiol levels	rs2102278	G	A	4	52818664	0.322486	0.0118583	0.0021139	2e-08	461460
Body mass index (BMI)	Estradiol levels	rs2133561	T	A	5	139086651	0.611084	-0.014097	0.00204744	5.79963e-12	461460
Body mass index (BMI)	Estradiol levels	rs213518	C	T	7	26941065	0.145608	0.0157894	0.00280491	1.79999e-08	461460
Body mass	Estradiol	rs21537	G	A	20	2126089	0.479914	-0.011	0.00199313	1.6e-08	461460

index	level	40						255			
(BMI)	s							1			
Body	Estra							-0.			
mass	diol	rs21						015		2.39	
index	level	563				3236	0.6118	522	0.00203	994	461
(BMI)	s	4	G	A	7	9148	79	3	492	e-14	460
Body	Estra							-0.			
mass	diol	rs21				1339		014		8.99	
index	level	721				7896	0.5787	938	0.00200	912	461
(BMI)	s	31	C	T	10	2	23	2	386	e-14	460
Body	Estra										
mass	diol	rs21						0.0		2.39	
index	level	767				6236	0.2717	170	0.00223	994	461
(BMI)	s	2	C	A	14	1021	41	155	046	e-14	460
Body	Estra										
mass	diol	rs21						-0.		3.69	
index	level	921				5550	0.5533	015	0.00198	999	461
(BMI)	s	58	G	A	4	5360	08	012	302	e-14	460
Body	Estra										
mass	diol	rs22				1815		0.0		5.19	
index	level	169				9907	0.6619	169	0.00208	996	461
(BMI)	s	31	A	C	2	0	66	109	587	e-16	460
Body	Estra							-0.			
mass	diol	rs22						020		3.59	
index	level	344				6563	0.6395	384	0.00205	998	461
(BMI)	s	58	T	C	11	9374	19	1	579	e-23	460
Body	Estra										
mass	diol	rs22				1319		0.0		3.79	
index	level	485				2468	0.1645	146	0.00266	997	461
(BMI)	s	51	A	G	6	9	22	523	497	e-08	460
Body	Estra										
mass	diol	rs22				1088		0.0		2.09	
index	level	533				8859	0.6260	173	0.00204	991	461
(BMI)	s	10	G	C	6	3	99	213	115	e-17	460
Body	Estra							-0.			
mass	diol	rs22						016		6.49	
index	level	711				5649	0.4027	308	0.00201	98e-	461
(BMI)	s	89	A	G	12	4991	2	6	869	16	460
Body	Estra							-0.			
mass	diol	rs22						015		5.19	
index	level	893				4480	0.3956	276	0.00202	996	461
(BMI)	s	79	T	C	7	4225	48	5	952	e-14	460
Body	Estra	rs23				7500	0.3950	-0.	0.00202	1.29	461
mass	diol	071	C	T	5	3678	25	028	203	987	460

index (BMI)	levels	11						0042		e-43	
Body mass index (BMI)	Estradiol levels	rs2342892	G	T	16	24540806	0.516212	-0.0126972	0.00197715	1.29999e-10	461460
Body mass index (BMI)	Estradiol levels	rs2381404	C	T	2	144035442	0.243837	0.0139664	0.00229974	1.29999e-09	461460
Body mass index (BMI)	Estradiol levels	rs2383377	A	G	14	33257914	0.130687	0.0161287	0.00293564	3.89996e-08	461460
Body mass index (BMI)	Estradiol levels	rs2398861	G	A	9	96430747	0.259169	0.0179932	0.00226733	2.09991e-15	461460
Body mass index (BMI)	Estradiol levels	rs2425816	A	G	20	44895075	0.415111	0.0122284	0.00201106	1.2e-09	461460
Body mass index (BMI)	Estradiol levels	rs2433733	A	G	2	230816703	0.677659	-0.0171805	0.00210907	3.80014e-16	461460
Body mass index (BMI)	Estradiol levels	rs2439823	G	A	10	99778226	0.545612	0.0191998	0.00199108	5.30029e-22	461460
Body mass index (BMI)	Estradiol levels	rs2482356	C	T	9	94178371	0.429018	-0.0113511	0.00199506	1.29999e-08	461460
Body mass index (BMI)	Estradiol levels	rs2512892	C	T	11	131451862	0.566085	0.0129468	0.00199819	9.20026e-11	461460
Body mass index (BMI)	Estradiol levels	rs252761	T	G	5	77380723	0.587966	-0.0115046	0.00201769	1.2e-08	461460
Body mass	Estradiol	rs25689	A	G	1	72765116	0.603656	0.0222	0.00201204	1.59993	461460

index (BMI)	levels	58						928		e-28	
Body mass index (BMI)	Estradiol levels	rs2569993	C	T	3	12926096	0.320327	0.01267	0.00212202	2.39999e-09	461460
Body mass index (BMI)	Estradiol levels	rs2606228	C	A	3	183537759	0.646368	-0.0138791	0.00208379	2.70023e-11	461460
Body mass index (BMI)	Estradiol levels	rs2616143	A	G	8	20632022	0.319862	-0.0138738	0.00212547	6.70039e-11	461460
Body mass index (BMI)	Estradiol levels	rs2618039	T	A	1	112324111	0.381481	0.0143963	0.0020317	1.39991e-12	461460
Body mass index (BMI)	Estradiol levels	rs2678204	G	T	1	201800511	0.340162	0.024161	0.00208158	3.80014e-31	461460
Body mass index (BMI)	Estradiol levels	rs2725371	G	A	8	30854033	0.696115	-0.0160301	0.00215845	1.10002e-13	461460
Body mass index (BMI)	Estradiol levels	rs2791643	T	C	1	11207269	0.761794	-0.0133837	0.00231332	7.19996e-09	461460
Body mass index (BMI)	Estradiol levels	rs28350	G	A	3	42418446	0.820684	-0.0180335	0.00258234	2.90001e-12	461460
Body mass index (BMI)	Estradiol levels	rs28366156	C	T	6	31671498	0.130595	-0.0264826	0.00292978	1.59993e-19	461460
Body mass index (BMI)	Estradiol levels	rs2837996	C	T	21	42626706	0.651408	0.0126647	0.00207859	1.09999e-09	461460
Body mass	Estradiol	rs28404	T	C	5	80874229	0.366041	-0.011	0.00205533	1.2e-08	461460

index (BMI)	levels	639						7129			
Body mass index (BMI)	Estradiol levels	rs28489620	A	G	22	41804716	0.290349	-0.0153737	0.00220025	2.80027e-12	461460
Body mass index (BMI)	Estradiol levels	rs28568418	A	G	15	53462969	0.107636	-0.0182825	0.00320812	1.2e-08	461460
Body mass index (BMI)	Estradiol levels	rs2861685	C	T	2	67837553	0.411984	-0.0171274	0.00199763	1e-17	461460
Body mass index (BMI)	Estradiol levels	rs28670671	C	T	9	140363045	0.286024	-0.0124638	0.00226426	3.69999e-08	461460
Body mass index (BMI)	Estradiol levels	rs2870111	T	C	15	79403585	0.412066	-0.0157151	0.00201912	7.10068e-15	461460
Body mass index (BMI)	Estradiol levels	rs2875762	C	G	6	124925032	0.242953	0.015339	0.0023121	3.29989e-11	461460
Body mass index (BMI)	Estradiol levels	rs2899644	T	C	15	59470366	0.229995	0.0149659	0.0023609	2.30001e-10	461460
Body mass index (BMI)	Estradiol levels	rs2920503	T	C	3	12324230	0.285413	-0.0140307	0.00219666	1.7e-10	461460
Body mass index (BMI)	Estradiol levels	rs2962334	T	G	5	86879056	0.020065	0.0432552	0.00703547	7.79992e-10	461460
Body mass index (BMI)	Estradiol levels	rs3176556	A	T	12	69681101	0.724452	-0.0144763	0.00221303	6.09958e-11	461460
Body mass	Estradiol	rs32139	A	C	2	13638984	0.131525	-0.017	0.00288163	4.79999	461460

index (BMI)	levels	43				0		9406		e-10	
Body mass index (BMI)	Estradiol levels	rs32421	T	A	5	167362416	0.224315	0.0132315	0.00237757	2.59998e-08	461460
Body mass index (BMI)	Estradiol levels	rs329118	T	C	5	133861663	0.419409	-0.0165734	0.0020038	1.29987e-16	461460
Body mass index (BMI)	Estradiol levels	rs329651	T	G	11	133767622	0.803977	0.0157218	0.00250202	3.29997e-10	461460
Body mass index (BMI)	Estradiol levels	rs34045288	T	C	6	40369081	0.334435	0.0234784	0.00209371	3.50026e-29	461460
Body mass index (BMI)	Estradiol levels	rs34153025	C	T	15	41339697	0.022158	-0.0389151	0.00678155	9.59997e-09	461460
Body mass index (BMI)	Estradiol levels	rs34234296	A	G	2	175166636	0.392437	-0.014947	0.00204032	2.39994e-13	461460
Body mass index (BMI)	Estradiol levels	rs34481751	A	C	20	47501038	0.165329	-0.0185029	0.00270404	7.8001e-12	461460
Body mass index (BMI)	Estradiol levels	rs34517439	A	C	1	78450517	0.121787	0.038848	0.00304983	3.59998e-37	461460
Body mass index (BMI)	Estradiol levels	rs34696181	C	T	7	93096635	0.476084	0.0114345	0.00198283	8.10009e-09	461460
Body mass index (BMI)	Estradiol levels	rs34811474	A	G	4	25408838	0.230756	-0.0285293	0.00234269	4.10015e-34	461460
Body mass	Estradiol	rs34907	A	G	11	84776849	0.500388	-0.013	0.00198231	1.9002e-	461460

index (BMI)	levels	1						3062		11	
Body mass index (BMI)	Estradiol levels	rs35154326	G	A	16	24862414	0.274044	-0.0130420427	0.00223442	5.30005e-09	461460
Body mass index (BMI)	Estradiol levels	rs35364449	T	C	15	74278126	0.109739	0.0217069	0.00318335	9.20026e-12	461460
Body mass index (BMI)	Estradiol levels	rs355777	C	G	3	154034950	0.407537	0.0152715	0.00201314	3.29989e-14	461460
Body mass index (BMI)	Estradiol levels	rs35697587	A	G	14	47298505	0.508121	-0.0164677	0.00198067	9.20026e-17	461460
Body mass index (BMI)	Estradiol levels	rs35697691	G	C	15	52353498	0.089346	0.0230305	0.00352223	6.20012e-11	461460
Body mass index (BMI)	Estradiol levels	rs35809007	A	G	2	47019521	0.363205	-0.0171009	0.00205627	9.09913e-17	461460
Body mass index (BMI)	Estradiol levels	rs35957544	T	G	8	73440371	0.574316	-0.0196429	0.00200438	1.10002e-22	461460
Body mass index (BMI)	Estradiol levels	rs36007635	A	G	6	163009335	0.137698	-0.021045	0.00286788	2.19989e-13	461460
Body mass index (BMI)	Estradiol levels	rs36061954	T	C	8	38329650	0.398747	0.0128445	0.00201928	2e-10	461460
Body mass index (BMI)	Estradiol levels	rs3764625	G	T	19	49649051	0.587594	-0.0117714	0.00201523	5.19996e-09	461460
Body mass	Estradiol	rs37847	C	T	15	68072458	0.226602	-0.029	0.00236042	2.49977	461460

index (BMI)	levels	10						7067		e-36	
Body mass index (BMI)	Estradiol levels	rs3803286	G	A	14	103246470	0.666815	-0.0186417	0.00209828	6.4003e-19	461460
Body mass index (BMI)	Estradiol levels	rs3807566	T	G	7	50564204	0.43832	-0.0120727	0.00199583	1.5e-09	461460
Body mass index (BMI)	Estradiol levels	rs3814883	T	C	16	29994922	0.482402	0.0240107	0.00198424	1e-33	461460
Body mass index (BMI)	Estradiol levels	rs3845344	T	C	1	75001480	0.391115	0.0163609	0.00201927	5.40008e-16	461460
Body mass index (BMI)	Estradiol levels	rs3851998	G	C	3	131876605	0.743064	-0.0136071	0.00226874	2e-09	461460
Body mass index (BMI)	Estradiol levels	rs3866805	A	C	1	6657424	0.355683	0.0117835	0.00206546	1.2e-08	461460
Body mass index (BMI)	Estradiol levels	rs3897102	T	C	12	123492112	0.411157	0.0120838	0.00202716	2.5e-09	461460
Body mass index (BMI)	Estradiol levels	rs3902951	G	T	14	69789755	0.237151	0.0141019	0.00235267	2e-09	461460
Body mass index (BMI)	Estradiol levels	rs3935190	A	G	17	79084367	0.536784	-0.0144864	0.00199701	4.00037e-13	461460
Body mass index (BMI)	Estradiol levels	rs394608	C	T	21	46581798	0.537697	0.0186353	0.00199484	9.49948e-21	461460
Body mass	Estradiol	rs40071	C	T	5	10749610	0.179536	-0.026	0.00258163	3.80014	461460

index	level					2		166		e-24	
(BMI)	s							6			
Body	Estra							-0.			
mass	diol	rs40						012			
index	level	174				4402	0.4702	581	0.00198	2.1e	461
(BMI)	s	25	T	C	3	8764	08	7	035	-10	460
Body	Estra							-0.			
mass	diol	rs40						017		7.39	
index	level	557				5926	0.4168	808	0.00200	946	461
(BMI)	s	91	T	C	13	6053	04	2	801	e-19	460
Body	Estra										
mass	diol	rs40						0.0		8.30	
index	level	638				1822	0.1772	159	0.00260	004	461
(BMI)	s	8	G	C	22	6997	66	73	181	e-10	460
Body	Estra										
mass	diol	rs41				1100		0.0		4.00	
index	level	279				8255	0.0259	684	0.00622	037	461
(BMI)	s	738	G	T	1	1	88	263	25	e-28	460
Body	Estra							-0.			
mass	diol	rs41						022		1.39	
index	level	481				8905	0.1132	968	0.00310	991	461
(BMI)	s	55	G	A	4	4667	68	1	659	e-13	460
Body	Estra										
mass	diol	rs42						0.0		1.59	
index	level	619				3100	0.3649	138	0.00205	993	461
(BMI)	s	44	G	T	4	3636	35	553	652	e-11	460
Body	Estra										
mass	diol	rs42						0.0		1.40	
index	level	671				6096	0.1863	153	0.00254	001	461
(BMI)	s	03	C	T	12	6740	47	916	312	e-09	460
Body	Estra										
mass	diol	rs42						0.0			
index	level	846				3184	0.4671	119	0.00199	2e-0	461
(BMI)	s	00	C	T	15	3528	22	673	506	9	460
Body	Estra							-0.			
mass	diol	rs42				1479		017		3.29	
index	level	934				0338	0.5765	379	0.00199	989	461
(BMI)	s	3	G	A	2	2	73	5	729	e-18	460
Body	Estra							-0.			
mass	diol	rs42						026		2.39	
index	level	935				4541	0.1541	672	0.00274	994	461
(BMI)	s	8	C	T	19	1941	66	3	373	e-22	460
Body	Estra	rs43				2435	0.4589	0.0	0.00198	1e-0	461
mass	diol	072	G	A	7	4300	41	121	816	9	460

index (BMI)	levels	39						413			
Body mass index (BMI)	Estradiol levels	rs4419475	T	A	4	96150044	0.407327	0.011487	0.0020106	1.09999e-08	461460
Body mass index (BMI)	Estradiol levels	rs4444317	G	A	15	92573234	0.216141	-0.0161604	0.00242104	2.49977e-11	461460
Body mass index (BMI)	Estradiol levels	rs4456769	T	C	20	25190777	0.333444	0.0146111	0.00210138	3.59998e-12	461460
Body mass index (BMI)	Estradiol levels	rs4477562	T	C	13	54104968	0.128644	0.0296118	0.00298011	2.90001e-23	461460
Body mass index (BMI)	Estradiol levels	rs4482463	A	C	2	205375909	0.923002	-0.0313175	0.00370692	2.99985e-17	461460
Body mass index (BMI)	Estradiol levels	rs45486197	A	G	19	2244849	0.065685	0.025766	0.00403737	1.7e-10	461460
Body mass index (BMI)	Estradiol levels	rs4605363	C	A	2	229010960	0.341595	0.0163638	0.00207733	3.29989e-15	461460
Body mass index (BMI)	Estradiol levels	rs4648450	A	C	1	2723214	0.466824	-0.0148837	0.00198775	8.4004e-14	461460
Body mass index (BMI)	Estradiol levels	rs4658403	T	C	1	243832560	0.833659	-0.0188877	0.00264997	1e-12	461460
Body mass index (BMI)	Estradiol levels	rs4672338	T	C	2	60217457	0.336228	0.0135646	0.00208669	8.00018e-11	461460
Body mass	Estradiol	rs47223	T	C	7	3125220	0.136137	0.0186	0.00287545	7.89951	461460

index	level	98						993		e-11	
(BMI)	s										
Body	Estra							-0.			
mass	diol	rs47				1036		018		3.10	
index	level	649				5809	0.3258	392	0.00211	027	461
(BMI)	s	49	G	A	12	6	92	4	197	e-18	460
Body	Estra							-0.			
mass	diol	rs47						025		2.60	
index	level	902				1824	0.1536	450	0.00275	016	461
(BMI)	s	92	A	C	17	305	93	9	603	e-20	460
Body	Estra							-0.			
mass	diol	rs48						017		1.69	
index	level	204				4069	0.3453	745	0.00208	981	461
(BMI)	s	10	G	A	22	0385	17	7	527	e-17	460
Body	Estra										
mass	diol	rs48						-0.		5.40	
index	level	322				8676	0.6861	015	0.00212	008	461
(BMI)	s	98	T	C	2	4004	42	965	268	e-14	460
Body	Estra										
mass	diol	rs48						0.0		1.39	
index	level	589				8825	0.8855	229	0.00309	991	461
(BMI)	s	40	C	T	3	4820	69	281	976	e-13	460
Body	Estra										
mass	diol	rs48				1166		0.0		3.29	
index	level	766				7184	0.7202	197	0.00220	989	461
(BMI)	s	11	G	A	8	8	43	549	502	e-19	460
Body	Estra										
mass	diol	rs49						0.0		4.49	
index	level	299				8639	0.6452	189	0.00206	987	461
(BMI)	s	23	C	T	11	200	04	424	436	e-20	460
Body	Estra										
mass	diol	rs50						0.0			
index	level	115				6918	0.7151	140	0.00219	1.6e	461
(BMI)	s	79	G	C	16	7318	52	268	322	-10	460
Body	Estra							-0.			
mass	diol	rs51						015		2.59	
index	level	212				7548	0.1920	935	0.00252	998	461
(BMI)	s	1	C	T	18	501	54	9	176	e-10	460
Body	Estra										
mass	diol	rs52				1731		0.0		1.29	
index	level	920				1430	0.5278	168	0.00197	987	461
(BMI)	s	0	G	A	3	5	29	965	85	e-17	460
Body	Estra	rs53				1778	0.2049	0.0	0.00244	1.99	461
mass	diol	951	C	A	1	8902	42	495	26	986	460

index (BMI)	levels	5				5		291		e-91	
Body mass index (BMI)	Estradiol levels	rs55707359	G	T	11	46159333	0.015432	0.0531292	0.00812381	6.20012e-11	461460
Body mass index (BMI)	Estradiol levels	rs55714539	C	A	19	18207397	0.343587	0.0175719	0.00210043	6.00067e-17	461460
Body mass index (BMI)	Estradiol levels	rs55726687	A	G	12	991306	0.209709	0.02483	0.00242628	1.39991e-24	461460
Body mass index (BMI)	Estradiol levels	rs55769038	A	G	11	13331808	0.590384	0.0160961	0.00201096	1.20005e-15	461460
Body mass index (BMI)	Estradiol levels	rs558887	G	A	11	28712741	0.307507	-0.0129976	0.00214881	1.5e-09	461460
Body mass index (BMI)	Estradiol levels	rs559231	T	G	18	39644247	0.393053	0.0134907	0.00203562	3.40017e-11	461460
Body mass index (BMI)	Estradiol levels	rs56038322	A	G	3	69925128	0.31062	0.0139276	0.00214833	8.99912e-11	461460
Body mass index (BMI)	Estradiol levels	rs56094641	G	A	16	53806453	0.404564	0.0734967	0.00201412	1e-200	461460
Body mass index (BMI)	Estradiol levels	rs56133507	G	T	2	172818467	0.196875	0.0137622	0.00247385	2.69998e-08	461460
Body mass index (BMI)	Estradiol levels	rs56143236	T	C	3	157020444	0.256388	0.0126667	0.00226533	2.30001e-08	461460
Body mass	Estradiol	rs56161	T	A	17	46288649	0.132847	0.0224	0.00291784	1.39991	461460

index (BMI)	levels	855						575		e-14	
Body mass index (BMI)	Estradiol levels	rs56203622	C	T	9	131040874	0.145529	0.0179769	0.00280183	1.40001e-10	461460
Body mass index (BMI)	Estradiol levels	rs56352336	C	T	19	19352155	0.154894	-0.016327	0.00275021	2.90001e-09	461460
Body mass index (BMI)	Estradiol levels	rs56399737	T	C	13	33381721	0.449124	-0.0161325	0.00199646	6.4003e-16	461460
Body mass index (BMI)	Estradiol levels	rs56858768	A	G	13	86511730	0.296861	0.0159188	0.00217378	2.39994e-13	461460
Body mass index (BMI)	Estradiol levels	rs56893062	G	T	8	25662655	0.303342	0.0125141	0.00215383	6.19998e-09	461460
Body mass index (BMI)	Estradiol levels	rs56930105	T	C	2	10982487	0.139295	0.0157557	0.00286187	3.69999e-08	461460
Body mass index (BMI)	Estradiol levels	rs57636386	C	T	18	58048295	0.08383	-0.0412553	0.00358326	1.10002e-30	461460
Body mass index (BMI)	Estradiol levels	rs57989773	C	T	6	100629078	0.244995	0.0133488	0.00236315	1.6e-08	461460
Body mass index (BMI)	Estradiol levels	rs58862095	T	C	7	75081418	0.419266	-0.0229877	0.00200786	2.39994e-30	461460
Body mass index (BMI)	Estradiol levels	rs59068084	T	G	4	113256737	0.410232	0.0110552	0.00201048	3.79997e-08	461460
Body mass	Estradiol	rs59227	G	A	11	43692423	0.31149	0.0229	0.00215313	1.50003	461460

index (BMI)	levels	842						675		e-26	
Body mass index (BMI)	Estradiol levels	rs594024	C	T	11	69443822	0.554162	-0.0146771	0.00199081	1.69981e-13	461460
Body mass index (BMI)	Estradiol levels	rs6023655	G	A	20	53479658	0.765741	-0.0147346	0.00234997	3.59998e-10	461460
Body mass index (BMI)	Estradiol levels	rs60764613	T	G	18	1839911	0.144883	0.02099	0.00282688	1.10002e-13	461460
Body mass index (BMI)	Estradiol levels	rs61740466	A	G	1	19934900	0.237203	-0.0135153	0.00231885	5.60003e-09	461460
Body mass index (BMI)	Estradiol levels	rs61813324	T	C	1	156049877	0.135728	0.0290259	0.00292038	2.80027e-23	461460
Body mass index (BMI)	Estradiol levels	rs61828641	A	G	1	174321997	0.10925	0.0224585	0.00315913	1.20005e-12	461460
Body mass index (BMI)	Estradiol levels	rs61871615	T	C	10	102487140	0.091551	-0.0267159	0.00359269	1e-13	461460
Body mass index (BMI)	Estradiol levels	rs61903695	G	A	11	89922417	0.254964	0.0166235	0.00227115	2.49977e-13	461460
Body mass index (BMI)	Estradiol levels	rs61992671	G	A	14	101531854	0.49195	-0.0161925	0.00206924	5.10035e-15	461460
Body mass index (BMI)	Estradiol levels	rs62007782	A	G	15	78029797	0.265091	-0.0167007	0.00224215	9.3994e-14	461460
Body mass	Estradiol	rs62020	A	T	15	89960286	0.14168	-0.016	0.0028635	7.19996	461460

index (BMI)	levels	775						5665		e-09	
Body mass index (BMI)	Estradiol levels	rs62072006	C	A	17	52938468	0.144541	0.0156492	0.00282374	2.99999e-08	461460
Body mass index (BMI)	Estradiol levels	rs62107261	C	T	2	422144	0.048327	-0.0911559	0.00460882	4.60045e-87	461460
Body mass index (BMI)	Estradiol levels	rs62176243	T	A	2	166190881	0.244987	-0.0149699	0.00228878	6.09958e-11	461460
Body mass index (BMI)	Estradiol levels	rs62190049	C	G	2	182566998	0.390446	-0.0111798	0.00203614	4e-08	461460
Body mass index (BMI)	Estradiol levels	rs62241847	G	A	3	20466465	0.314443	-0.0124263	0.00212897	5.30005e-09	461460
Body mass index (BMI)	Estradiol levels	rs62246311	A	G	3	9498143	0.102364	0.0207539	0.00325487	1.79999e-10	461460
Body mass index (BMI)	Estradiol levels	rs62379271	G	T	5	105870033	0.578511	0.0117237	0.00200557	5e-09	461460
Body mass index (BMI)	Estradiol levels	rs62407562	A	T	6	33530346	0.268584	0.0144393	0.00222463	8.49963e-11	461460
Body mass index (BMI)	Estradiol levels	rs6265	T	C	11	27679916	0.188472	-0.0399185	0.00252719	3.29989e-56	461460
Body mass index (BMI)	Estradiol levels	rs6430068	A	G	2	145627927	0.108559	0.0186066	0.0031882	5.30005e-09	461460
Body mass	Estradiol	rs64449	A	G	3	17060207	0.237474	0.0158	0.00232007	8.30042	461460

index (BMI)	levels	50				3		525		e-12	
Body mass index (BMI)	Estradiol levels	rs6545714	A	G	2	59307725	0.601448	-0.0205219	0.00201573	2.39994e-24	461460
Body mass index (BMI)	Estradiol levels	rs6560906	C	T	12	133414054	0.691874	-0.012198	0.00214136	1.2e-08	461460
Body mass index (BMI)	Estradiol levels	rs6561937	A	T	13	58257667	0.753587	-0.015934	0.00230346	4.60045e-12	461460
Body mass index (BMI)	Estradiol levels	rs6567160	C	T	18	57829135	0.232714	0.0541723	0.00234213	2.30144e-118	461460
Body mass index (BMI)	Estradiol levels	rs6575340	A	G	14	94023972	0.636038	0.0207333	0.00206189	8.69961e-24	461460
Body mass index (BMI)	Estradiol levels	rs66679256	T	C	4	18351898	0.445826	0.0148864	0.00198759	6.89922e-14	461460
Body mass index (BMI)	Estradiol levels	rs6669341	G	A	1	47678458	0.582679	-0.0170287	0.0019986	1.59993e-17	461460
Body mass index (BMI)	Estradiol levels	rs6682438	C	T	1	33784146	0.673081	0.0131586	0.00210047	3.69999e-10	461460
Body mass index (BMI)	Estradiol levels	rs6705567	C	T	2	55320173	0.37596	-0.0146168	0.00204919	9.79941e-13	461460
Body mass index (BMI)	Estradiol levels	rs6707827	G	A	2	100123030	0.703627	0.0119437	0.00217332	3.89996e-08	461460
Body mass	Estradiol	rs67100	G	C	2	239597	0.348174	-0.011	0.00206707	1.6e-08	461460

index	level	91						683			
(BMI)	s							5			
Body	Estra							-0.			
mass	diol	rs67						013		2.19	
index	level	137				4029	0.4018	573	0.00202	989	461
(BMI)	s	81	C	G	2	1940	82	6	799	e-11	460
Body	Estra										
mass	diol	rs67				2202		0.0		3.29	
index	level	259				0514	0.8476	191	0.00274	989	461
(BMI)	s	31	T	C	2	6	58	046	403	e-12	460
Body	Estra									4.49	
mass	diol	rs67						0.0		987	
index	level	446				6285	0.8282	554	0.00261	e-10	461
(BMI)	s	46	G	A	2	04	99	684	213	0	460
Body	Estra										
mass	diol	rs67						0.0		3.29	
index	level	529				8174	0.3168	125	0.00211	997	461
(BMI)	s	79	A	G	2	1750	8	235	721	e-09	460
Body	Estra										
mass	diol	rs67				1266		0.0		8.60	
index	level	609				4093	0.2836	170	0.00220	003	461
(BMI)	s	008	C	T	10	6	14	863	21	e-15	460
Body	Estra							-0.			
mass	diol	rs67						013		8.10	
index	level	696				6268	0.6638	575	0.00208	028	461
(BMI)	s	17	T	A	3	7746	6	4	902	e-11	460
Body	Estra										
mass	diol	rs67				1961		0.0		9.70	
index	level	748				1639	0.3581	133	0.00205	063	461
(BMI)	s	94	A	T	3	3	48	088	674	e-11	460
Body	Estra										
mass	diol	rs67						0.0			
index	level	777				6237	0.6167	116	0.00202	1e-0	461
(BMI)	s	84	T	G	3	6645	8	125	753	8	460
Body	Estra							-0.			
mass	diol	rs68						011		2.19	
index	level	310				2025	0.6401	522	0.00205	999	461
(BMI)	s	88	A	G	4	7769	39	2	91	e-08	460
Body	Estra										
mass	diol	rs68				1621		0.0		3.50	
index	level	438				3275	0.5078	130	0.00197	026	461
(BMI)	s	52	T	C	4	8	61	897	607	e-11	460
Body	Estra	rs69				9775	0.3268	-0.	0.00211	4.70	461
mass	diol	096	T	C	6	3952	09	014	352	002	460

index (BMI)	levels	85						612		e-12	
Body mass index (BMI)	Estradiol levels	rs6922607	G	A	6	142703483	0.189847	0.0148848	0.00251479	3.2e-09	461460
Body mass index (BMI)	Estradiol levels	rs6938973	C	T	6	98421721	0.601456	0.0182223	0.00201872	1.80011e-19	461460
Body mass index (BMI)	Estradiol levels	rs6950388	A	G	7	1270699	0.795086	0.0155219	0.00244806	2.30001e-10	461460
Body mass index (BMI)	Estradiol levels	rs6962980	C	A	7	113452183	0.556019	-0.0159568	0.00198814	1e-15	461460
Body mass index (BMI)	Estradiol levels	rs698147	G	A	5	3513485	0.543596	-0.0128512	0.00198514	9.60064e-11	461460
Body mass index (BMI)	Estradiol levels	rs7024334	G	T	9	109072075	0.77914	-0.0138177	0.00238338	6.69993e-09	461460
Body mass index (BMI)	Estradiol levels	rs7027304	T	C	9	129408290	0.652661	0.0145492	0.00208703	3.10027e-12	461460
Body mass index (BMI)	Estradiol levels	rs7034554	G	A	9	37081301	0.373825	-0.0129826	0.00204252	2.1e-10	461460
Body mass index (BMI)	Estradiol levels	rs7038943	C	T	9	120377178	0.338789	-0.0140202	0.00208641	1.80011e-11	461460
Body mass index (BMI)	Estradiol levels	rs704061	C	T	12	89771903	0.45505	0.0146374	0.00198552	1.69981e-13	461460
Body mass	Estradiol	rs70706	T	C	10	61842645	0.327923	-0.012	0.00211928	5.99998	461460

index	level	70						327		e-09	
(BMI)	s							4			
Body	Estra							-0.			
mass	diol	rs70				1329		014		6.19	
index	level	812				5569	0.2057	251	0.00245	998	461
(BMI)	s	54	C	T	10	6	39	6	216	e-09	460
Body	Estra										
mass	diol	rs71						0.0		1.50	
index	level	246				4752	0.4083	256	0.00200	003	461
(BMI)	s	81	A	C	11	9947	53	977	63	e-37	460
Body	Estra										
mass	diol	rs71						0.0		1.39	
index	level	329				5026	0.3844	297	0.00203	991	461
(BMI)	s	08	A	G	12	3148	58	904	363	e-48	460
Body	Estra										
mass	diol	rs71						0.0		6.70	
index	level	495				3397	0.0769	277	0.00370	039	461
(BMI)	s	038	A	G	10	1383	36	998	969	e-14	460
Body	Estra							-0.			
mass	diol	rs72						014		6.00	
index	level	018				4077	0.3542	978	0.00207	067	461
(BMI)	s	95	A	G	16	23	91	1	999	e-13	460
Body	Estra										
mass	diol	rs72						0.0		1.79	
index	level	066				8287	0.3216	135	0.00211	999	461
(BMI)	s	08	G	C	16	2628	2	122	797	e-10	460
Body	Estra										
mass	diol	rs72						0.0		2.90	
index	level	180				6583	0.1973	189	0.00249	001	461
(BMI)	s	14	C	T	17	2016	05	524	216	e-14	460
Body	Estra										
mass	diol	rs72						0.0			
index	level	321				3125	0.5826	123	0.00200	8e-1	461
(BMI)	s	71	T	G	18	1221	3	404	821	0	460
Body	Estra										
mass	diol	rs72						0.0		2.99	
index	level	367				2161	0.4315	111	0.00200	999	461
(BMI)	s	2	T	C	12	561	1	273	698	e-08	460
Body	Estra										
mass	diol	rs72						0.0		6.69	
index	level	508				3393	0.2888	135	0.00218	993	461
(BMI)	s	33	T	C	19	7277	66	041	8	e-10	460
Body	Estra	rs72				4756	0.5960	0.0	0.00203	6.59	461
mass	diol	590	C	T	19	2509	62	218	628	933	460

index (BMI)	levels	70						69		e-27	
Body mass index (BMI)	Estradiol levels	rs72634826	A	G	1	1601052	0.259858	-0.021277	0.00228001	1e-20	461460
Body mass index (BMI)	Estradiol levels	rs72649373	C	T	4	80609966	0.143188	0.0178062	0.00287805	6.1e-10	461460
Body mass index (BMI)	Estradiol levels	rs72673947	G	A	8	118884379	0.107031	0.0218864	0.00321496	9.8992e-12	461460
Body mass index (BMI)	Estradiol levels	rs72892910	T	G	6	50816887	0.172232	0.0387798	0.00262053	1.50003e-49	461460
Body mass index (BMI)	Estradiol levels	rs72976986	A	G	19	4050424	0.190123	-0.0232257	0.00254712	7.59976e-20	461460
Body mass index (BMI)	Estradiol levels	rs73026725	A	C	19	31017686	0.153534	-0.022318	0.00275052	4.90004e-16	461460
Body mass index (BMI)	Estradiol levels	rs73052033	C	T	3	185828465	0.18493	-0.0303929	0.00254634	7.70016e-33	461460
Body mass index (BMI)	Estradiol levels	rs7306534	A	G	12	68107914	0.621712	-0.0112754	0.00205437	4.09996e-08	461460
Body mass index (BMI)	Estradiol levels	rs73124396	C	T	7	71579606	0.205144	-0.0154472	0.0024548	3.09999e-10	461460
Body mass index (BMI)	Estradiol levels	rs73142879	T	C	20	51195932	0.192297	-0.0266936	0.00252257	3.59998e-26	461460
Body mass	Estradiol	rs73193	G	A	12	10829438	0.243934	-0.017	0.00232002	2.19989	461460

index	level	736				1		716		e-14	
(BMI)	s							5			
Body	Estra							-0.			
mass	diol	rs73						022		1.69	
index	level	213				2848	0.1412	576	0.00283	981	461
(BMI)	s	484	T	A	4	9339	36	6	64	e-15	460
Body	Estra							-0.			
mass	diol	rs73						014		6.59	
index	level	314				9923	0.2852	370	0.00220	933	461
(BMI)	s	20	A	G	13	6471	76	2	081	e-11	460
Body	Estra										
mass	diol	rs73						0.0		1.10	
index	level	577				9220	0.5001	141	0.00198	002	461
(BMI)	s	54	G	A	9	7308	39	146	362	e-12	460
Body	Estra										
mass	diol	rs73						0.0		1.29	
index	level	601				1854	0.1145	177	0.00311	999	461
(BMI)	s	548	T	C	10	9889	27	354	639	e-08	460
Body	Estra										
mass	diol	rs73				2122		0.0			
index	level	985				9924	0.3072	136	0.00214	1.7e	461
(BMI)	s	439	C	A	2	9	99	616	059	-10	460
Body	Estra							-0.			
mass	diol	rs74						022		3.19	
index	level	428				8768	0.2140	816	0.00241	963	461
(BMI)	s	85	G	C	5	2877	49	3	251	e-21	460
Body	Estra										
mass	diol	rs74				1054		0.0		1.10	
index	level	524				6033	0.2819	176	0.00219	002	461
(BMI)	s	9	T	C	2	3	45	118	724	e-15	460
Body	Estra							-0.			
mass	diol	rs74				1147		019			
index	level	750				4446	0.0866	638	0.00352	2.5e	461
(BMI)	s	282	C	T	7	3	23	2	561	-08	460
Body	Estra										
mass	diol	rs74						0.0		2.29	
index	level	986				2888	0.3996	268	0.00201	985	461
(BMI)	s	65	G	A	16	3241	59	646	997	e-40	460
Body	Estra										
mass	diol	rs75				2103		0.0		2.59	
index	level	165				0133	0.3999	120	0.00201	998	461
(BMI)	s	54	T	C	1	1	41	066	565	e-09	460
Body	Estra	rs75				6643	0.5283	0.0	0.00198	1.69	461
mass	diol	192	A	G	1	4743	6	140	381	981	460

index	level	59						028		e-12	
(BMI)	s										
Body	Estra										
mass	diol	rs75						0.0		1.50	
index	level	463				4230	0.8865	219	0.00310	003	461
(BMI)	s	5	G	C	3	5131	82	925	93	e-12	460
Body	Estra							-0.			
mass	diol	rs75						018		8.90	
index	level	499				2614	0.2201	033	0.00241	02e-	461
(BMI)	s	503	T	C	6	5217	01	1	845	14	460
Body	Estra							-0.			
mass	diol	rs75						015		1.80	
index	level	714				6169	0.2605	891	0.00225	011	461
(BMI)	s	96	G	A	2	351	45	6	454	e-12	460
Body	Estra							-0.			
mass	diol	rs76				1143		021			
index	level	183				7193	0.0807	958	0.00364	1.7e	461
(BMI)	s	894	C	T	3	9	47	4	437	-09	460
Body	Estra										
mass	diol	rs76						0.0		2.19	
index	level	191				2511	0.5886	134	0.00201	989	461
(BMI)	s	39	A	T	3	0415	16	537	061	e-11	460
Body	Estra							-0.			
mass	diol	rs76				1951		016		1.20	
index	level	702				4829	0.2106	486	0.00243	005	461
(BMI)	s	514	G	C	1	6	23	2	278	e-11	460
Body	Estra							-0.			
mass	diol	rs76				1801		012		8.19	
index	level	838				6790	0.5572	246	0.00199	993	461
(BMI)	s	36	A	G	4	6	15	9	427	e-10	460
Body	Estra							-0.			
mass	diol	rs77				1535		015		1.39	
index	level	085				4346	0.5723	932	0.00199	991	461
(BMI)	s	84	G	A	5	6	44	1	49	e-15	460
Body	Estra							-0.			
mass	diol	rs77						013		1.29	
index	level	616				7035	0.2198	591	0.00239	999	461
(BMI)	s	73	A	T	6	7368	8	5	01	e-08	460
Body	Estra										
mass	diol	rs77				1533		0.0		9.09	
index	level	627				8022	0.2854	149	0.00218	913	461
(BMI)	s	94	G	A	6	8	08	077	606	e-12	460
Body	Estra	rs77				4801	0.3104	0.0	0.00215	3.29	461
mass	diol	74	A	C	17	163	67	149	169	989	460

index	level							88		e-12	
(BMI)	s										
Body	Estra										
mass	diol	rs77						0.0		1.40	
index	level	760				7374	0.2875	123	0.00218	001	461
(BMI)	s	21	A	G	6	2152	99	701	251	e-08	460
Body	Estra										
mass	diol	rs78				1374		0.0		1.79	
index	level	023				3592	0.2885	122	0.00218	999	461
(BMI)	s	42	G	T	7	5	66	748	145	e-08	460
Body	Estra										
mass	diol	rs78						0.0		1.80	
index	level	054				7812	0.5022	133	0.00198	011	461
(BMI)	s	41	T	C	7	1458	61	747	884	e-11	460
Body	Estra										
mass	diol	rs78						0.0		3.59	
index	level	086				2402	0.0398	318	0.00507	998	461
(BMI)	s	698	C	T	12	4639	38	357	61	e-10	460
Body	Estra										
mass	diol	rs78						0.0		1.99	
index	level	425				5339	0.8125	179	0.00254	986	461
(BMI)	s	7	C	T	18	7199	41	315	968	e-12	460
Body	Estra										
mass	diol	rs78						-0.		1.80	
index	level	605				8363	0.0540	032	0.00444	011	461
(BMI)	s	811	C	A	3	1491	5	737	673	e-13	460
Body	Estra										
mass	diol	rs78						0.0		2.29	
index	level	935				1675	0.6658	140	0.00210	985	461
(BMI)	s	71	T	G	10	0129	97	553	152	e-11	460
Body	Estra										
mass	diol	rs79				1189		0.0		3.29	
index	level	251				4159	0.3961	147	0.00202	989	461
(BMI)	s	00	A	G	11	6	17	25	21	e-13	460
Body	Estra										
mass	diol	rs79				1307		0.0		2.19	
index	level	447				9569	0.5097	157	0.00198	989	461
(BMI)	s	82	G	T	11	8	84	606	748	e-15	460
Body	Estra							-0.			
mass	diol	rs79						018		8.30	
index	level	471				6409	0.1634	278	0.00267	042	461
(BMI)	s	43	A	G	11	0422	83	2	522	e-12	460
Body	Estra	rs79				1049	0.0774	0.0	0.00369	1.5e	461
mass	diol	780	T	C	10	5249	28	236	591	-10	460

index	level	963				9		803			
(BMI)	s										
Body	Estra										
mass	diol	rs79						0.0		3.59	
index	level	966				9701	0.4493	145	0.00200	998	461
(BMI)	s	39	A	G	13	9090	54	559	245	e-13	460
Body	Estra										
mass	diol	rs80						0.0		3.79	
index	level	135				5286	0.0703	213	0.00388	997	461
(BMI)	s	274	T	A	17	277	24	39	113	e-08	460
Body	Estra										
mass	diol	rs80						0.0		6.70	
index	level	154				2593	0.6770	213	0.00211	039	461
(BMI)	s	00	A	C	14	0988	97	422	709	e-24	460
Body	Estra										
mass	diol	rs80						0.0			
index	level	203				7993	0.2204	251	0.00239	1e-2	461
(BMI)	s	65	A	T	14	7216	64	068	517	5	460
Body	Estra										
mass	diol	rs80						0.0			
index	level	241				3583	0.8482	156	0.00276	1.6e	461
(BMI)	s	37	T	A	15	7297	07	329	778	-08	460
Body	Estra							-0.			
mass	diol	rs80						014		1.69	
index	level	255				9527	0.6459	655	0.00207	981	461
(BMI)	s	16	G	T	15	1872	28	8	578	e-12	460
Body	Estra										
mass	diol	rs80						0.0		1.80	
index	level	766				1588	0.5615	140	0.00199	011	461
(BMI)	s	69	C	T	17	8448	61	681	601	e-12	460
Body	Estra										
mass	diol	rs80						0.0		3.89	
index	level	895				6922	0.3686	129	0.00207	996	461
(BMI)	s	14	A	T	18	4478	8	88	588	e-10	460
Body	Estra										
mass	diol	rs81						-0.		1.80	
index	level	128				1881	0.4003	020	0.00202	011	461
(BMI)	s	18	G	A	19	2785	29	696	712	e-24	460
Body	Estra										
mass	diol	rs81						-0.		2.39	
index	level	324				4028	0.3130	015	0.00219	994	461
(BMI)	s	91	A	G	21	8577	23	375	317	e-12	460
Body	Estra	rs81				1902	0.5631	-0.	0.00198	1e-1	461
mass	diol	516	C	T	1	9472	48	016	582	6	460

index	level	3				6		485			
(BMI)	s										
Body	Estra							-0.			
mass	diol	rs85						013		1.40	
index	level	204				1709	0.7585	120	0.00231	001	461
(BMI)	s	2	G	A	20	1233	75	7	309	e-08	460
Body	Estra							-0.			
mass	diol	rs86						023		1.20	
index	level	232				6965	0.4096	170	0.00201	005	461
(BMI)	s	0	T	C	16	1866	5	3	345	e-30	460
Body	Estra										
mass	diol	rs87						0.0		2.29	
index	level	962				4015	0.6132	241	0.00203	985	461
(BMI)	s	0	T	C	16	729	37	136	595	e-32	460
Body	Estra							-0.			
mass	diol	rs90						018		2.59	
index	level	989				4198	0.1348	410	0.00291	998	461
(BMI)	s	2	A	G	20	2698	17	5	255	e-10	460
Body	Estra							-0.			
mass	diol	rs92						014		1.29	
index	level	399				6780	0.7831	576	0.00240	999	461
(BMI)	s	4	G	A	4	2992	95	1	219	e-09	460
Body	Estra										
mass	diol	rs92						0.0		2.59	
index	level	676				3188	0.0607	261	0.00413	998	461
(BMI)	s	71	A	G	6	0480	33	49	587	e-10	460
Body	Estra										
mass	diol	rs92						-0.		8.60	
index	level	918				6407	0.5148	014	0.00199	003	461
(BMI)	s	22	T	C	5	6515	68	263	464	e-13	460
Body	Estra										
mass	diol	rs92						0.0			
index	level	942				8343	0.4765	147	0.00198	1e-1	461
(BMI)	s	60	A	G	6	3228	59	82	818	3	460
Body	Estra										
mass	diol	rs93						0.0		2.59	
index	level	492				4251	0.4106	111	0.00200	998	461
(BMI)	s	35	T	C	6	6718	35	809	924	e-08	460
Body	Estra							-0.			
mass	diol	rs93						016		3.19	
index	level	516				2694	0.5068	106	0.00197	963	461
(BMI)	s	6	A	G	2	9366	05	6	292	e-16	460
Body	Estra	rs94				9510	0.3389	-0.	0.00210	4.39	461
mass	diol	631	T	C	6	030	58	011	115	997	460

index (BMI)	levels	75						5		e-08	
Body mass index (BMI)	Estradiol levels	rs9478496	C	T	6	154333183	0.164225	0.0181836	0.00267493	1.10002e-11	461460
Body mass index (BMI)	Estradiol levels	rs9515446	G	A	13	112217108	0.447709	0.0151051	0.00199052	3.19963e-14	461460
Body mass index (BMI)	Estradiol levels	rs9522180	T	C	13	111970212	0.553392	-0.0141095	0.00199325	1.50003e-12	461460
Body mass index (BMI)	Estradiol levels	rs9571687	A	C	13	67472713	0.329363	-0.0135388	0.0021094	1.40001e-10	461460
Body mass index (BMI)	Estradiol levels	rs9638713	G	A	7	14645949	0.974777	-0.0361735	0.00635399	1.2e-08	461460
Body mass index (BMI)	Estradiol levels	rs9673839	G	A	16	76895693	0.490967	0.0130337	0.00198783	5.50047e-11	461460
Body mass index (BMI)	Estradiol levels	rs9674487	G	C	17	80191995	0.001338	0.158445	0.0286171	3.09999e-08	461460
Body mass index (BMI)	Estradiol levels	rs9830592	A	C	3	104631603	0.582421	0.0154849	0.00200164	1e-14	461460
Body mass index (BMI)	Estradiol levels	rs9839081	A	G	3	123051230	0.325232	-0.0110701	0.00214045	4.60002e-08	461460
Body mass index (BMI)	Estradiol levels	rs9843653	C	T	3	49920571	0.511652	0.0294509	0.00197538	2.90001e-50	461460
Body mass	Estradiol	rs98766	T	G	3	85806313	0.375389	-0.018	0.00204174	9.60064	461460

index (BMI)	levels	64						0478		e-19	
Body mass index (BMI)	Estradiol levels	rs9888533	T	C	13	107854612	0.538079	0.0120038	0.0020182	2.69998e-09	461460
Body mass index (BMI)	Estradiol levels	rs9926784	C	T	16	19941968	0.184576	-0.023818	0.00254677	8.60003e-21	461460
Body mass index (BMI)	Estradiol levels	rs9951619	G	T	18	56882326	0.767369	0.0144231	0.00235777	9.49992e-10	461460

Supplementary Table S3. Instruments for causal estimation from BMI to SHBG level.

Exposure	Outcome	SNP	Effect allele	Other allele	Chromosome	Genetic position	Effect allele frequency	Beta	Standard error of beta	Pvalue	Sample size
Body mass index (BMI)	Sex hormone-binding globulin levels	rs10063055	T	C	5	14090108	0.253365	0.0136775	0.00226991	1.7e-09	461460
Body mass index (BMI)	Sex hormone-binding globulin levels	rs10099330	G	A	8	14338694	0.45285	0.0124247	0.00198907	4.20001e-10	461460
Body mass index (BMI)	Sex hormone-binding globulin levels	rs10160769	C	G	11	76474827	0.21751	-0.0155862	0.00242199	1.2e-10	461460
Body mass index (BMI)	Sex hormone-binding globulin levels	rs10169594	C	T	2	41637688	0.363139	0.0121875	0.00205506	2.99999e-09	461460
Body mass index (BMI)	Sex hormone-binding globulin levels	rs10182416	G	A	2	10424292	0.512223	0.0130378	0.00197134	3.69999e-11	461460
Body mass index (BMI)	Sex hormone-binding globulin levels	rs10423928	A	T	19	46182304	0.194358	-0.0340136	0.00249869	3.40017e-42	461460
Body mass index (BMI)	Sex hormone-binding globulin levels	rs10505836	C	A	12	19288508	0.860012	0.0184851	0.00287086	1.2e-10	461460
Body mass	Sex hormone-b	rs1051	T	C	10	11865099	0.247013	0.017	0.00229886	2.1998	461460

index (BMI)	inding globulin levels	0025				6		5643		9e-14	
Body mass index (BMI)	Sex hormone-binding globulin levels	rs1064213	A	G	2	198950240	0.478377	0.0149301	0.00197196	3.69999e-14	461460
Body mass index (BMI)	Sex hormone-binding globulin levels	rs10742752	C	T	11	45438374	0.612239	0.0117979	0.00202961	6.1e-09	461460
Body mass index (BMI)	Sex hormone-binding globulin levels	rs10756714	G	A	9	15885041	0.444196	-0.0207862	0.00199408	1.9002e-25	461460
Body mass index (BMI)	Sex hormone-binding globulin levels	rs10756792	T	C	9	16726119	0.742901	-0.0190582	0.00227201	4.90004e-17	461460
Body mass index (BMI)	Sex hormone-binding globulin levels	rs10760277	T	C	9	126093999	0.385049	0.0138734	0.00203825	1e-11	461460
Body mass index (BMI)	Sex hormone-binding globulin levels	rs10780248	A	G	9	81370555	0.559454	-0.0121232	0.00199415	1.2e-09	461460
Body mass index (BMI)	Sex hormone-binding globulin levels	rs1078141	T	C	8	142619393	0.383867	0.0142239	0.00205862	4.90004e-12	461460
Body mass index (BMI)	Sex hormone-binding globulin levels	rs10799778	G	T	1	23313353	0.833718	-0.0182237	0.00264876	6.0067e-12	461460
Body	Sex	rs1	G	C	9	1185	0.3501	-0.	0.0020	1.4	461

mass index (BMI)	hormone-binding globulin levels	0809621				9607	43	0125305	7043	0001e-09	460
Body mass index (BMI)	Sex hormone-binding globulin levels	rs10824211	T	C	10	76363107	0.139491	0.0207688	0.00286631	4.30031e-13	461460
Body mass index (BMI)	Sex hormone-binding globulin levels	rs10832778	G	C	11	17394073	0.623038	0.0115628	0.00203985	1.40001e-08	461460
Body mass index (BMI)	Sex hormone-binding globulin levels	rs10927006	C	T	1	243557659	0.143704	-0.0168156	0.00281467	2.30001e-09	461460
Body mass index (BMI)	Sex hormone-binding globulin levels	rs10965698	T	C	9	23203619	0.369897	-0.0112741	0.002052	3.89996e-08	461460
Body mass index (BMI)	Sex hormone-binding globulin levels	rs10989067	A	G	9	103119634	0.31593	0.0169514	0.00212349	1.39991e-15	461460
Body mass index (BMI)	Sex hormone-binding globulin levels	rs11001963	T	C	10	78760959	0.581338	0.0116794	0.00201921	7.29995e-09	461460
Body mass index (BMI)	Sex hormone-binding globulin levels	rs11009685	T	C	10	34511990	0.244096	-0.0130834	0.00230766	1.40001e-08	461460
Body mass index (BMI)	Sex hormone-binding globulin levels	rs11012732	G	A	10	21830104	0.331683	0.0216425	0.00210126	7.10068e-25	461460

Body mass index (BMI)	Sex hormone-binding globulin levels	rs11079849	T	C	17	47090785	0.328531	-0.020093	0.00211163	1.80011e-21	461460
Body mass index (BMI)	Sex hormone-binding globulin levels	rs11099020	T	C	4	130724902	0.640609	-0.0142038	0.00206202	5.60015e-12	461460
Body mass index (BMI)	Sex hormone-binding globulin levels	rs11115160	A	G	12	82424100	0.237797	-0.0130768	0.00233641	2.19999e-08	461460
Body mass index (BMI)	Sex hormone-binding globulin levels	rs11122450	G	T	1	230301811	0.611739	-0.0116316	0.00202425	9.09997e-09	461460
Body mass index (BMI)	Sex hormone-binding globulin levels	rs11134679	G	A	5	170623391	0.684753	0.0182459	0.00213286	1.20005e-17	461460
Body mass index (BMI)	Sex hormone-binding globulin levels	rs11150745	G	A	17	78757626	0.317711	-0.0211611	0.00212961	2.90001e-23	461460
Body mass index (BMI)	Sex hormone-binding globulin levels	rs111598585	T	C	4	171635471	0.208671	-0.0142245	0.00243798	5.39995e-09	461460
Body mass index (BMI)	Sex hormone-binding globulin levels	rs11165643	T	C	1	96924097	0.590103	0.0193319	0.00200337	4.90004e-22	461460
Body mass index (BMI)	Sex hormone-binding globulin	rs111689389	C	G	5	27175962	0.282614	-0.013670	0.00219522	4.70002e-10	461460

	levels							6			
Body mass index (BMI)	Sex hormone-binding globulin levels	rs11218510	A	G	11	12192258 7	0.40047	-0.0144616	0.00202121	8.4004e-13	461460
Body mass index (BMI)	Sex hormone-binding globulin levels	rs113079574	T	C	4	14735408 9	0.19279	-0.0155896	0.00251598	5.80003e-10	461460
Body mass index (BMI)	Sex hormone-binding globulin levels	rs113624107	A	G	14	88326386	0.22586	0.0150461	0.00236876	2.1e-10	461460
Body mass index (BMI)	Sex hormone-binding globulin levels	rs11525873	C	T	7	13881719 3	0.097701	-0.0239781	0.00333603	6.5993e-13	461460
Body mass index (BMI)	Sex hormone-binding globulin levels	rs11607476	C	A	11	11503706 1	0.486574	0.0157122	0.00199179	3.10027e-15	461460
Body mass index (BMI)	Sex hormone-binding globulin levels	rs11610621	A	T	12	12167113 3	0.148174	0.0164869	0.00278318	3.09999e-09	461460
Body mass index (BMI)	Sex hormone-binding globulin levels	rs11630647	A	G	15	99240947	0.251739	-0.0112674	0.00228082	2.69998e-08	461460
Body mass index (BMI)	Sex hormone-binding globulin levels	rs116374395	A	G	5	50723410	0.035444	0.0318817	0.00536236	2.80001e-09	461460
Body mass index	Sex hormone-binding	rs1164209	C	T	16	81730582	0.373536	0.01141	0.00205925	2.90001e-	461460

(BMI)	globulin levels	0						84		08	
Body mass index (BMI)	Sex hormone-binding globulin levels	rs11656076	A	G	17	31464270	0.224774	-0.0154285	0.00237054	7.59976e-11	461460
Body mass index (BMI)	Sex hormone-binding globulin levels	rs1167311	A	G	1	49996959	0.681309	-0.0192649	0.00213109	1.59993e-19	461460
Body mass index (BMI)	Sex hormone-binding globulin levels	rs11675464	G	A	2	204053742	0.562988	0.0119751	0.00198558	1.6e-09	461460
Body mass index (BMI)	Sex hormone-binding globulin levels	rs11691869	A	C	2	100805996	0.362038	-0.0193158	0.00205562	5.60015e-21	461460
Body mass index (BMI)	Sex hormone-binding globulin levels	rs11699828	A	G	20	62157198	0.035832	-0.0335418	0.00582677	8.60003e-09	461460
Body mass index (BMI)	Sex hormone-binding globulin levels	rs11709402	G	A	3	131551027	0.278732	0.0228192	0.00220796	4.90004e-25	461460
Body mass index (BMI)	Sex hormone-binding globulin levels	rs117118217	C	G	10	131783328	0.017711	0.0448186	0.00788004	1.29999e-08	461460
Body mass index (BMI)	Sex hormone-binding globulin levels	rs11757278	C	T	6	13180454	0.303879	-0.0146269	0.00214834	9.8992e-12	461460
Body mass	Sex hormone-b	rs1177	G	A	8	87762607	0.163123	0.015	0.0026874	4.2000	461460

index (BMI)	inding globulin levels	8219						796		1e-09	
Body mass index (BMI)	Sex hormone-binding globulin levels	rs11919665	T	A	3	48085349	0.679775	-0.0127854	0.00211292	1.40001e-09	461460
Body mass index (BMI)	Sex hormone-binding globulin levels	rs12001437	C	T	9	34074476	0.367678	0.0121836	0.00205069	2.80001e-09	461460
Body mass index (BMI)	Sex hormone-binding globulin levels	rs12072739	G	A	1	98315893	0.224478	0.0157008	0.00236691	3.29989e-11	461460
Body mass index (BMI)	Sex hormone-binding globulin levels	rs12088284	T	C	1	80798635	0.300838	0.0139317	0.00214854	8.9002e-11	461460
Body mass index (BMI)	Sex hormone-binding globulin levels	rs12089815	A	G	1	91189933	0.54861	-0.0123121	0.00198606	5.69994e-10	461460
Body mass index (BMI)	Sex hormone-binding globulin levels	rs12140153	T	G	1	62579891	0.094252	-0.0303075	0.0034589	1.20005e-21	461460
Body mass index (BMI)	Sex hormone-binding globulin levels	rs12149660	A	G	16	70309237	0.114956	-0.0227338	0.00311586	2.99985e-13	461460
Body mass index (BMI)	Sex hormone-binding globulin levels	rs12259464	A	G	10	53680099	0.48447	0.0130895	0.00198686	4.49987e-11	461460
Body	Sex	rs1	T	C	11	1169	0.0565	0.0	0.0042	6.8	461

mass index (BMI)	hormone-binding globulin levels	2273545				11012	05	248393	8551	0002e-09	460
Body mass index (BMI)	Sex hormone-binding globulin levels	rs1229984	C	T	4	100239319	0.972775	0.037357	0.00599385	4.60002e-10	461460
Body mass index (BMI)	Sex hormone-binding globulin levels	rs12364470	G	T	11	134601012	0.164556	0.0192705	0.0026658	4.90004e-13	461460
Body mass index (BMI)	Sex hormone-binding globulin levels	rs12440603	T	C	15	46585722	0.433693	0.0139285	0.0020011	3.40017e-12	461460
Body mass index (BMI)	Sex hormone-binding globulin levels	rs12459368	G	A	19	18459377	0.268203	-0.0170141	0.00223235	2.49977e-14	461460
Body mass index (BMI)	Sex hormone-binding globulin levels	rs12462975	A	G	19	30272202	0.329692	0.0195822	0.00212071	2.60016e-20	461460
Body mass index (BMI)	Sex hormone-binding globulin levels	rs12541408	C	T	8	95585807	0.31742	-0.0143261	0.00212723	1.59993e-11	461460
Body mass index (BMI)	Sex hormone-binding globulin levels	rs1266874	G	A	6	51779638	0.349619	0.0140966	0.0020712	1e-11	461460
Body mass index (BMI)	Sex hormone-binding globulin levels	rs12681792	A	C	8	62054463	0.1926	0.0148693	0.00251693	3.50002e-09	461460

	Sex										
Body	hormone-b	rs1						0.0		1.2	
mass	inding	269				1612		13		999	
index	globulin	259				6591	0.3718	08	0.0020	9e-	461
(BMI)	levels	6	T	C	2	0	5	72	3738	10	460
	Sex							-0.			
Body	hormone-b	rs1						01		3.5	
mass	inding	269				1563		52		000	
index	globulin	603				0475	0.1494	80	0.0027	2e-	461
(BMI)	levels	9	G	A	3	0	05	2	705	08	460
	Sex										
Body	hormone-b							0.0		6.2	
mass	inding	rs1						14		001	
index	globulin	286				9145	0.7037	91	0.0021	2e-	461
(BMI)	levels	058	A	T	14	8523	47	26	6922	12	460
	Sex										
Body	hormone-b	rs1						0.0		7.6	
mass	inding	288				1011		22		999	
index	globulin	162				4641	0.0826	07	0.0035	9e-	461
(BMI)	levels	9	G	A	14	3	5	48	8929	10	460
	Sex										
Body	hormone-b	rs1						0.0		3.7	
mass	inding	292						20		999	
index	globulin	198				7231	0.0779	33	0.0036	7e-	461
(BMI)	levels	6	G	A	16	2727	84	27	983	08	460
	Sex										
Body	hormone-b							-0.		3.9	
mass	inding	rs1				1370		01		003	
index	globulin	296				8319	0.5590	88	0.0019	2e-	461
(BMI)	levels	328	C	A	4	3	32	62	99	21	460
	Sex										
Body	hormone-b	rs1						0.0		2.2	
mass	inding	297						15		998	
index	globulin	445				1866	0.5431	24	0.0019	5e-	461
(BMI)	levels	8	T	C	19	115	75	66	9717	14	460
	Sex							-0.			
Body	hormone-b	rs1						01			
mass	inding	301						36			
index	globulin	207				3544	0.2283	50	0.0023	6.1e	461
(BMI)	levels	0	A	G	2	7243	88	1	4795	-09	460
Body	Sex	rs1						0.0		3.2	
mass	hormone-b	303				1335		12		999	
index	inding	331				2360	0.2527	61	0.0022	7e-	461
(BMI)	globulin	0	A	G	2	5	6	1	826	08	460

	levels										
Body mass index (BMI)	Sex hormone-binding globulin levels	rs13097918	A	T	3	35676330	0.212315	0.0145561	0.0024161	1.7e-09	461460
Body mass index (BMI)	Sex hormone-binding globulin levels	rs13107325	T	C	4	103188709	0.07492	0.0475799	0.00375479	8.49963e-37	461460
Body mass index (BMI)	Sex hormone-binding globulin levels	rs13176429	C	T	5	43152216	0.687609	0.0141555	0.00213122	3.10027e-11	461460
Body mass index (BMI)	Sex hormone-binding globulin levels	rs1320251	T	C	17	21264396	0.454791	-0.0180321	0.00199407	1.50003e-19	461460
Body mass index (BMI)	Sex hormone-binding globulin levels	rs13218383	G	C	6	120173501	0.335132	-0.0144025	0.00209236	5.79963e-12	461460
Body mass index (BMI)	Sex hormone-binding globulin levels	rs1322842	G	A	6	20488897	0.609274	-0.0131292	0.00203539	1.09999e-10	461460
Body mass index (BMI)	Sex hormone-binding globulin levels	rs13248187	C	T	8	14336834	0.268557	0.0157664	0.00224155	1.99986e-12	461460
Body mass index (BMI)	Sex hormone-binding globulin levels	rs1327259	G	A	6	51177811	0.387722	-0.0148532	0.00203363	2.80027e-13	461460
Body mass index	Sex hormone-binding	rs1329172	A	G	9	80510077	0.570683	0.01103	0.00199876	3.40001e-	461460

(BMI)	globulin levels	3						18		08	
Body mass index (BMI)	Sex hormone-binding globulin levels	rs1330199	T	G	9	27760946	0.483137	-0.0117509	0.00198642	3.29997e-09	461460
Body mass index (BMI)	Sex hormone-binding globulin levels	rs13420048	A	C	2	50751414	0.365037	-0.0154817	0.00205272	4.60045e-14	461460
Body mass index (BMI)	Sex hormone-binding globulin levels	rs13427822	G	A	2	213414265	0.271198	-0.0181515	0.00224142	5.60015e-16	461460
Body mass index (BMI)	Sex hormone-binding globulin levels	rs1346841	A	G	4	65651730	0.405041	-0.0130 56	0.0020172	9.70063e-11	461460
Body mass index (BMI)	Sex hormone-binding globulin levels	rs1360201	T	C	9	73796450	0.481546	0.0130075	0.00197747	4.79954e-11	461460
Body mass index (BMI)	Sex hormone-binding globulin levels	rs13642	T	A	11	30432220	0.360654	-0.0161363	0.00205695	4.30031e-15	461460
Body mass index (BMI)	Sex hormone-binding globulin levels	rs140159717	T	C	15	73765586	0.082292	-0.0246882	0.00370967	2.80027e-11	461460
Body mass index (BMI)	Sex hormone-binding globulin levels	rs1438945	A	T	5	152510937	0.715119	-0.0133824	0.00219791	1.09999e-09	461460
Body mass	Sex hormone-b	rs1441	A	G	13	79580919	0.593681	0.017	0.00205854	3.4001	461460

index (BMI)	inding globulin levels	264						9033		7e-18	
Body mass index (BMI)	Sex hormone-binding globulin levels	rs1451963	T	G	14	41350367	0.082266	0.022201	0.00360585	7.39997e-10	461460
Body mass index (BMI)	Sex hormone-binding globulin levels	rs1458156	T	C	12	41887940	0.488431	0.0140775	0.00197951	1.20005e-12	461460
Body mass index (BMI)	Sex hormone-binding globulin levels	rs145981104	G	A	8	74714869	0.063711	0.0227711	0.00404552	2e-08	461460
Body mass index (BMI)	Sex hormone-binding globulin levels	rs146569428	A	G	11	2199686	0.200749	0.0139533	0.00248571	2e-08	461460
Body mass index (BMI)	Sex hormone-binding globulin levels	rs1471093	A	G	3	108031094	0.616663	0.0134619	0.00203996	4.10015e-11	461460
Body mass index (BMI)	Sex hormone-binding globulin levels	rs147568678	C	T	10	93061851	0.23807	-0.0133327	0.00233034	1.09999e-08	461460
Body mass index (BMI)	Sex hormone-binding globulin levels	rs1477290	C	T	5	87988934	0.136947	0.0337772	0.00289815	2.19989e-31	461460
Body mass index (BMI)	Sex hormone-binding globulin levels	rs147730268	T	G	12	123024476	0.087243	-0.0350799	0.00358357	1.29987e-22	461460
Body	Sex	rs1	C	T	5	6302	0.4800	0.0	0.0019	5.9	461

mass index (BMI)	hormone-binding globulin levels	503526				0706	77	154308	7681	0065e-15	460
Body mass index (BMI)	Sex hormone-binding globulin levels	rs156201	C	G	6	104847441	0.753455	0.0131899	0.00228932	8.30004e-09	461460
Body mass index (BMI)	Sex hormone-binding globulin levels	rs156914	A	G	1	16848652	0.491786	0.0111552	0.00197308	1.6e-08	461460
Body mass index (BMI)	Sex hormone-binding globulin levels	rs1582931	A	G	5	122657199	0.473242	-0.0133419	0.0019956	2.29985e-11	461460
Body mass index (BMI)	Sex hormone-binding globulin levels	rs1608113	T	A	3	157815217	0.364998	-0.0117766	0.00205086	9.29994e-09	461460
Body mass index (BMI)	Sex hormone-binding globulin levels	rs1609010	G	A	8	77227464	0.565728	0.0209773	0.0019963	7.89951e-26	461460
Body mass index (BMI)	Sex hormone-binding globulin levels	rs16916303	G	A	9	30823761	0.119736	-0.0192369	0.00307931	4.20001e-10	461460
Body mass index (BMI)	Sex hormone-binding globulin levels	rs17056301	C	T	5	158271680	0.256425	0.0135831	0.00222697	2.19999e-09	461460
Body mass index (BMI)	Sex hormone-binding globulin levels	rs17132130	C	G	7	2108036	0.221467	-0.0178418	0.00238564	7.50067e-14	461460

Body mass index (BMI)	Sex hormone-binding globulin levels	rs17149254	C	T	7	76634463	0.804854	-0.0213507	0.00255804	7.00003e-17	461460
Body mass index (BMI)	Sex hormone-binding globulin levels	rs17289010	G	A	4	140774684	0.32787	-0.0134661	0.00210438	1.6e-10	461460
Body mass index (BMI)	Sex hormone-binding globulin levels	rs17399739	G	A	10	87490850	0.068893	0.0270713	0.00391006	4.40048e-12	461460
Body mass index (BMI)	Sex hormone-binding globulin levels	rs17446299	G	C	13	40762556	0.165544	0.0153235	0.00266515	8.9e-09	461460
Body mass index (BMI)	Sex hormone-binding globulin levels	rs17544384	C	T	1	115295160	0.210864	0.0140931	0.00241434	5.30005e-09	461460
Body mass index (BMI)	Sex hormone-binding globulin levels	rs17668356	G	C	3	61208619	0.146029	-0.0230543	0.00279318	1.50003e-16	461460
Body mass index (BMI)	Sex hormone-binding globulin levels	rs17770336	T	C	9	28414625	0.322437	0.0242931	0.00211161	1.29987e-30	461460
Body mass index (BMI)	Sex hormone-binding globulin levels	rs1778830	A	G	1	156489974	0.362165	0.0140775	0.00205596	7.50067e-12	461460
Body mass index (BMI)	Sex hormone-binding globulin	rs1788808	G	A	18	21090023	0.49478	-0.020404	0.00198255	7.70016e-25	461460

	levels										
Body mass index (BMI)	Sex hormone-binding globulin levels	rs1793636	C	G	11	131934926	0.309144	-0.0133343	0.0021408	4.70002e-10	461460
Body mass index (BMI)	Sex hormone-binding globulin levels	rs1805123	G	T	7	150645534	0.245301	-0.0167569	0.00229711	2.99985e-13	461460
Body mass index (BMI)	Sex hormone-binding globulin levels	rs1834144	A	C	18	40744790	0.373192	-0.0140112	0.00205213	8.60003e-12	461460
Body mass index (BMI)	Sex hormone-binding globulin levels	rs1861410	T	C	2	58933591	0.555423	-0.0212558	0.00198918	1.20005e-26	461460
Body mass index (BMI)	Sex hormone-binding globulin levels	rs1884897	G	A	20	6612832	0.627378	0.0200001	0.00205634	2.29985e-22	461460
Body mass index (BMI)	Sex hormone-binding globulin levels	rs1919243	C	T	5	88778861	0.487406	0.0116759	0.00200175	5.49997e-09	461460
Body mass index (BMI)	Sex hormone-binding globulin levels	rs1967772	A	G	13	28036062	0.285117	-0.0170434	0.00220377	1e-14	461460
Body mass index (BMI)	Sex hormone-binding globulin levels	rs2035936	T	G	3	141298124	0.055892	0.0370863	0.00435778	1.69981e-17	461460
Body mass index	Sex hormone-binding	rs2075466	C	G	16	4872970	0.267182	0.01329	0.00223627	2.80001e-	461460

(BMI)	globulin levels							03		09	
Body mass index (BMI)	Sex hormone-binding globulin levels	rs2102278	G	A	4	52818664	0.322486	0.0118583	0.0021139	2e-08	461460
Body mass index (BMI)	Sex hormone-binding globulin levels	rs2133561	T	A	5	139086651	0.611084	-0.014097	0.00204744	5.79963e-12	461460
Body mass index (BMI)	Sex hormone-binding globulin levels	rs213518	C	T	7	26941065	0.145608	0.0157894	0.00280491	1.79999e-08	461460
Body mass index (BMI)	Sex hormone-binding globulin levels	rs2153740	G	A	20	2126089	0.479914	-0.0112551	0.00199313	1.6e-08	461460
Body mass index (BMI)	Sex hormone-binding globulin levels	rs215634	G	A	7	32369148	0.611879	-0.0155223	0.00203492	2.39994e-14	461460
Body mass index (BMI)	Sex hormone-binding globulin levels	rs2172131	C	T	10	133978962	0.578723	-0.0149382	0.00200386	8.99912e-14	461460
Body mass index (BMI)	Sex hormone-binding globulin levels	rs217672	C	A	14	62361021	0.271741	0.0170155	0.00223046	2.39994e-14	461460
Body mass index (BMI)	Sex hormone-binding globulin levels	rs2192158	G	A	4	55505360	0.553308	-0.015012	0.00198302	3.69999e-14	461460
Body mass	Sex hormone-b	rs2216	A	C	2	18159907	0.661966	0.016	0.00208587	5.1999	461460

index (BMI)	inding globulin levels	931				0		9109		6e-16	
Body mass index (BMI)	Sex hormone-binding globulin levels	rs2234458	T	C	11	65639374	0.639519	-0.0203841	0.00205579	3.59998e-23	461460
Body mass index (BMI)	Sex hormone-binding globulin levels	rs2248551	A	G	6	131924689	0.164522	0.0146523	0.00266497	3.79997e-08	461460
Body mass index (BMI)	Sex hormone-binding globulin levels	rs2253310	G	C	6	108885932	0.626099	0.0173213	0.00204115	2.09991e-17	461460
Body mass index (BMI)	Sex hormone-binding globulin levels	rs2271189	A	G	12	56494991	0.40272	-0.0163086	0.00201869	6.4998e-16	461460
Body mass index (BMI)	Sex hormone-binding globulin levels	rs2289379	T	C	7	44804225	0.395648	-0.0152765	0.00202952	5.19996e-14	461460
Body mass index (BMI)	Sex hormone-binding globulin levels	rs2307111	C	T	5	75003678	0.395025	-0.0280042	0.00202203	1.29987e-43	461460
Body mass index (BMI)	Sex hormone-binding globulin levels	rs2342892	G	T	16	24540806	0.516212	-0.0126972	0.00197715	1.29999e-10	461460
Body mass index (BMI)	Sex hormone-binding globulin levels	rs2381404	C	T	2	144035442	0.243837	0.0139664	0.00229974	1.29999e-09	461460
Body	Sex	rs2	A	G	14	3325	0.1306	0.0	0.0029	3.8	461

mass index (BMI)	hormone-binding globulin levels	383377				7914	87	161287	3564	9996e-08	460
Body mass index (BMI)	Sex hormone-binding globulin levels	rs2398861	G	A	9	96430747	0.259169	0.0179932	0.00226733	2.09991e-15	461460
Body mass index (BMI)	Sex hormone-binding globulin levels	rs2425816	A	G	20	44895075	0.415111	0.0122284	0.00201106	1.2e-09	461460
Body mass index (BMI)	Sex hormone-binding globulin levels	rs2433733	A	G	2	230816703	0.677659	-0.0171805	0.00210907	3.80014e-16	461460
Body mass index (BMI)	Sex hormone-binding globulin levels	rs2439823	G	A	10	99778226	0.545612	0.0191998	0.00199108	5.30029e-22	461460
Body mass index (BMI)	Sex hormone-binding globulin levels	rs2482356	C	T	9	94178371	0.429018	-0.0113511	0.00199506	1.29999e-08	461460
Body mass index (BMI)	Sex hormone-binding globulin levels	rs2512892	C	T	11	131451862	0.566085	0.0129468	0.00199819	9.20026e-11	461460
Body mass index (BMI)	Sex hormone-binding globulin levels	rs252761	T	G	5	77380723	0.587966	-0.0115046	0.00201769	1.2e-08	461460
Body mass index (BMI)	Sex hormone-binding globulin levels	rs2568958	A	G	1	72765116	0.603656	0.0222928	0.00201204	1.59993e-28	461460

Body mass index (BMI)	Sex hormone-binding globulin levels	rs2569993	C	T	3	12926096	0.320327	0.01267	0.00212202	2.39999e-09	461460
Body mass index (BMI)	Sex hormone-binding globulin levels	rs2606228	C	A	3	183537759	0.646368	-0.0138791	0.00208379	2.70023e-11	461460
Body mass index (BMI)	Sex hormone-binding globulin levels	rs2616143	A	G	8	20632022	0.319862	-0.0138738	0.00212547	6.70039e-11	461460
Body mass index (BMI)	Sex hormone-binding globulin levels	rs2618039	T	A	1	112324111	0.381481	0.0143963	0.0020317	1.39991e-12	461460
Body mass index (BMI)	Sex hormone-binding globulin levels	rs2678204	G	T	1	201800511	0.340162	0.024161	0.00208158	3.80014e-31	461460
Body mass index (BMI)	Sex hormone-binding globulin levels	rs2725371	G	A	8	30854033	0.696115	-0.0160301	0.00215845	1.10002e-13	461460
Body mass index (BMI)	Sex hormone-binding globulin levels	rs28350	G	A	3	42418446	0.820684	-0.0180335	0.00258234	2.90001e-12	461460
Body mass index (BMI)	Sex hormone-binding globulin levels	rs28366156	C	T	6	31671498	0.130595	-0.0264826	0.00292978	1.59993e-19	461460
Body mass index (BMI)	Sex hormone-binding globulin	rs2837996	C	T	21	42626706	0.651408	0.0126647	0.00207859	1.09999e-09	461460

	levels										
Body mass index (BMI)	Sex hormone-binding globulin levels	rs28404639	T	C	5	80874229	0.366041	-0.0117129	0.00205533	1.2e-08	461460
Body mass index (BMI)	Sex hormone-binding globulin levels	rs28568418	A	G	15	53462969	0.107636	-0.0182825	0.00320812	1.2e-08	461460
Body mass index (BMI)	Sex hormone-binding globulin levels	rs2861685	C	T	2	67837553	0.411984	-0.0171274	0.00199763	1e-17	461460
Body mass index (BMI)	Sex hormone-binding globulin levels	rs28670671	C	T	9	140363045	0.286024	-0.0124638	0.00226426	3.69999e-08	461460
Body mass index (BMI)	Sex hormone-binding globulin levels	rs2870111	T	C	15	79403585	0.412066	-0.0157151	0.00201912	7.10068e-15	461460
Body mass index (BMI)	Sex hormone-binding globulin levels	rs2899644	T	C	15	59470366	0.229995	0.0149659	0.0023609	2.30001e-10	461460
Body mass index (BMI)	Sex hormone-binding globulin levels	rs2962334	T	G	5	86879056	0.020065	0.0432552	0.00703547	7.79992e-10	461460
Body mass index (BMI)	Sex hormone-binding globulin levels	rs317656	A	T	12	69681101	0.724452	-0.0144763	0.00221303	6.09958e-11	461460
Body mass index	Sex hormone-binding	rs32421	T	A	5	167362416	0.224315	0.01323	0.00237757	2.59998e-	461460

(BMI)	globulin levels							15		08	
Body mass index (BMI)	Sex hormone-binding globulin levels	rs329651	T	G	11	133767622	0.803977	0.0157218	0.00250202	3.29997e-10	461460
Body mass index (BMI)	Sex hormone-binding globulin levels	rs34045288	T	C	6	40369081	0.334435	0.0234784	0.00209371	3.50026e-29	461460
Body mass index (BMI)	Sex hormone-binding globulin levels	rs34153025	C	T	15	41339697	0.022158	-0.0389151	0.00678155	9.59997e-09	461460
Body mass index (BMI)	Sex hormone-binding globulin levels	rs34234296	A	G	2	175166636	0.392437	-0.0149477	0.00204032	2.39994e-13	461460
Body mass index (BMI)	Sex hormone-binding globulin levels	rs34481751	A	C	20	47501038	0.165329	-0.0185029	0.00270404	7.8001e-12	461460
Body mass index (BMI)	Sex hormone-binding globulin levels	rs34517439	A	C	1	78450517	0.121787	0.0388848	0.00304983	3.59998e-37	461460
Body mass index (BMI)	Sex hormone-binding globulin levels	rs34696181	C	T	7	93096635	0.476084	0.0114345	0.00198283	8.10009e-09	461460
Body mass index (BMI)	Sex hormone-binding globulin levels	rs34811474	A	G	4	25408838	0.230756	-0.0285293	0.00234269	4.10015e-34	461460
Body mass	Sex hormone-b	rs3490	A	G	11	84776849	0.500388	-0.01	0.00198231	1.9002	461460

index	inding	71						33		e-1	
(BMI)	globulin							06		1	
	levels							2			
	Sex							-0.			
Body	hormone-b	rs3						01		5.3	
mass	inding	515						30		000	
index	globulin	432				2486	0.2740	42	0.0022	5e-	461
(BMI)	levels	6	G	A	16	2414	44	7	3442	09	460
	Sex										
Body	hormone-b	rs3						0.0		9.2	
mass	inding	536						21		002	
index	globulin	444				7427	0.1097	70	0.0031	6e-	461
(BMI)	levels	9	T	C	15	8126	39	69	8335	12	460
	Sex										
Body	hormone-b							0.0		3.2	
mass	inding	rs3				1540		15		998	
index	globulin	557				3495	0.4075	27	0.0020	9e-	461
(BMI)	levels	77	C	G	3	0	37	15	1314	14	460
	Sex							-0.			
Body	hormone-b	rs3						01		9.2	
mass	inding	569						64		002	
index	globulin	758				4729	0.5081	67	0.0019	6e-	461
(BMI)	levels	7	A	G	14	8505	21	7	8067	17	460
	Sex										
Body	hormone-b	rs3						0.0		6.2	
mass	inding	569						23		001	
index	globulin	769				5235	0.0893	03	0.0035	2e-	461
(BMI)	levels	1	G	C	15	3498	46	05	2223	11	460
	Sex							-0.			
Body	hormone-b	rs3						01		9.0	
mass	inding	580						71		991	
index	globulin	900				4701	0.3632	00	0.0020	3e-	461
(BMI)	levels	7	A	G	2	9521	05	9	5627	17	460
	Sex							-0.			
Body	hormone-b	rs3						01		1.1	
mass	inding	595						96		000	
index	globulin	754				7344	0.5743	42	0.0020	2e-	461
(BMI)	levels	4	T	G	8	0371	16	9	0438	22	460
	Sex										
Body	hormone-b	rs3						-0.		2.1	
mass	inding	600				1630		02		998	
index	globulin	763				0933	0.1376	10	0.0028	9e-	461
(BMI)	levels	5	A	G	6	5	98	45	6788	13	460
Body	Sex	rs3	T	C	8	3832	0.3987	0.0	0.0020	2e-	461

mass index (BMI)	hormone-binding globulin levels	6061954				9650	47	128445	1928	10	460
Body mass index (BMI)	Sex hormone-binding globulin levels	rs3764625	G	T	19	49649051	0.587594	-0.0117714	0.00201523	5.19996e-09	461460
Body mass index (BMI)	Sex hormone-binding globulin levels	rs3784710	C	T	15	68072458	0.226602	-0.0297067	0.00236042	2.49977e-36	461460
Body mass index (BMI)	Sex hormone-binding globulin levels	rs3803286	G	A	14	103246470	0.666815	-0.0186417	0.00209828	6.4003e-19	461460
Body mass index (BMI)	Sex hormone-binding globulin levels	rs3807566	T	G	7	50564204	0.43832	-0.0120727	0.00199583	1.5e-09	461460
Body mass index (BMI)	Sex hormone-binding globulin levels	rs3814883	T	C	16	29994922	0.482402	0.0240107	0.00198424	1e-33	461460
Body mass index (BMI)	Sex hormone-binding globulin levels	rs3845344	T	C	1	75001480	0.391115	0.0163609	0.00201927	5.40008e-16	461460
Body mass index (BMI)	Sex hormone-binding globulin levels	rs3851998	G	C	3	131876605	0.743064	-0.0136071	0.00226874	2e-09	461460
Body mass index (BMI)	Sex hormone-binding globulin levels	rs3866805	A	C	1	6657424	0.355683	0.0117835	0.00206546	1.2e-08	461460

Body mass index (BMI)	Sex hormone-binding globulin levels	rs3897102	T	C	12	123492112	0.411157	0.0120838	0.00202716	2.5e-09	461460
Body mass index (BMI)	Sex hormone-binding globulin levels	rs3901286	A	C	7	99107727	0.15244	-0.0225553	0.00275602	2.70023e-16	461460
Body mass index (BMI)	Sex hormone-binding globulin levels	rs3902951	G	T	14	69789755	0.237151	0.0141019	0.00235267	2e-09	461460
Body mass index (BMI)	Sex hormone-binding globulin levels	rs3935190	A	G	17	79084367	0.536784	-0.0144864	0.00199701	4.00037e-13	461460
Body mass index (BMI)	Sex hormone-binding globulin levels	rs394608	C	T	21	46581798	0.537697	0.0186353	0.00199484	9.49948e-21	461460
Body mass index (BMI)	Sex hormone-binding globulin levels	rs40071	C	T	5	107496102	0.179536	-0.0261666	0.00258163	3.80014e-24	461460
Body mass index (BMI)	Sex hormone-binding globulin levels	rs4017425	T	C	3	44028764	0.470208	-0.0125817	0.00198035	2.1e-10	461460
Body mass index (BMI)	Sex hormone-binding globulin levels	rs4055791	T	C	13	59266053	0.416804	-0.0178082	0.00200801	7.39946e-19	461460
Body mass index (BMI)	Sex hormone-binding globulin	rs406388	G	C	22	18226997	0.177266	0.0159973	0.00260181	8.30004e-10	461460

	levels										
Body mass index (BMI)	Sex hormone-binding globulin levels	rs41279738	G	T	1	110082551	0.025988	0.0684263	0.0062225	4.00037e-28	461460
Body mass index (BMI)	Sex hormone-binding globulin levels	rs4148155	G	A	4	89054667	0.113268	-0.0229681	0.00310659	1.39991e-13	461460
Body mass index (BMI)	Sex hormone-binding globulin levels	rs4261944	G	T	4	31003636	0.364935	0.0138553	0.00205652	1.59993e-11	461460
Body mass index (BMI)	Sex hormone-binding globulin levels	rs4267103	C	T	12	60966740	0.186347	0.0153916	0.00254312	1.40001e-09	461460
Body mass index (BMI)	Sex hormone-binding globulin levels	rs4284600	C	T	15	31843528	0.467122	0.0119673	0.00199506	2e-09	461460
Body mass index (BMI)	Sex hormone-binding globulin levels	rs429343	G	A	2	147903382	0.576573	-0.0173795	0.00199729	3.29989e-18	461460
Body mass index (BMI)	Sex hormone-binding globulin levels	rs429358	C	T	19	45411941	0.154166	-0.0266723	0.00274373	2.39994e-22	461460
Body mass index (BMI)	Sex hormone-binding globulin levels	rs4307239	G	A	7	24354300	0.458941	0.0121413	0.00198816	1e-09	461460
Body mass index	Sex hormone-binding	rs4419475	T	A	4	96150044	0.407327	0.0111148	0.0020106	1.09999e-	461460

(BMI)	globulin levels							7		08	
Body mass index (BMI)	Sex hormone-binding globulin levels	rs4444317	G	A	15	92573234	0.216141	-0.0161604	0.00242104	2.49977e-11	461460
Body mass index (BMI)	Sex hormone-binding globulin levels	rs4456769	T	C	20	25190777	0.333444	0.0146111	0.00210138	3.59998e-12	461460
Body mass index (BMI)	Sex hormone-binding globulin levels	rs4477562	T	C	13	54104968	0.128644	0.0296118	0.00298011	2.90001e-23	461460
Body mass index (BMI)	Sex hormone-binding globulin levels	rs4482463	A	C	2	205375909	0.923002	-0.0313175	0.00370692	2.99985e-17	461460
Body mass index (BMI)	Sex hormone-binding globulin levels	rs45486197	A	G	19	2244849	0.065685	0.0257666	0.00403737	1.7e-10	461460
Body mass index (BMI)	Sex hormone-binding globulin levels	rs4605363	C	A	2	229010960	0.341595	0.0163638	0.00207733	3.29989e-15	461460
Body mass index (BMI)	Sex hormone-binding globulin levels	rs4648450	A	C	1	2723214	0.466824	-0.014837	0.00198775	8.4004e-14	461460
Body mass index (BMI)	Sex hormone-binding globulin levels	rs4658403	T	C	1	243832560	0.833659	-0.0188877	0.00264997	1e-12	461460
Body mass	Sex hormone-b	rs4672	T	C	2	60217457	0.336228	0.013	0.00208669	8.0001	461460

index (BMI)	inding globulin levels	338						5646		8e-11	
Body mass index (BMI)	Sex hormone-binding globulin levels	rs4722398	T	C	7	3125220	0.136137	0.0186993	0.00287545	7.89951e-11	461460
Body mass index (BMI)	Sex hormone-binding globulin levels	rs4790292	A	C	17	1824305	0.153693	-0.0254509	0.00275603	2.60016e-20	461460
Body mass index (BMI)	Sex hormone-binding globulin levels	rs4820410	G	A	22	40690385	0.345317	-0.0177457	0.00208527	1.69981e-17	461460
Body mass index (BMI)	Sex hormone-binding globulin levels	rs4832298	T	C	2	86764004	0.686142	-0.015965	0.00212268	5.40008e-14	461460
Body mass index (BMI)	Sex hormone-binding globulin levels	rs4876611	G	A	8	116671848	0.720243	0.0197549	0.00220502	3.29989e-19	461460
Body mass index (BMI)	Sex hormone-binding globulin levels	rs4929923	C	T	11	8639200	0.645204	0.0189424	0.00206436	4.49987e-20	461460
Body mass index (BMI)	Sex hormone-binding globulin levels	rs5011579	G	C	16	69187318	0.715152	0.0140268	0.00219322	1.6e-10	461460
Body mass index (BMI)	Sex hormone-binding globulin levels	rs512121	C	T	18	7548501	0.192054	-0.0159359	0.00252176	2.59998e-10	461460
Body	Sex	rs5	G	A	3	1731	0.5278	0.0	0.0019	1.2	461

mass index (BMI)	hormone-binding globulin levels	29200				14305	29	168965	785	9987e-17	460
Body mass index (BMI)	Sex hormone-binding globulin levels	rs55726687	A	G	12	991306	0.209709	0.02483	0.00242628	1.39991e-24	461460
Body mass index (BMI)	Sex hormone-binding globulin levels	rs558887	G	A	11	28712741	0.307507	-0.0129976	0.00214881	1.5e-09	461460
Body mass index (BMI)	Sex hormone-binding globulin levels	rs559231	T	G	18	39644247	0.393053	0.0134907	0.00203562	3.40017e-11	461460
Body mass index (BMI)	Sex hormone-binding globulin levels	rs56038322	A	G	3	69925128	0.31062	0.0139276	0.00214833	8.99912e-11	461460
Body mass index (BMI)	Sex hormone-binding globulin levels	rs56094641	G	A	16	53806453	0.404564	0.0734967	0.00201412	1e-200	461460
Body mass index (BMI)	Sex hormone-binding globulin levels	rs56143236	T	C	3	157020444	0.256388	0.0126667	0.00226533	2.30001e-08	461460
Body mass index (BMI)	Sex hormone-binding globulin levels	rs56203622	C	T	9	131040874	0.145529	0.0179769	0.00280183	1.40001e-10	461460
Body mass index (BMI)	Sex hormone-binding globulin levels	rs56352336	C	T	19	19352155	0.154894	-0.016327	0.00275021	2.90001e-09	461460

Body mass index (BMI)	Sex hormone-binding globulin levels	rs56399737	T	C	13	33381721	0.449124	-0.0161325	0.00199646	6.4003e-16	461460
Body mass index (BMI)	Sex hormone-binding globulin levels	rs56858768	A	G	13	86511730	0.296861	0.0159188	0.00217378	2.39994e-13	461460
Body mass index (BMI)	Sex hormone-binding globulin levels	rs56893062	G	T	8	25662655	0.303342	0.0125141	0.00215383	6.19998e-09	461460
Body mass index (BMI)	Sex hormone-binding globulin levels	rs56930105	T	C	2	10982487	0.139295	0.0157557	0.00286187	3.69999e-08	461460
Body mass index (BMI)	Sex hormone-binding globulin levels	rs57636386	C	T	18	58048295	0.08383	-0.0412553	0.00358326	1.10002e-30	461460
Body mass index (BMI)	Sex hormone-binding globulin levels	rs58862095	T	C	7	75081418	0.419266	-0.0229877	0.00200786	2.39994e-30	461460
Body mass index (BMI)	Sex hormone-binding globulin levels	rs59068084	T	G	4	113256737	0.410232	0.0110552	0.00201048	3.79997e-08	461460
Body mass index (BMI)	Sex hormone-binding globulin levels	rs59227842	G	A	11	43692423	0.31149	0.0229675	0.00215313	1.50003e-26	461460
Body mass index (BMI)	Sex hormone-binding globulin	rs594024	C	T	11	69443822	0.554162	-0.014677	0.00199081	1.69981e-13	461460

	levels							1			
Body mass index (BMI)	Sex hormone-binding globulin levels	rs6023655	G	A	20	53479658	0.765741	-0.0147346	0.00234997	3.59998e-10	461460
Body mass index (BMI)	Sex hormone-binding globulin levels	rs60764613	T	G	18	1839911	0.144883	0.0202099	0.00282688	1.10002e-13	461460
Body mass index (BMI)	Sex hormone-binding globulin levels	rs61740466	A	G	1	19934900	0.237203	-0.0135153	0.00231885	5.60003e-09	461460
Body mass index (BMI)	Sex hormone-binding globulin levels	rs61813324	T	C	1	156049877	0.135728	0.0290259	0.00292038	2.80027e-23	461460
Body mass index (BMI)	Sex hormone-binding globulin levels	rs61828641	A	G	1	174321997	0.109255	0.0224585	0.00315913	1.20005e-12	461460
Body mass index (BMI)	Sex hormone-binding globulin levels	rs61871615	T	C	10	102487140	0.091551	-0.0267159	0.00359269	1e-13	461460
Body mass index (BMI)	Sex hormone-binding globulin levels	rs61903695	G	A	11	89922417	0.254964	0.0166235	0.00227115	2.49977e-13	461460
Body mass index (BMI)	Sex hormone-binding globulin levels	rs61992671	G	A	14	101531854	0.491195	-0.0161925	0.00206924	5.10035e-15	461460
Body mass index	Sex hormone-binding	rs6200778	A	G	15	78029797	0.265091	-0.0167	0.00224215	9.3994e-1	461460

(BMI)	globulin levels	2						007		4	
Body mass index (BMI)	Sex hormone-binding globulin levels	rs62020775	A	T	15	89960286	0.14168	-0.0165665	0.0028635	7.19996e-09	461460
Body mass index (BMI)	Sex hormone-binding globulin levels	rs62072006	C	A	17	52938468	0.144541	0.0156492	0.00282374	2.99999e-08	461460
Body mass index (BMI)	Sex hormone-binding globulin levels	rs62107261	C	T	2	422144	0.048327	-0.0911559	0.00460882	4.60045e-87	461460
Body mass index (BMI)	Sex hormone-binding globulin levels	rs62176243	T	A	2	166190881	0.244987	-0.0149699	0.00228878	6.09958e-11	461460
Body mass index (BMI)	Sex hormone-binding globulin levels	rs62190049	C	G	2	182566998	0.390446	-0.0111798	0.00203614	4e-08	461460
Body mass index (BMI)	Sex hormone-binding globulin levels	rs62241847	G	A	3	20466465	0.314443	-0.0124263	0.00212897	5.30005e-09	461460
Body mass index (BMI)	Sex hormone-binding globulin levels	rs62246311	A	G	3	9498143	0.102364	0.0207539	0.00325487	1.79999e-10	461460
Body mass index (BMI)	Sex hormone-binding globulin levels	rs62379271	G	T	5	105870033	0.578511	0.0117237	0.00200557	5e-09	461460
Body mass	Sex hormone-b	rs6240	A	T	6	33530346	0.268584	0.014	0.00222463	8.4996	461460

index (BMI)	inding globulin levels	7562						4393		3e-11	
Body mass index (BMI)	Sex hormone-binding globulin levels	rs6265	T	C	11	27679916	0.188472	-0.0399185	0.00252719	3.29989e-56	461460
Body mass index (BMI)	Sex hormone-binding globulin levels	rs6430068	A	G	2	145627927	0.108559	0.0186066	0.0031882	5.30005e-09	461460
Body mass index (BMI)	Sex hormone-binding globulin levels	rs6444950	A	G	3	170602073	0.237474	0.0158525	0.00232007	8.30042e-12	461460
Body mass index (BMI)	Sex hormone-binding globulin levels	rs6545714	A	G	2	59307725	0.601448	-0.0205219	0.00201573	2.39994e-24	461460
Body mass index (BMI)	Sex hormone-binding globulin levels	rs6560906	C	T	12	133414054	0.691874	-0.012198	0.00214136	1.2e-08	461460
Body mass index (BMI)	Sex hormone-binding globulin levels	rs6561937	A	T	13	58257667	0.753587	-0.015934	0.00230346	4.60045e-12	461460
Body mass index (BMI)	Sex hormone-binding globulin levels	rs6575340	A	G	14	94023972	0.636038	0.0207333	0.00206189	8.69961e-24	461460
Body mass index (BMI)	Sex hormone-binding globulin levels	rs66679256	T	C	4	18351898	0.445826	0.0148864	0.00198759	6.89922e-14	461460
Body	Sex	rs6	G	A	1	4767	0.5826	-0.	0.0019	1.5	461

mass index (BMI)	hormone-binding globulin levels	669341				8458	79	0170287	986	9993e-17	460
Body mass index (BMI)	Sex hormone-binding globulin levels	rs6682438	C	T	1	33784146	0.673081	0.0131586	0.00210047	3.69999e-10	461460
Body mass index (BMI)	Sex hormone-binding globulin levels	rs6705567	C	T	2	55320173	0.37596	-0.0146168	0.00204919	9.79941e-13	461460
Body mass index (BMI)	Sex hormone-binding globulin levels	rs6707827	G	A	2	100123030	0.703627	0.0119437	0.00217332	3.89996e-08	461460
Body mass index (BMI)	Sex hormone-binding globulin levels	rs6710091	G	C	2	239597	0.348174	-0.0116835	0.00206707	1.6e-08	461460
Body mass index (BMI)	Sex hormone-binding globulin levels	rs6713781	C	G	2	40291940	0.401882	-0.0135736	0.00202799	2.19989e-11	461460
Body mass index (BMI)	Sex hormone-binding globulin levels	rs6725931	T	C	2	220205146	0.847658	0.0191046	0.00274403	3.29989e-12	461460
Body mass index (BMI)	Sex hormone-binding globulin levels	rs6744646	G	A	2	628504	0.828299	0.0554684	0.00261213	4.49987e-100	461460
Body mass index (BMI)	Sex hormone-binding globulin levels	rs6752979	A	G	2	81741750	0.31688	0.0125235	0.00211721	3.29997e-09	461460

Body mass index (BMI)	Sex hormone-binding globulin levels	rs67609008	C	T	10	126640936	0.283614	0.0170863	0.0022021	8.60003e-15	461460
Body mass index (BMI)	Sex hormone-binding globulin levels	rs6769617	T	A	3	62687746	0.66386	-0.0135754	0.00208902	8.10028e-11	461460
Body mass index (BMI)	Sex hormone-binding globulin levels	rs6774894	A	T	3	196116393	0.358148	0.0133088	0.00205674	9.70063e-11	461460
Body mass index (BMI)	Sex hormone-binding globulin levels	rs6777784	T	G	3	62376645	0.61678	0.0116125	0.00202753	1e-08	461460
Body mass index (BMI)	Sex hormone-binding globulin levels	rs6831088	A	G	4	20257769	0.640139	-0.0115222	0.00200591	2.19999e-08	461460
Body mass index (BMI)	Sex hormone-binding globulin levels	rs6843852	T	C	4	162132758	0.507861	0.0130897	0.00197607	3.50026e-11	461460
Body mass index (BMI)	Sex hormone-binding globulin levels	rs6909685	T	C	6	97753952	0.326809	-0.014612	0.00211352	4.70002e-12	461460
Body mass index (BMI)	Sex hormone-binding globulin levels	rs6922607	G	A	6	142703483	0.189847	0.0148848	0.00251479	3.2e-09	461460
Body mass index (BMI)	Sex hormone-binding globulin	rs6938973	C	T	6	98421721	0.601456	0.0182223	0.00201872	1.80011e-19	461460

	levels										
Body mass index (BMI)	Sex hormone-binding globulin levels	rs6950388	A	G	7	1270699	0.795086	0.0155219	0.00244806	2.30001e-10	461460
Body mass index (BMI)	Sex hormone-binding globulin levels	rs6962980	C	A	7	113452183	0.556019	-0.0159568	0.00198814	1e-15	461460
Body mass index (BMI)	Sex hormone-binding globulin levels	rs698147	G	A	5	3513485	0.543596	-0.0128512	0.00198514	9.60064e-11	461460
Body mass index (BMI)	Sex hormone-binding globulin levels	rs7024334	G	T	9	109072075	0.77914	-0.0138177	0.00238338	6.69993e-09	461460
Body mass index (BMI)	Sex hormone-binding globulin levels	rs7027304	T	C	9	129408290	0.652661	0.0145492	0.00208703	3.10027e-12	461460
Body mass index (BMI)	Sex hormone-binding globulin levels	rs7034554	G	A	9	37081301	0.373825	-0.0129826	0.00204252	2.1e-10	461460
Body mass index (BMI)	Sex hormone-binding globulin levels	rs704061	C	T	12	89771903	0.45505	0.0146374	0.00198552	1.69981e-13	461460
Body mass index (BMI)	Sex hormone-binding globulin levels	rs7070670	T	C	10	61842645	0.327923	-0.0123274	0.00211928	5.99998e-09	461460
Body mass index	Sex hormone-binding	rs7081254	C	T	10	132955696	0.205739	-0.0142	0.00245216	6.19998e-	461460

(BMI)	globulin levels							516		09	
Body mass index (BMI)	Sex hormone-binding globulin levels	rs7124681	A	C	11	47529947	0.408353	0.0256977	0.0020063	1.50003e-37	461460
Body mass index (BMI)	Sex hormone-binding globulin levels	rs7132908	A	G	12	50263148	0.384458	0.0297904	0.00203363	1.39991e-48	461460
Body mass index (BMI)	Sex hormone-binding globulin levels	rs71495038	A	G	10	33971383	0.076936	0.0277998	0.00370969	6.70039e-14	461460
Body mass index (BMI)	Sex hormone-binding globulin levels	rs7201895	A	G	16	407723	0.354291	-0.0149781	0.00207999	6.00067e-13	461460
Body mass index (BMI)	Sex hormone-binding globulin levels	rs7206608	G	C	16	82872628	0.32162	0.0135122	0.00211797	1.79999e-10	461460
Body mass index (BMI)	Sex hormone-binding globulin levels	rs7218014	C	T	17	65832016	0.197305	0.0189524	0.00249216	2.90001e-14	461460
Body mass index (BMI)	Sex hormone-binding globulin levels	rs7232171	T	G	18	31251221	0.58263	0.0121234	0.00200821	8e-10	461460
Body mass index (BMI)	Sex hormone-binding globulin levels	rs723672	T	C	12	2161561	0.43151	0.0111273	0.00200698	2.9999e-08	461460
Body mass	Sex hormone-b	rs7259	C	T	19	47562509	0.596062	0.021	0.00203628	6.5993	461460

index (BMI)	inding globulin levels	070						869		3e-27	
Body mass index (BMI)	Sex hormone-binding globulin levels	rs72634826	A	G	1	1601052	0.259858	-0.021277	0.00228001	1e-20	461460
Body mass index (BMI)	Sex hormone-binding globulin levels	rs72649373	C	T	4	80609966	0.143188	0.0178062	0.00287805	6.1e-10	461460
Body mass index (BMI)	Sex hormone-binding globulin levels	rs72673947	G	A	8	11884379	0.107031	0.0218864	0.00321496	9.8992e-12	461460
Body mass index (BMI)	Sex hormone-binding globulin levels	rs72892910	T	G	6	50816887	0.172232	0.0387798	0.00262053	1.50003e-49	461460
Body mass index (BMI)	Sex hormone-binding globulin levels	rs72976986	A	G	19	4050424	0.190123	-0.0232257	0.00254712	7.59976e-20	461460
Body mass index (BMI)	Sex hormone-binding globulin levels	rs73026725	A	C	19	31017686	0.153534	-0.022318	0.00275052	4.90004e-16	461460
Body mass index (BMI)	Sex hormone-binding globulin levels	rs73052033	C	T	3	185828465	0.18493	-0.0303929	0.00254634	7.70016e-33	461460
Body mass index (BMI)	Sex hormone-binding globulin levels	rs7306534	A	G	12	68107914	0.621712	-0.0112754	0.00205437	4.09996e-08	461460
Body	Sex	rs7	C	T	7	7157	0.2051	-0.	0.0024	3.0	461

mass index (BMI)	hormone-binding globulin levels	3124396				9606	44	0154472	548	9999e-10	460
Body mass index (BMI)	Sex hormone-binding globulin levels	rs73142879	T	C	20	51195932	0.192297	-0.0266936	0.00252257	3.59998e-26	461460
Body mass index (BMI)	Sex hormone-binding globulin levels	rs73193736	G	A	12	108294381	0.243934	-0.0177165	0.00232002	2.19989e-14	461460
Body mass index (BMI)	Sex hormone-binding globulin levels	rs73213484	T	A	4	28489339	0.141236	-0.0225766	0.0028364	1.69981e-15	461460
Body mass index (BMI)	Sex hormone-binding globulin levels	rs7331420	A	G	13	99236471	0.285276	-0.0143702	0.00220081	6.59933e-11	461460
Body mass index (BMI)	Sex hormone-binding globulin levels	rs7357754	G	A	9	92207308	0.500139	0.0141146	0.00198362	1.10002e-12	461460
Body mass index (BMI)	Sex hormone-binding globulin levels	rs73601548	T	C	10	18549889	0.114527	0.0177354	0.00311639	1.29999e-08	461460
Body mass index (BMI)	Sex hormone-binding globulin levels	rs73985439	C	A	2	212299249	0.307299	0.0136616	0.00214059	1.7e-10	461460
Body mass index (BMI)	Sex hormone-binding globulin levels	rs7442885	G	C	5	87682877	0.214049	-0.0228163	0.00241251	3.19963e-21	461460

Body mass index (BMI)	Sex hormone-binding globulin levels	rs745249	T	C	2	105460333	0.281945	0.0176118	0.00219724	1.10002e-15	461460
Body mass index (BMI)	Sex hormone-binding globulin levels	rs74750282	C	T	7	114744463	0.086623	-0.0196382	0.00352561	2.5e-08	461460
Body mass index (BMI)	Sex hormone-binding globulin levels	rs7498665	G	A	16	28883241	0.399659	0.0268646	0.00201997	2.29985e-40	461460
Body mass index (BMI)	Sex hormone-binding globulin levels	rs7516554	T	C	1	210301331	0.399941	0.0120066	0.00201565	2.59998e-09	461460
Body mass index (BMI)	Sex hormone-binding globulin levels	rs7519259	A	G	1	66434743	0.52836	0.0140028	0.00198381	1.69981e-12	461460
Body mass index (BMI)	Sex hormone-binding globulin levels	rs754635	G	C	3	42305131	0.886582	0.0219925	0.0031093	1.50003e-12	461460
Body mass index (BMI)	Sex hormone-binding globulin levels	rs75499503	T	C	6	26145217	0.220101	-0.0180331	0.00241845	8.9002e-14	461460
Body mass index (BMI)	Sex hormone-binding globulin levels	rs7571496	G	A	2	6169351	0.260545	-0.0158916	0.00225454	1.80011e-12	461460
Body mass index (BMI)	Sex hormone-binding globulin	rs76183894	C	T	3	114371939	0.080747	-0.021958	0.00364437	1.7e-09	461460

	levels							4			
Body mass index (BMI)	Sex hormone-binding globulin levels	rs7619139	A	T	3	25110415	0.588616	0.0134537	0.00201061	2.19989e-11	461460
Body mass index (BMI)	Sex hormone-binding globulin levels	rs76702514	G	C	1	195148296	0.210623	-0.0164862	0.00243278	1.20005e-11	461460
Body mass index (BMI)	Sex hormone-binding globulin levels	rs7683836	A	G	4	180167906	0.557215	-0.0122469	0.00199427	8.19993e-10	461460
Body mass index (BMI)	Sex hormone-binding globulin levels	rs7708584	G	A	5	153543466	0.572344	-0.0159321	0.0019949	1.39991e-15	461460
Body mass index (BMI)	Sex hormone-binding globulin levels	rs7761673	A	T	6	70357368	0.21988	-0.0135915	0.00233901	1.29999e-08	461460
Body mass index (BMI)	Sex hormone-binding globulin levels	rs7762794	G	A	6	153380228	0.285408	0.0149077	0.00218606	9.09913e-12	461460
Body mass index (BMI)	Sex hormone-binding globulin levels	rs7776021	A	G	6	73742152	0.287599	0.0123701	0.00218251	1.40001e-08	461460
Body mass index (BMI)	Sex hormone-binding globulin levels	rs7802342	G	T	7	137435925	0.288566	0.0122748	0.00218145	1.79999e-08	461460
Body mass index	Sex hormone-binding	rs7805441	T	C	7	78121458	0.502261	0.01337	0.00198884	1.80011e-	461460

(BMI)	globulin levels							47		11	
Body mass index (BMI)	Sex hormone-binding globulin levels	rs784257	C	T	18	53397199	0.812541	0.0179315	0.00254968	1.99986e-12	461460
Body mass index (BMI)	Sex hormone-binding globulin levels	rs78605811	C	A	3	83631491	0.05405	-0.032737	0.00444673	1.8001e-13	461460
Body mass index (BMI)	Sex hormone-binding globulin levels	rs7893571	T	G	10	16750129	0.665897	0.0140553	0.00210152	2.29985e-11	461460
Body mass index (BMI)	Sex hormone-binding globulin levels	rs7925100	A	G	11	118941596	0.396117	0.014725	0.00200221	3.29989e-13	461460
Body mass index (BMI)	Sex hormone-binding globulin levels	rs7944782	G	T	11	130795698	0.509784	0.0157606	0.00198748	2.19998e-15	461460
Body mass index (BMI)	Sex hormone-binding globulin levels	rs7947143	A	G	11	64090422	0.163483	-0.0182782	0.00267522	8.30042e-12	461460
Body mass index (BMI)	Sex hormone-binding globulin levels	rs79780963	T	C	10	104952499	0.077428	0.0236803	0.00369591	1.5e-10	461460
Body mass index (BMI)	Sex hormone-binding globulin levels	rs7996639	A	G	13	97019090	0.449354	0.0145559	0.00200245	3.59998e-13	461460
Body mass	Sex hormone-b	rs8013	T	A	17	5286277	0.070324	0.021	0.00388113	3.7999	461460

index (BMI)	inding globulin levels	5274						339		7e-08	
Body mass index (BMI)	Sex hormone-binding globulin levels	rs8020365	A	T	14	79937216	0.220464	0.0251068	0.00239517	1e-25	461460
Body mass index (BMI)	Sex hormone-binding globulin levels	rs8024137	T	A	15	35837297	0.848207	0.0156329	0.00276778	1.6e-08	461460
Body mass index (BMI)	Sex hormone-binding globulin levels	rs8025516	G	T	15	95271872	0.645928	-0.0146558	0.00207578	1.6998e-12	461460
Body mass index (BMI)	Sex hormone-binding globulin levels	rs8089514	A	T	18	69224478	0.36868	0.0129888	0.00207588	3.89996e-10	461460
Body mass index (BMI)	Sex hormone-binding globulin levels	rs8112818	G	A	19	18812785	0.400329	-0.0206096	0.00202712	1.8001e-24	461460
Body mass index (BMI)	Sex hormone-binding globulin levels	rs8132491	A	G	21	40288577	0.313023	-0.0115375	0.00219317	2.3999e-12	461460
Body mass index (BMI)	Sex hormone-binding globulin levels	rs815163	C	T	1	190294726	0.563148	-0.0164885	0.00198582	1e-16	461460
Body mass index (BMI)	Sex hormone-binding globulin levels	rs852042	G	A	20	17091233	0.758575	-0.0131207	0.00231309	1.4000e-08	461460
Body	Sex	rs8	T	C	16	6965	0.4096	-0.	0.0020	1.2	461

mass index (BMI)	hormone-binding globulin levels	62320				1866	5	0231703	1345	0005e-30	460
Body mass index (BMI)	Sex hormone-binding globulin levels	rs909892	A	G	20	41982698	0.134817	-0.0184105	0.00291255	2.59998e-10	461460
Body mass index (BMI)	Sex hormone-binding globulin levels	rs923994	G	A	4	67802992	0.783195	-0.0145761	0.00240219	1.29999e-09	461460
Body mass index (BMI)	Sex hormone-binding globulin levels	rs9267671	A	G	6	31880480	0.060733	0.0261499	0.00413587	2.59998e-10	461460
Body mass index (BMI)	Sex hormone-binding globulin levels	rs9291822	T	C	5	64076515	0.514868	-0.014263	0.00199464	8.60003e-13	461460
Body mass index (BMI)	Sex hormone-binding globulin levels	rs9294260	A	G	6	83433228	0.476559	0.014782	0.00198818	1e-13	461460
Body mass index (BMI)	Sex hormone-binding globulin levels	rs9349235	T	C	6	42516718	0.410635	0.0111809	0.00200924	2.59998e-08	461460
Body mass index (BMI)	Sex hormone-binding globulin levels	rs935166	A	G	2	26949366	0.506805	-0.0161066	0.00197292	3.19996e-16	461460
Body mass index (BMI)	Sex hormone-binding globulin levels	rs9463175	T	C	6	9510030	0.338958	-0.0115	0.00210115	4.39997e-08	461460

Body mass index (BMI)	Sex hormone-binding globulin levels	rs9478496	C	T	6	154333183	0.164225	0.0181836	0.00267493	1.10002e-11	461460
Body mass index (BMI)	Sex hormone-binding globulin levels	rs9515446	G	A	13	112217108	0.447709	0.0151051	0.00199052	3.19963e-14	461460
Body mass index (BMI)	Sex hormone-binding globulin levels	rs9522180	T	C	13	111970212	0.553392	-0.0141095	0.00199325	1.50003e-12	461460
Body mass index (BMI)	Sex hormone-binding globulin levels	rs9571687	A	C	13	67472713	0.329363	-0.0135388	0.0021094	1.40001e-10	461460
Body mass index (BMI)	Sex hormone-binding globulin levels	rs9638713	G	A	7	14645949	0.97477	-0.0361735	0.00635399	1.2e-08	461460
Body mass index (BMI)	Sex hormone-binding globulin levels	rs9673839	G	A	16	76895693	0.490967	0.0130337	0.00198783	5.50047e-11	461460
Body mass index (BMI)	Sex hormone-binding globulin levels	rs9674487	G	C	17	80191995	0.001338	0.158445	0.0286171	3.09999e-08	461460
Body mass index (BMI)	Sex hormone-binding globulin levels	rs9830592	A	C	3	104631603	0.582421	0.0154849	0.00200164	1e-14	461460
Body mass index (BMI)	Sex hormone-binding globulin	rs9839081	A	G	3	123051230	0.325232	-0.011701	0.00214045	4.60002e-08	461460

	levels										
Body mass index (BMI)	Sex hormone-binding globulin levels	rs9843653	C	T	3	49920571	0.511652	0.0294509	0.00197538	2.90001e-50	461460
Body mass index (BMI)	Sex hormone-binding globulin levels	rs9876664	T	G	3	85806313	0.375389	-0.0180478	0.00204174	9.60064e-19	461460
Body mass index (BMI)	Sex hormone-binding globulin levels	rs9888533	T	C	13	107854612	0.538079	0.0120038	0.0020182	2.69998e-09	461460
Body mass index (BMI)	Sex hormone-binding globulin levels	rs9926784	C	T	16	19941968	0.184576	-0.023818	0.00254677	8.60003e-21	461460
Body mass index (BMI)	Sex hormone-binding globulin levels	rs9951619	G	T	18	56882326	0.767369	0.0144231	0.00235777	9.49992e-10	461460

Supplementary Table S4. Instruments for causal estimation from BioT level to BMI.

Exposure	Outcome	SNP	Effect allele	Other allele	Chromosome	Genetic position	Effect allele frequency	Beta	Standard error of beta	Pvalue	Sample size
Bioavailable testosterone levels	Body mass index (BMI)	rs10279715	G	A	7	40870935	0.462118	-0.0217388	0.00306173	3.10027e-13	178782
Bioavailable testosterone levels	Body mass index (BMI)	rs10738700	G	A	9	24973797	0.430636	-0.0204816	0.00300877	5.60015e-11	178782
Bioavailable testosterone levels	Body mass index (BMI)	rs10867084	C	T	9	140774721	0.489079	0.0226393	0.00304395	6.79986e-14	178782
Bioavailable testosterone levels	Body mass index (BMI)	rs10982156	A	T	9	117088064	0.069545	0.0477036	0.00622867	4.49987e-14	178782
Bioavailable testosterone levels	Body mass index (BMI)	rs112112195	G	A	3	24085166	0.495419	0.0182475	0.00306001	4.70002e-10	178782
Bioavailable testosterone levels	Body mass index (BMI)	rs12217694	C	G	4	104641064	0.05773	-0.0494985	0.00656267	1.20005e-14	178782
Bioavailable testosterone levels	Body mass index (BMI)	rs14492985	C	T	4	70572301	0.11256	-0.0249099	0.00483022	3.69999e-08	178782
Bioavailable testosterone levels	Body mass index (BMI)	rs14548805	A	T	4	104705578	0.03578	-0.0528064	0.00829446	8.60003e-10	178782
Bioavaila	Body	rs1	G	A	4	1047	0.0119	-0.	0.0146	2.8	178

ble	mass	152				7469	39	22	193	002	782
testostero	index	602				8		71		7e-	
ne levels	(BMI)	27						47		56	
Bioavaila	Body	rs1						0.0		5.9	
ble	mass	170						39		006	
testostero	index	337				4967	0.2696	65	0.0034	5e-	178
ne levels	(BMI)	6	T	C	22	8713	62	26	4869	32	782
Bioavaila	Body							0.0		2.2	
ble	mass	rs1						37		998	
testostero	index	264				3084	0.3523	94	0.0031	5e-	178
ne levels	(BMI)	332	C	G	6	5563	23	17	8829	25	782
Bioavaila	Body							0.0		1.3	
ble	mass	rs1						26		999	
testostero	index	272				6088	0.3866	76	0.0031	1e-	178
ne levels	(BMI)	131	C	T	14	6150	95	42	5783	17	782
								-0.			
Bioavaila	Body	rs1						05		6.8	
ble	mass	279						60		992	
testostero	index	648				9413	0.1768	93	0.0039	2e-	178
ne levels	(BMI)	8	A	C	11	1557	83	6	8259	46	782
Bioavaila	Body	rs1						0.0		1.9	
ble	mass	294						40		998	
testostero	index	464				7492	0.1987	87	0.0038	6e-	178
ne levels	(BMI)	9	G	A	17	228	32	54	2485	27	782
								-0.			
Bioavaila	Body	rs1						03		2.7	
ble	mass	302						08		002	
testostero	index	847				1171	0.5535	99	0.0030	3e-	178
ne levels	(BMI)	9	T	G	2	2075	1	4	7167	24	782
								-0.			
Bioavaila	Body	rs1						03		8.8	
ble	mass	306						20		003	
testostero	index	546				6166	0.1308	51	0.0045	5e-	178
ne levels	(BMI)	3	A	G	3	2996	52	9	199	13	782
Bioavaila	Body							0.0		4.9	
ble	mass	rs1						18		000	
testostero	index	383				8905	0.4242	98	0.0030	4e-	178
ne levels	(BMI)	5	A	C	15	6040	08	02	8562	10	782
Bioavaila	Body	rs1						-0.		8.6	
ble	mass	453						07		996	
testostero	index	599				7850	0.0241	76	0.0106	1e-	178
ne levels	(BMI)	38	C	G	7	4431	11	46	751	15	782
Bioavaila	Body	rs1				2937	0.0185	-0.	0.0113	1e-	178
ble	mass	461	T	C	11	8978	66	08	221	15	782

testosterone levels	index (BMI)	15416						78742			
Bioavailable testosterone levels	Body mass index (BMI)	rs17703883	C	T	15	51530097	0.255265	0.0442784	0.00350043	7.50067e-40	178782
Bioavailable testosterone levels	Body mass index (BMI)	rs2011425	G	T	2	234627608	0.079615	-0.0502524	0.00565644	6.4003e-20	178782
Bioavailable testosterone levels	Body mass index (BMI)	rs2038695	A	C	13	100559123	0.550327	-0.0227854	0.00308986	2.60016e-15	178782
Bioavailable testosterone levels	Body mass index (BMI)	rs2090409	A	C	9	108967088	0.315943	-0.0309321	0.0032844	1.69981e-21	178782
Bioavailable testosterone levels	Body mass index (BMI)	rs2184968	C	T	6	126760994	0.451	0.0202431	0.00305595	9.09913e-11	178782
Bioavailable testosterone levels	Body mass index (BMI)	rs2631864	G	A	8	21112084	0.103625	0.0331054	0.00501603	8.30042e-12	178782
Bioavailable testosterone levels	Body mass index (BMI)	rs2764772	A	T	16	20060653	0.33405	0.0370436	0.003233	5.40008e-33	178782
Bioavailable testosterone levels	Body mass index (BMI)	rs2961853	C	T	5	165932048	0.468671	0.0191152	0.00305362	3.2e-10	178782
Bioavailable testosterone levels	Body mass index (BMI)	rs3020421	A	G	6	152348122	0.327194	-0.0185099	0.0032529	1e-08	178782
Bioavaila	Body	rs3	C	T	3	1072	0.0756	-0.	0.0059	2.1	178

ble testosterone levels	mass index (BMI)	4040779				35109	71	0348252	1069	9999e-09	782
Bioavailable testosterone levels	Body mass index (BMI)	rs34192788	A	T	6	17416258	0.309674	-0.0198155	0.00332811	4.20001e-09	178782
Bioavailable testosterone levels	Body mass index (BMI)	rs35737316	T	C	1	204161534	0.24569	0.0402393	0.00354903	4.70002e-32	178782
Bioavailable testosterone levels	Body mass index (BMI)	rs3742223	C	T	13	112725196	0.096604	-0.0433217	0.00521873	1e-16	178782
Bioavailable testosterone levels	Body mass index (BMI)	rs4562360	G	A	8	61704817	0.756947	0.0323526	0.00355024	3.59998e-20	178782
Bioavailable testosterone levels	Body mass index (BMI)	rs4919686	C	A	10	104592249	0.289688	-0.0234107	0.00335461	3.90032e-13	178782
Bioavailable testosterone levels	Body mass index (BMI)	rs503542	G	A	11	118590743	0.450311	0.0183001	0.00300761	5.80003e-10	178782
Bioavailable testosterone levels	Body mass index (BMI)	rs55795858	C	T	7	146123500	0.329414	0.0179944	0.003298	1.5e-08	178782
Bioavailable testosterone levels	Body mass index (BMI)	rs57606486	A	G	14	35796645	0.026698	0.0532939	0.00951357	2.1e-08	178782
Bioavailable testosterone levels	Body mass index (BMI)	rs58879558	C	T	17	44095467	0.229892	0.0225798	0.00361103	2.39999e-09	178782
Bioavaila	Body	rs6	G	T	16	7392	0.2126	0.0	0.0037	1.6	178

ble testosterone levels	mass index (BMI)	2041532				2719	19	233361	2844	e-10	782
Bioavailable testosterone levels	Body mass index (BMI)	rs62465144	C	T	7	72883106	0.197582	-0.0232167	0.00382645	1.6e-09	178782
Bioavailable testosterone levels	Body mass index (BMI)	rs6486542	T	C	12	130952209	0.429429	-0.0261149	0.00309397	2.90001e-18	178782
Bioavailable testosterone levels	Body mass index (BMI)	rs6600895	C	T	4	69983412	0.457994	-0.0174488	0.00300472	3.2e-09	178782
Bioavailable testosterone levels	Body mass index (BMI)	rs6718154	T	C	2	180497923	0.274755	0.0322455	0.00340933	1.20005e-22	178782
Bioavailable testosterone levels	Body mass index (BMI)	rs7089031	G	T	10	101693155	0.393477	-0.0174188	0.0031234	3.2e-08	178782
Bioavailable testosterone levels	Body mass index (BMI)	rs714786	A	G	3	53789022	0.373237	-0.0213182	0.00314574	3.69999e-13	178782
Bioavailable testosterone levels	Body mass index (BMI)	rs71519251	A	G	1	163251833	0.166865	0.0240397	0.00414069	1.5e-09	178782
Bioavailable testosterone levels	Body mass index (BMI)	rs7265992	A	G	20	33525407	0.178841	-0.0317217	0.00402147	4.00037e-16	178782
Bioavailable testostero	Body mass index	rs745486	T	C	9	11242155	0.281397	-0.0213	0.00338763	1.2e-10	178782

ne levels	(BMI)							054			
Bioavailable testosterone levels	Body mass index (BMI)	rs7454964	C	T	6	52728059	0.571586	-0.0171983	0.00308619	1.79999e-08	178782
Bioavailable testosterone levels	Body mass index (BMI)	rs7679843	G	C	4	22028079	0.094982	0.0501614	0.00533198	3.80014e-22	178782
Bioavailable testosterone levels	Body mass index (BMI)	rs7857865	A	G	9	1957974	0.475348	0.0173188	0.00306171	5.80003e-09	178782
Bioavailable testosterone levels	Body mass index (BMI)	rs7912521	C	T	10	67262089	0.415588	0.0611999	0.00308282	6.20012e-94	178782
Bioavailable testosterone levels	Body mass index (BMI)	rs7928369	T	C	11	125095221	0.721798	-0.0240248	0.00339392	1.10002e-13	178782
Bioavailable testosterone levels	Body mass index (BMI)	rs8076703	T	C	17	75612643	0.704135	-0.0238709	0.00333901	1e-12	178782
Bioavailable testosterone levels	Body mass index (BMI)	rs912202	C	G	9	77225603	0.343328	0.0401986	0.00320718	2.49977e-38	178782
Bioavailable testosterone levels	Body mass index (BMI)	rs9322822	T	C	6	105369598	0.3215	-0.0487092	0.00325272	2.09991e-52	178782
Bioavailable testosterone levels	Body mass index (BMI)	rs950716	G	A	5	135680540	0.137381	-0.0336833	0.00442472	3.10027e-14	178782
Bioavaila	Body	rs9	T	G	3	2880	0.7208	0.0	0.0033	4.1	178

ble	mass	824				7441	96	25	9312	001	782
testostero	index	196						98		5e-	
ne levels	(BMI)							6		16	
Bioavaila	Body							0.0		2.8	
ble	mass	rs9						55		002	
testostero	index	986				1501	0.5068	60	0.0030	7e-	178
ne levels	(BMI)	829	A	G	7	9259	65	73	5395	76	782

Supplementary Table S5. Instruments for causal estimation from estradiol level to BMI.

Exposure	Outcome	SNP	Effect allele	Other allele	Chromosome	Genetic position	Effect allele frequency	Beta	Standard error of beta	Pvalue	Sample size
Estradiol levels	Body mass index (BMI)	rs10006452	C	T	4	69977808	0.48021	-0.00728098	0.000844823	5.30029e-18	206927
Estradiol levels	Body mass index (BMI)	rs10491431	A	C	5	35968000	0.141	0.00774443	0.00121717	1.79999e-10	206927
Estradiol levels	Body mass index (BMI)	rs112881196	G	C	2	31982811	0.039212	0.0249518	0.00219839	5.60015e-30	206927
Estradiol levels	Body mass index (BMI)	rs117826558	T	C	22	46770756	0.033971	0.0136375	0.00243043	2.69998e-08	206927
Estradiol levels	Body mass index (BMI)	rs2547234	C	T	19	48383906	0.834781	-0.00760122	0.00113891	2.70023e-11	206927
Estradiol levels	Body mass index (BMI)	rs34019140	A	G	14	106527500	0.435748	-0.011661	0.000862566	6.89922e-42	206927
Estradiol levels	Body mass index (BMI)	rs3751591	G	A	15	51606710	0.166931	0.0077766	0.00113687	6.59933e-12	206927
Estradiol levels	Body mass index (BMI)	rs56196860	A	C	12	2908330	0.031492	0.0209011	0.0024192	2.60016e-18	206927
Estradiol levels	Body mass index (BMI)	rs6571526	A	C	9	136139265	0.338866	-0.00835302	0.00089229	5.50047e-21	206927
Estradiol	Body mass	rs71735	T	C	15	51533736	0.64871	0.01585	0.000886215	3.5999	206927

levels	index (BMI)	95						26		8e-72	
Estradiol levels	Body mass index (BMI)	rs727428	C	T	17	7537792	0.557221	0.00566325	0.000852078	1.8001e-11	206927

Supplementary Table S6. Instruments for causal estimation from SHBG level to BMI.

Exposure	Outcome	SNP	Effect allele	Other allele	Chromosome	Genetic position	Effect allele frequency	Beta	Standard error of beta	Pvalue	Sample size
Sex hormone-binding globulin levels	Body mass index (BMI)	rs10027275	C	G	4	148981496	0.74126	-0.0125534	0.00141198	1.69981e-21	180726
Sex hormone-binding globulin levels	Body mass index (BMI)	rs10041660	C	T	5	122650224	0.50476	0.00755274	0.00124541	8.19993e-10	180726
Sex hormone-binding globulin levels	Body mass index (BMI)	rs10107182	T	C	8	59392737	0.663202	0.0124022	0.00130257	2.80027e-22	180726
Sex hormone-binding globulin levels	Body mass index (BMI)	rs10737683	A	G	1	196894829	0.236779	-0.010124	0.00144072	4.30031e-12	180726
Sex hormone-binding globulin levels	Body mass index (BMI)	rs10868080	A	T	9	86626769	0.744477	-0.0213279	0.00140815	1.50003e-57	180726
Sex hormone-binding globulin levels	Body mass index (BMI)	rs10881582	A	G	9	137256078	0.238037	0.0133501	0.00145058	9.30037e-21	180726
Sex hormone-binding globulin levels	Body mass index (BMI)	rs10895277	G	A	11	102084940	0.340806	-0.00961547	0.00130251	8.9002e-15	180726
Sex hormone-b	Body mass	rs1107	A	C	16	15148646	0.296081	0.0092	0.00135476	8.0001	180726

inding globulin levels	index (BMI)	5253						2345		8e-12	
Sex hormone-binding globulin levels	Body mass index (BMI)	rs1111127274	A	G	12	102838128	0.737279	-0.00731072	0.00138631	4.60002e-08	180726
Sex hormone-binding globulin levels	Body mass index (BMI)	rs11752158	T	C	4	170994894	0.096496	0.0127725	0.0020799	2.80027e-11	180726
Sex hormone-binding globulin levels	Body mass index (BMI)	rs11981233	G	T	19	50016479	0.078984	0.0261638	0.00227727	8.19974e-33	180726
Sex hormone-binding globulin levels	Body mass index (BMI)	rs112035922	T	C	13	115047464	0.231857	-0.008812	0.00146895	2.30001e-10	180726
Sex hormone-binding globulin levels	Body mass index (BMI)	rs113248443	G	A	10	66102542	0.048294	-0.0143674	0.00294711	2.90001e-08	180726
Sex hormone-binding globulin levels	Body mass index (BMI)	rs114165349	C	G	1	27021913	0.023158	-0.0871136	0.00408954	3.49945e-111	180726
Sex hormone-binding globulin levels	Body mass index (BMI)	rs11564722	T	C	11	2178330	0.23874	0.00955307	0.00146515	1.20005e-13	180726
Sex hormone-binding globulin levels	Body mass index (BMI)	rs11610256	T	C	12	111734121	0.216665	0.00867838	0.00149523	2.1e-09	180726
Sex	Body	rs1	T	C	14	2487	0.4536	-0.	0.0012	1.3	180

hormone-binding globulin levels	mass index (BMI)	1621792				1926	47	012749	4244	9991e-25	726
Sex hormone-binding globulin levels	Body mass index (BMI)	rs116338429	T	C	13	114767040	0.170752	0.00985678	0.00172794	1.29999e-09	180726
Sex hormone-binding globulin levels	Body mass index (BMI)	rs11666245	A	G	19	38229926	0.047306	-0.01633325	0.0029014	2.19999e-09	180726
Sex hormone-binding globulin levels	Body mass index (BMI)	rs11734408	G	A	4	23882519	0.293256	0.00767697	0.00134935	3.29997e-10	180726
Sex hormone-binding globulin levels	Body mass index (BMI)	rs11739158	T	C	5	72927292	0.427811	0.00829379	0.00124625	2.09991e-12	180726
Sex hormone-binding globulin levels	Body mass index (BMI)	rs11743810	T	C	5	137802404	0.560069	0.00893646	0.00124066	1.99986e-12	180726
Sex hormone-binding globulin levels	Body mass index (BMI)	rs117664177	T	C	12	101036562	0.01211	-0.051561	0.00573803	3.69999e-22	180726
Sex hormone-binding globulin levels	Body mass index (BMI)	rs117734486	T	C	8	81061523	0.061081	-0.0133401	0.00261459	1.2e-08	180726
Sex hormone-binding globulin levels	Body mass index (BMI)	rs117921873	G	A	8	81473835	0.066515	0.021022	0.00250814	4.70002e-18	180726

Sex hormone-binding globulin levels	Body mass index (BMI)	rs118037027	T	A	12	21203424	0.040459	0.0222618	0.00313784	3.69999e-13	180726
Sex hormone-binding globulin levels	Body mass index (BMI)	rs11856926	A	G	15	96223649	0.448829	-0.011587	0.00124664	1.9002e-22	180726
Sex hormone-binding globulin levels	Body mass index (BMI)	rs11870307	G	A	17	7617787	0.780506	0.0526994	0.00145305	1e-200	180726
Sex hormone-binding globulin levels	Body mass index (BMI)	rs12138136	A	T	1	149844305	0.086596	0.0152389	0.00220676	6.29941e-14	180726
Sex hormone-binding globulin levels	Body mass index (BMI)	rs1229492	C	T	7	81564122	0.731518	-0.0114669	0.00139655	6.29941e-18	180726
Sex hormone-binding globulin levels	Body mass index (BMI)	rs12605524	G	A	18	22073551	0.28752	-0.00764797	0.00137562	1.2e-08	180726
Sex hormone-binding globulin levels	Body mass index (BMI)	rs12611046	A	G	19	14291704	0.150636	-0.009968	0.00177915	7.00003e-09	180726
Sex hormone-binding globulin levels	Body mass index (BMI)	rs12694450	C	T	2	220019638	0.675226	-0.00706409	0.0013572	3.79997e-08	180726
Sex hormone-binding globulin	Body mass index (BMI)	rs12696304	G	C	3	169481271	0.265645	0.00813109	0.00139236	1.7e-09	180726

levels											
Sex hormone-binding globulin levels	Body mass index (BMI)	rs12797706	A	G	11	65561369	0.234588	0.0120832	0.00146721	6.09958e-17	180726
Sex hormone-binding globulin levels	Body mass index (BMI)	rs12953087	T	C	17	59265201	0.795092	0.00827924	0.00148126	7.49998e-09	180726
Sex hormone-binding globulin levels	Body mass index (BMI)	rs12989596	T	G	2	148591232	0.291651	0.00791747	0.00135721	9.59997e-10	180726
Sex hormone-binding globulin levels	Body mass index (BMI)	rs13108218	G	A	4	3443931	0.617254	-0.0229943	0.00127338	1.20005e-79	180726
Sex hormone-binding globulin levels	Body mass index (BMI)	rs13150068	G	A	4	88203828	0.437637	-0.0207329	0.00123764	7.59976e-67	180726
Sex hormone-binding globulin levels	Body mass index (BMI)	rs13379043	C	T	14	74250126	0.280009	0.00828852	0.00139928	2.19999e-10	180726
Sex hormone-binding globulin levels	Body mass index (BMI)	rs1349852	C	A	4	69533217	0.47548	0.0203727	0.00123789	5.90065e-67	180726
Sex hormone-binding globulin levels	Body mass index (BMI)	rs138529890	A	G	2	32478354	0.037848	0.0252972	0.00327843	2.49977e-14	180726
Sex hormone-binding	Body mass index	rs1386115	C	T	5	56228040	0.076823	-0.014847	0.0023084	3.50026e-	180726

globulin levels	(BMI)	41						1		12	
Sex hormone-binding globulin levels	Body mass index (BMI)	rs144647926	A	G	1	235467607	0.087356	0.0123175	0.00217179	1.40001e-09	180726
Sex hormone-binding globulin levels	Body mass index (BMI)	rs1538648	G	A	10	94820860	0.503197	-0.00757485	0.00122235	8.4004e-13	180726
Sex hormone-binding globulin levels	Body mass index (BMI)	rs1736180	A	G	19	2796021	0.287261	0.0173849	0.00136123	2.29985e-41	180726
Sex hormone-binding globulin levels	Body mass index (BMI)	rs174529	C	T	11	61543961	0.375889	-0.00774839	0.00127239	4.20001e-10	180726
Sex hormone-binding globulin levels	Body mass index (BMI)	rs1782652	A	T	10	81074125	0.380625	-0.0127414	0.00127529	5.79963e-27	180726
Sex hormone-binding globulin levels	Body mass index (BMI)	rs1801689	C	A	17	64210580	0.029871	-0.03343	0.00350564	1.80011e-21	180726
Sex hormone-binding globulin levels	Body mass index (BMI)	rs1860018	A	G	17	13846723	0.443251	-0.00848468	0.00119697	2.49977e-13	180726
Sex hormone-binding globulin levels	Body mass index (BMI)	rs187079266	A	G	17	7438801	0.013934	-0.072588	0.00515995	7.70016e-47	180726
Sex hormone-b	Body mass	rs1871	G	A	12	21352315	0.151789	-0.032	0.00171209	4.6004	180726

inding globulin levels	index (BMI)	395						5026		5e-88	
Sex hormone-binding globulin levels	Body mass index (BMI)	rs187437	A	G	7	116445091	0.453037	-0.00826966	0.00123975	9.60064e-14	180726
Sex hormone-binding globulin levels	Body mass index (BMI)	rs2045345	G	A	1	26818894	0.194645	-0.0118503	0.00158288	1e-13	180726
Sex hormone-binding globulin levels	Body mass index (BMI)	rs2073503	G	C	4	3536176	0.310531	0.00683324	0.00132685	2.1e-08	180726
Sex hormone-binding globulin levels	Body mass index (BMI)	rs2123050	T	A	15	35224247	0.60582	-0.00886187	0.00126102	1.8001e-13	180726
Sex hormone-binding globulin levels	Body mass index (BMI)	rs2138161	C	T	2	227095159	0.64815	-0.0130685	0.00128154	1.39991e-24	180726
Sex hormone-binding globulin levels	Body mass index (BMI)	rs2239222	G	A	14	73011885	0.349619	0.0110595	0.00130116	5.50047e-17	180726
Sex hormone-binding globulin levels	Body mass index (BMI)	rs2256657	G	A	10	65712613	0.853507	-0.0140088	0.00173169	6.29941e-18	180726
Sex hormone-binding globulin levels	Body mass index (BMI)	rs2259305	A	G	10	93615903	0.476995	-0.0118863	0.00122291	2.90001e-22	180726
Sex	Body	rs2	A	G	1	1710	0.4166	-0.	0.0012	4.0	180

hormone-binding globulin levels	mass index (BMI)	266782				76966	47	00783493	4201	9996e-10	726
Sex hormone-binding globulin levels	Body mass index (BMI)	rs2298058	T	C	13	95248566	0.306244	0.0111891	0.0013408	2.09991e-18	180726
Sex hormone-binding globulin levels	Body mass index (BMI)	rs2301050	T	C	3	105401507	0.275057	-0.00678906	0.00137554	4.30002e-08	180726
Sex hormone-binding globulin levels	Body mass index (BMI)	rs2330649	A	G	22	24295053	0.566063	-0.0100381	0.00126705	4.00037e-17	180726
Sex hormone-binding globulin levels	Body mass index (BMI)	rs2351958	A	C	11	16248020	0.387523	-0.0101948	0.00126369	1.29987e-17	180726
Sex hormone-binding globulin levels	Body mass index (BMI)	rs2575368	G	T	16	1817397	0.919707	-0.0120002	0.00227266	3.09999e-08	180726
Sex hormone-binding globulin levels	Body mass index (BMI)	rs2612069	G	C	12	66215292	0.096031	-0.0120066	0.0020917	8.10009e-09	180726
Sex hormone-binding globulin levels	Body mass index (BMI)	rs2642438	G	A	1	220970028	0.701828	0.0150028	0.00133645	3.40017e-31	180726
Sex hormone-binding globulin levels	Body mass index (BMI)	rs2694157	C	T	7	101907808	0.620654	0.00902024	0.00126663	7.89951e-14	180726

Sex hormone-binding globulin levels	Body mass index (BMI)	rs2721195	C	T	8	145677011	0.52513	-0.0112875	0.001238	5.30029e-21	180726
Sex hormone-binding globulin levels	Body mass index (BMI)	rs28773027	A	G	4	88076227	0.101446	0.0119206	0.00205087	1.89998e-10	180726
Sex hormone-binding globulin levels	Body mass index (BMI)	rs2896906	C	T	16	4647417	0.193587	-0.00970772	0.00156221	1.09999e-10	180726
Sex hormone-binding globulin levels	Body mass index (BMI)	rs2905801	T	C	17	29524974	0.705121	0.0140121	0.00130719	3.80014e-28	180726
Sex hormone-binding globulin levels	Body mass index (BMI)	rs2924545	A	G	11	68883281	0.429092	-0.00664564	0.00124894	5e-09	180726
Sex hormone-binding globulin levels	Body mass index (BMI)	rs3006593	C	G	10	31171626	0.379929	0.00767249	0.00125767	7.90005e-10	180726
Sex hormone-binding globulin levels	Body mass index (BMI)	rs3173833	G	T	7	150491084	0.573379	0.00768356	0.0012443	6.80002e-09	180726
Sex hormone-binding globulin levels	Body mass index (BMI)	rs34255979	T	C	19	46384830	0.119463	0.0272468	0.00190546	4.0003e-51	180726
Sex hormone-binding globulin	Body mass index (BMI)	rs34587839	A	G	20	32300671	0.155551	-0.0125669	0.00170732	1.10002e-14	180726

levels											
Sex hormone-binding globulin levels	Body mass index (BMI)	rs34869253	C	T	4	1009420	0.428735	-0.00809933	0.00125064	3.59998e-12	180726
Sex hormone-binding globulin levels	Body mass index (BMI)	rs35135293	T	C	2	20363666	0.519066	-0.00737332	0.00122906	7.29962e-12	180726
Sex hormone-binding globulin levels	Body mass index (BMI)	rs35234337	T	C	9	35661243	0.257471	-0.00760759	0.00141397	2.30001e-08	180726
Sex hormone-binding globulin levels	Body mass index (BMI)	rs35346083	A	C	1	25788425	0.443854	-0.0104562	0.00123226	8.00018e-19	180726
Sex hormone-binding globulin levels	Body mass index (BMI)	rs3730393	A	G	16	67226405	0.040398	-0.0188678	0.00316246	2.1e-10	180726
Sex hormone-binding globulin levels	Body mass index (BMI)	rs3795128	C	T	20	39774163	0.483632	0.00977417	0.00123946	1.50003e-17	180726
Sex hormone-binding globulin levels	Body mass index (BMI)	rs3890483	T	G	19	7220596	0.442264	-0.0111819	0.00124431	1.99986e-21	180726
Sex hormone-binding globulin levels	Body mass index (BMI)	rs3891167	G	A	18	658423	0.257163	0.00815923	0.0014729	3.59998e-08	180726
Sex hormone-binding	Body mass index	rs40270	C	A	5	55804552	0.772121	-0.014166	0.00146708	1.29987e-	180726

globulin levels	(BMI)							5		22	
Sex hormone-binding globulin levels	Body mass index (BMI)	rs4092465	G	A	18	55080437	0.647016	0.00857154	0.00130236	3.59998e-13	180726
Sex hormone-binding globulin levels	Body mass index (BMI)	rs42945	G	A	16	58545426	0.51158	-0.00881296	0.00124194	3.19963e-13	180726
Sex hormone-binding globulin levels	Body mass index (BMI)	rs4297769	A	G	17	7944048	0.389563	-0.0138222	0.00122432	1.9002e-28	180726
Sex hormone-binding globulin levels	Body mass index (BMI)	rs4327534	A	C	4	157627879	0.319889	0.0070071	0.00131672	3.79997e-08	180726
Sex hormone-binding globulin levels	Body mass index (BMI)	rs4671607	C	T	2	64905898	0.737605	-0.0156863	0.00140192	2.90001e-33	180726
Sex hormone-binding globulin levels	Body mass index (BMI)	rs4675682	C	T	2	208402750	0.46019	-0.00894896	0.00123031	3.10027e-15	180726
Sex hormone-binding globulin levels	Body mass index (BMI)	rs4715316	T	C	6	52628998	0.644472	0.00866613	0.00129252	1.99986e-13	180726
Sex hormone-binding globulin levels	Body mass index (BMI)	rs4719366	A	G	7	1977906	0.397059	-0.00801322	0.00126634	2.19989e-11	180726
Sex hormone-b	Body mass	rs4819	A	C	22	20136379	0.297211	0.0068	0.00135593	5e-09	180726

inding globulin levels	index (BMI)	867						8975			
Sex hormone-binding globulin levels	Body mass index (BMI)	rs4841133	G	A	8	9183664	0.908681	0.022133	0.00214218	1.39991e-28	180726
Sex hormone-binding globulin levels	Body mass index (BMI)	rs4918722	T	C	10	113947040	0.727446	-0.00951318	0.00137242	5.10035e-14	180726
Sex hormone-binding globulin levels	Body mass index (BMI)	rs540730	C	T	12	57807114	0.754418	-0.0177235	0.00142693	4.90004e-39	180726
Sex hormone-binding globulin levels	Body mass index (BMI)	rs55771168	C	T	11	119070949	0.271108	0.00745775	0.00138631	1.2e-08	180726
Sex hormone-binding globulin levels	Body mass index (BMI)	rs55869502	T	C	14	100825670	0.4951	-0.0113108	0.00123479	3.29989e-22	180726
Sex hormone-binding globulin levels	Body mass index (BMI)	rs55987409	T	C	20	49569025	0.072538	0.0141619	0.00238657	1.29999e-09	180726
Sex hormone-binding globulin levels	Body mass index (BMI)	rs56196860	A	C	12	2908330	0.031512	0.0219327	0.00351758	6.59933e-11	180726
Sex hormone-binding globulin levels	Body mass index (BMI)	rs56332871	A	C	15	96714816	0.272043	0.0298253	0.00139111	4.19759e-111	180726
Sex	Body	rs5	T	A	8	8142	0.1581	0.0	0.0016	8.6	180

hormone-binding globulin levels	mass index (BMI)	9203582				4720	08	17504	9193	9961e-30	726
Sex hormone-binding globulin levels	Body mass index (BMI)	rs6130613	T	C	20	43054441	0.469585	0.017418	0.00125777	7.50067e-45	180726
Sex hormone-binding globulin levels	Body mass index (BMI)	rs62472728	T	C	7	143105566	0.060894	0.0170313	0.00260051	9.59997e-10	180726
Sex hormone-binding globulin levels	Body mass index (BMI)	rs62618693	T	C	11	32956492	0.045536	0.0187921	0.00294867	5.19996e-11	180726
Sex hormone-binding globulin levels	Body mass index (BMI)	rs631695	G	T	11	69283303	0.582362	-0.0168256	0.00124634	8.69961e-47	180726
Sex hormone-binding globulin levels	Body mass index (BMI)	rs6480299	G	C	10	69631393	0.669646	-0.0109315	0.00130057	3.80014e-17	180726
Sex hormone-binding globulin levels	Body mass index (BMI)	rs6532796	G	A	4	100042242	0.706404	-0.0200993	0.00134644	4.70002e-51	180726
Sex hormone-binding globulin levels	Body mass index (BMI)	rs6541725	A	C	2	121338032	0.379609	-0.00773737	0.0012627	1.79999e-09	180726
Sex hormone-binding globulin levels	Body mass index (BMI)	rs6736913	G	A	2	42510018	0.978716	-0.0329121	0.00423623	2.99985e-15	180726

Sex hormone-binding globulin levels	Body mass index (BMI)	rs6758199	T	C	2	70537173	0.066765	-0.016905	0.00246789	3.50026e-12	180726
Sex hormone-binding globulin levels	Body mass index (BMI)	rs6939861	A	G	6	41703041	0.261847	-0.0112806	0.00142004	1.10002e-15	180726
Sex hormone-binding globulin levels	Body mass index (BMI)	rs6950023	G	T	7	97915635	0.8144	0.0316741	0.00157952	9.8992e-96	180726
Sex hormone-binding globulin levels	Body mass index (BMI)	rs7092853	T	C	10	64429307	0.850678	-0.0108014	0.00171373	6.79986e-11	180726
Sex hormone-binding globulin levels	Body mass index (BMI)	rs720130	T	G	16	11132633	0.404437	-0.00659278	0.00125987	7.79992e-09	180726
Sex hormone-binding globulin levels	Body mass index (BMI)	rs7225349	A	G	17	73240009	0.832522	-0.0133437	0.00160389	5.19996e-17	180726
Sex hormone-binding globulin levels	Body mass index (BMI)	rs72787581	A	G	2	33012808	0.023397	0.0246186	0.00409905	7.10003e-10	180726
Sex hormone-binding globulin levels	Body mass index (BMI)	rs72842811	T	C	17	7296899	0.033389	-0.0653578	0.00357483	3.40017e-77	180726
Sex hormone-binding globulin	Body mass index (BMI)	rs72929847	A	G	1	61684630	0.076546	0.0196337	0.00230744	6.20012e-18	180726

levels											
Sex hormone-binding globulin levels	Body mass index (BMI)	rs7314285	G	T	12	111522026	0.068474	0.0301165	0.00243544	1.50003e-41	180726
Sex hormone-binding globulin levels	Body mass index (BMI)	rs74551598	C	A	9	117177566	0.243984	-0.00836883	0.00143759	1e-09	180726
Sex hormone-binding globulin levels	Body mass index (BMI)	rs75130744	C	G	12	25410741	0.071554	-0.0296028	0.00238856	3.10027e-39	180726
Sex hormone-binding globulin levels	Body mass index (BMI)	rs7540115	C	A	1	200265618	0.818722	0.00936579	0.0015874	1.09999e-09	180726
Sex hormone-binding globulin levels	Body mass index (BMI)	rs77483564	T	C	4	111096883	0.016978	0.0303068	0.00485249	3.69999e-10	180726
Sex hormone-binding globulin levels	Body mass index (BMI)	rs776746	T	C	7	99270539	0.067312	0.0152833	0.00245492	6.09958e-11	180726
Sex hormone-binding globulin levels	Body mass index (BMI)	rs7773423	G	A	6	116363149	0.40114	0.00749373	0.00125965	2.1e-09	180726
Sex hormone-binding globulin levels	Body mass index (BMI)	rs7860634	A	G	9	139089679	0.573824	0.0123675	0.00124597	6.09958e-25	180726
Sex hormone-binding	Body mass index	rs7867931	G	T	9	113033907	0.187187	0.0089348	0.0015856	2.39999e-	180726

globulin levels	(BMI)	8						4		09	
Sex hormone-binding globulin levels	Body mass index (BMI)	rs78890745	A	G	4	159834474	0.108878	0.0136529	0.00198065	8.9002e-12	180726
Sex hormone-binding globulin levels	Body mass index (BMI)	rs7920217	T	C	10	105668172	0.504413	0.00773882	0.00122079	1.09999e-09	180726
Sex hormone-binding globulin levels	Body mass index (BMI)	rs79717793	A	G	10	5262267	0.155045	-0.0246105	0.00168938	2.60016e-55	180726
Sex hormone-binding globulin levels	Body mass index (BMI)	rs79760705	T	G	5	53298716	0.113023	-0.0175959	0.00194631	1.99986e-21	180726
Sex hormone-binding globulin levels	Body mass index (BMI)	rs820503	A	C	9	6667928	0.137437	-0.0112088	0.0017888	3.69999e-11	180726
Sex hormone-binding globulin levels	Body mass index (BMI)	rs876435	A	G	8	22873533	0.590337	-0.00756735	0.00126472	2.99985e-11	180726
Sex hormone-binding globulin levels	Body mass index (BMI)	rs9288177	A	G	2	191585252	0.374299	-0.00898949	0.00127263	5.00035e-13	180726
Sex hormone-binding globulin levels	Body mass index (BMI)	rs9379084	A	G	6	7231843	0.115346	-0.0153264	0.00198813	1e-15	180726
Sex hormone-b	Body mass	rs9492	C	A	6	32610976	0.182992	-0.009	0.00176134	3.7999	180726

inding<br>globulin<br>levels	index<br>(BMI)							475<br>56		7e-<br>09	
Sex<br>hormone-b<br>inding<br>globulin<br>levels	Body<br>mass<br>index<br>(BMI)	rs9<br>697<br>210	A	G	9	1314<br>6874<br>0	0.1452<br>55	-0.<br>018<br>744<br>1	0.0017<br>5123	1.1<br>000<br>2e-<br>29	180<br>726
Sex<br>hormone-b<br>inding<br>globulin<br>levels	Body<br>mass<br>index<br>(BMI)	rs9<br>739<br>640	G	A	12	5378<br>3174	0.1679<br>95	-0.<br>009<br>481<br>38	0.0016<br>6929	1.2<br>999<br>9e-<br>08	180<br>726

Supplementary Table S7. Instruments for causal estimation from SHBG to BioT level.

Exposure	Outcome	SNP	Effect allele	Other allele	Chromosome	Genetic position	Effect allele frequency	Beta	Standard error of beta	Pvalue	Sample size
Sex hormone-binding globulin levels	Bioavailable testosterone levels	rs10027275	C	G	4	148981496	0.74126	-0.0125534	0.00141198	1.69981e-21	180726
Sex hormone-binding globulin levels	Bioavailable testosterone levels	rs10041660	C	T	5	122650224	0.50476	0.00755274	0.00124541	8.19993e-10	180726
Sex hormone-binding globulin levels	Bioavailable testosterone levels	rs10107182	T	C	8	59392737	0.663202	0.0124022	0.00130257	2.80027e-22	180726
Sex hormone-binding globulin levels	Bioavailable testosterone levels	rs1037169	C	T	11	13361005	0.687205	-0.0115299	0.00133154	3.69999e-20	180726
Sex hormone-binding globulin levels	Bioavailable testosterone levels	rs10733789	C	T	10	64948684	0.313039	0.0492531	0.00131963	1e-200	180726
Sex hormone-binding globulin levels	Bioavailable testosterone levels	rs10737683	A	G	1	196894829	0.236779	-0.010124	0.00144072	4.30031e-12	180726
Sex hormone-binding globulin levels	Bioavailable testosterone levels	rs10868080	A	T	9	86626769	0.744477	-0.0213279	0.00140815	1.50003e-57	180726
Sex hormone-b	Bioavailable	rs1087	G	A	18	57851763	0.236131	-0.009	0.00145802	5.0003	1807

inding	testoster	177						840		5e-	26
globulin	one	7						77		11	
levels	levels										
Sex	Bioavail										
hormone-b	able	rs1								9.3	
inding	testoster	088				1372		0.0		003	18
globulin	one	158				5607	0.2380	133	0.0014	7e-	07
levels	levels	2	A	G	9	8	37	501	5058	21	26
Sex	Bioavail										
hormone-b	able	rs1						-0.		8.9	
inding	testoster	089				1020		009		002	18
globulin	one	527				8494	0.3408	615	0.0013	e-1	07
levels	levels	7	G	A	11	0	06	47	0251	5	26
Sex	Bioavail										
hormone-b	able	rs1						0.0		8.0	
inding	testoster	107						092		001	18
globulin	one	525				1514	0.2960	234	0.0013	8e-	07
levels	levels	3	A	C	16	8646	81	5	5476	12	26
Sex	Bioavail										
hormone-b	able	rs1						-0.		4.6	
inding	testoster	111				1028		007		000	18
globulin	one	127				3812	0.7372	310	0.0013	2e-	07
levels	levels	4	A	G	12	8	79	72	8631	08	26
Sex	Bioavail										
hormone-b	able	rs1						0.0		2.1	
inding	testoster	115						078		999	18
globulin	one	897				7323	0.2877	724	0.0013	9e-	07
levels	levels	6	T	C	14	0278	21	2	6551	08	26
Sex	Bioavail										
hormone-b	able	rs1								3.1	
inding	testoster	115						-0.		002	18
globulin	one	934				2594	0.6808	010	0.0013	7e-	07
levels	levels	7	T	C	14	7436	71	709	2486	17	26
Sex	Bioavail										
hormone-b	able	rs1								2.8	
inding	testoster	117				1709		0.0		002	18
globulin	one	521				9489	0.0964	127	0.0020	7e-	07
levels	levels	58	T	C	4	4	96	725	799	11	26
Sex	Bioavail										
hormone-b	able	rs1								8.1	
inding	testoster	119						0.0		997	18
globulin	one	812				5001	0.0789	261	0.0022	4e-	07
levels	levels	33	G	T	19	6479	84	638	7727	33	26
Sex	Bioavail	rs1	T	C	13	1150	0.2318	-0.	0.0014	2.3	18

hormone-b<br>inding<br>globulin<br>levels	able<br>testoster<br>one<br>levels	120<br>359<br>22				4746<br>4	57	008<br>812	6895	000<br>1e-<br>10	07<br>26
Sex<br>hormone-b<br>inding<br>globulin<br>levels	Bioavail<br>able<br>testoster<br>one<br>levels	rs1<br>132<br>484<br>43	G	A	10	6610<br>2542	0.0482<br>94	-0.<br>014<br>367<br>4	0.0029<br>4711	2.9<br>000<br>1e-<br>08	18<br>07<br>26
Sex<br>hormone-b<br>inding<br>globulin<br>levels	Bioavail<br>able<br>testoster<br>one<br>levels	rs1<br>141<br>653<br>49	C	G	1	2702<br>1913	0.0231<br>58	-0.<br>087<br>113<br>6	0.0040<br>8954	3.4<br>994<br>5e-<br>111	18<br>07<br>26
Sex<br>hormone-b<br>inding<br>globulin<br>levels	Bioavail<br>able<br>testoster<br>one<br>levels	rs1<br>149<br>492<br>63	C	T	7	1504<br>9824<br>5	0.1113<br>88	0.0<br>187<br>991	0.0019<br>6067	4.1<br>995<br>2e-<br>25	18<br>07<br>26
Sex<br>hormone-b<br>inding<br>globulin<br>levels	Bioavail<br>able<br>testoster<br>one<br>levels	rs1<br>152<br>766<br>19	A	T	1	1848<br>6513<br>2	0.0166<br>26	-0.<br>028<br>539<br>9	0.0049<br>3428	3.6<br>999<br>9e-<br>11	18<br>07<br>26
Sex<br>hormone-b<br>inding<br>globulin<br>levels	Bioavail<br>able<br>testoster<br>one<br>levels	rs1<br>154<br>988	A	T	3	1359<br>2519<br>1	0.7730<br>31	-0.<br>027<br>658<br>7	0.0014<br>6575	4.2<br>004<br>9e-<br>88	18<br>07<br>26
Sex<br>hormone-b<br>inding<br>globulin<br>levels	Bioavail<br>able<br>testoster<br>one<br>levels	rs1<br>156<br>472<br>2	T	C	11	2178<br>330	0.2387<br>4	0.0<br>095<br>530<br>7	0.0014<br>6515	1.2<br>000<br>5e-<br>13	18<br>07<br>26
Sex<br>hormone-b<br>inding<br>globulin<br>levels	Bioavail<br>able<br>testoster<br>one<br>levels	rs1<br>161<br>025<br>6	T	C	12	1117<br>3412<br>1	0.2166<br>65	0.0<br>086<br>783<br>8	0.0014<br>9523	2.1<br>e-0<br>9	18<br>07<br>26
Sex<br>hormone-b<br>inding<br>globulin<br>levels	Bioavail<br>able<br>testoster<br>one<br>levels	rs1<br>162<br>179<br>2	T	C	14	2487<br>1926	0.4536<br>47	-0.<br>012<br>749	0.0012<br>4244	1.3<br>999<br>1e-<br>25	18<br>07<br>26

Sex hormone-binding globulin levels	Bioavailable testosterone levels	rs116338429	T	C	13	114767040	0.170752	0.00985678	0.00172794	1.29999e-09	180726
Sex hormone-binding globulin levels	Bioavailable testosterone levels	rs1666245	A	G	19	38229926	0.047306	-0.0163325	0.0029014	2.19999e-09	180726
Sex hormone-binding globulin levels	Bioavailable testosterone levels	rs1734408	G	A	4	23882519	0.293256	0.00767697	0.00134935	3.29997e-10	180726
Sex hormone-binding globulin levels	Bioavailable testosterone levels	rs1739158	T	C	5	72927292	0.427811	0.00829379	0.00124625	2.09991e-12	180726
Sex hormone-binding globulin levels	Bioavailable testosterone levels	rs1743810	T	C	5	137802404	0.560069	0.00893646	0.00124066	1.99986e-12	180726
Sex hormone-binding globulin levels	Bioavailable testosterone levels	rs17664177	T	C	12	101036562	0.01211	-0.051561	0.00573803	3.69999e-22	180726
Sex hormone-binding globulin levels	Bioavailable testosterone levels	rs17734486	T	C	8	81061523	0.061081	-0.0133401	0.00261459	1.2e-08	180726
Sex hormone-binding globulin levels	Bioavailable testosterone levels	rs17921873	G	A	8	81473835	0.066515	0.021022	0.00250814	4.70002e-18	180726
Sex hormone-binding globulin	Bioavailable testosterone	rs18037027	T	A	12	21203424	0.040459	0.0222618	0.00313784	3.69999e-13	180726

levels	levels										
Sex hormone-binding globulin levels	Bioavailable testosterone levels	rs11856926	A	G	15	96223649	0.448829	-0.011587	0.00124664	1.9002e-22	180726
Sex hormone-binding globulin levels	Bioavailable testosterone levels	rs11870307	G	A	17	7617787	0.780506	0.0526994	0.00145305	1e-200	180726
Sex hormone-binding globulin levels	Bioavailable testosterone levels	rs12138136	A	T	1	149844305	0.086596	0.0152389	0.00220676	6.29941e-14	180726
Sex hormone-binding globulin levels	Bioavailable testosterone levels	rs1229492	C	T	7	81564122	0.731518	-0.0114669	0.00139655	6.29941e-18	180726
Sex hormone-binding globulin levels	Bioavailable testosterone levels	rs12543287	C	G	8	42334511	0.370844	0.0106061	0.00128263	1.9002e-18	180726
Sex hormone-binding globulin levels	Bioavailable testosterone levels	rs1260326	C	T	2	27730940	0.605679	0.0380831	0.00125216	1e-200	180726
Sex hormone-binding globulin levels	Bioavailable testosterone levels	rs12605524	G	A	18	22073551	0.28752	-0.00764797	0.00137562	1.2e-08	180726
Sex hormone-binding globulin levels	Bioavailable testosterone levels	rs12611046	A	G	19	14291704	0.150636	-0.00968	0.00177915	7.00003e-09	180726
Sex hormone-binding	Bioavailable testoster	rs1269445	C	T	2	220019638	0.675226	-0.007064	0.0013572	3.79997e-	180726

globulin	one	0						09		08	
levels	levels										
Sex	Bioavail										
hormone-b	able	rs1						0.0			
inding	testoster	269				1694		081		1.7	18
globulin	one	630				8127	0.2656	310	0.0013	e-0	07
levels	levels	4	G	C	3	1	45	9	9236	9	26
Sex	Bioavail										
hormone-b	able	rs1								6.0	
inding	testoster	279						0.0		995	18
globulin	one	770				6556	0.2345	120	0.0014	8e-	07
levels	levels	6	A	G	11	1369	88	832	6721	17	26
Sex	Bioavail										
hormone-b	able	rs1								7.3	
inding	testoster	295						0.0		994	18
globulin	one	056				1799	0.6139	110	0.0012	6e-	07
levels	levels	2	T	C	17	5166	66	236	2815	21	26
Sex	Bioavail										
hormone-b	able	rs1						0.0		7.4	
inding	testoster	295						082		999	18
globulin	one	308				5926	0.7950	792	0.0014	8e-	07
levels	levels	7	T	C	17	5201	92	4	8126	09	26
Sex	Bioavail										
hormone-b	able	rs1						0.0		9.5	
inding	testoster	298				1485		079		999	18
globulin	one	959				9123	0.2916	174	0.0013	7e-	07
levels	levels	6	T	G	2	2	51	7	5721	10	26
Sex	Bioavail										
hormone-b	able	rs1						-0.		1.2	
inding	testoster	310						022		000	18
globulin	one	821				3443	0.6172	994	0.0012	5e-	07
levels	levels	8	G	A	4	931	54	3	7338	79	26
Sex	Bioavail										
hormone-b	able	rs1						-0.		7.5	
inding	testoster	315						020		997	18
globulin	one	006				8820	0.4376	732	0.0012	6e-	07
levels	levels	8	G	A	4	3828	37	9	3764	67	26
Sex	Bioavail										
hormone-b	able	rs1						0.0		2.1	
inding	testoster	337						082		999	18
globulin	one	904				7425	0.2800	885	0.0013	9e-	07
levels	levels	3	C	T	14	0126	09	2	9928	10	26
Sex	Bioavail	rs1				5622	0.0768	-0.	0.0023	3.5	18
hormone-b	able	386	C	T	5	8040	23	014	084	002	07

inding globulin levels	testosterone levels	11541						8471		6e-12	26
Sex hormone-binding globulin levels	Bioavailable testosterone levels	rs138820	C	T	22	50164078	0.215284	-0.00803676	0.00150694	2e-08	180726
Sex hormone-binding globulin levels	Bioavailable testosterone levels	rs1415293	T	A	1	219730006	0.318282	0.00743431	0.00131195	2.69998e-09	180726
Sex hormone-binding globulin levels	Bioavailable testosterone levels	rs144647926	A	G	1	23546767	0.087356	0.0123175	0.00217179	1.40001e-09	180726
Sex hormone-binding globulin levels	Bioavailable testosterone levels	rs149131600	T	C	19	35549122	0.17438	0.0205782	0.00162554	9.20026e-42	180726
Sex hormone-binding globulin levels	Bioavailable testosterone levels	rs150844304	C	A	15	43726625	0.024905	-0.0745127	0.0039585	9.3994e-85	180726
Sex hormone-binding globulin levels	Bioavailable testosterone levels	rs1538648	G	A	10	94820860	0.503197	-0.00757485	0.00122235	8.4004e-13	180726
Sex hormone-binding globulin levels	Bioavailable testosterone levels	rs157935	G	T	7	130585553	0.303601	0.0121026	0.00134199	1.50003e-22	180726
Sex hormone-binding globulin levels	Bioavailable testosterone levels	rs1730865	T	G	1	107605611	0.654768	-0.0260475	0.00129437	4.60045e-98	180726
Sex	Bioavail	rs1	A	G	19	2796	0.2872	0.0	0.0013	2.2	18

hormone-binding globulin levels	able testosterone levels	736180				021	61	173849	6123	9985e-41	0726
Sex hormone-binding globulin levels	Bioavailable testosterone levels	rs174529	C	T	11	61543961	0.375889	-0.00774839	0.00127239	4.20001e-10	180726
Sex hormone-binding globulin levels	Bioavailable testosterone levels	rs17580	A	T	14	94847262	0.048463	0.0304227	0.00286953	4.19952e-29	180726
Sex hormone-binding globulin levels	Bioavailable testosterone levels	rs1776899	A	G	6	34193230	0.969325	-0.0226948	0.0035717	5.69994e-10	180726
Sex hormone-binding globulin levels	Bioavailable testosterone levels	rs1782652	A	T	10	81074125	0.380625	-0.0127414	0.00127529	5.79963e-27	180726
Sex hormone-binding globulin levels	Bioavailable testosterone levels	rs1801689	C	A	17	64210580	0.029871	-0.03343	0.00350564	1.80011e-21	180726
Sex hormone-binding globulin levels	Bioavailable testosterone levels	rs1860018	A	G	17	13846723	0.443251	-0.00848468	0.00119697	2.49977e-13	180726
Sex hormone-binding globulin levels	Bioavailable testosterone levels	rs187079266	A	G	17	7438801	0.013934	-0.0725588	0.00515995	7.70016e-47	180726
Sex hormone-binding globulin levels	Bioavailable testosterone levels	rs1871395	G	A	12	21352315	0.151789	-0.0325026	0.00171209	4.60045e-88	180726

Sex hormone-binding globulin levels	Bioavailable testosterone levels	rs187437	A	G	7	116445091	0.453037	-0.00826966	0.00123975	9.60064e-14	180726
Sex hormone-binding globulin levels	Bioavailable testosterone levels	rs202200760	C	G	19	17346854	0.038592	0.0711034	0.00348775	3.69999e-98	180726
Sex hormone-binding globulin levels	Bioavailable testosterone levels	rs2045345	G	A	1	26818894	0.194645	-0.0118503	0.00158288	1e-13	180726
Sex hormone-binding globulin levels	Bioavailable testosterone levels	rs2073503	G	C	4	3536176	0.310531	0.00683324	0.00132685	2.1e-08	180726
Sex hormone-binding globulin levels	Bioavailable testosterone levels	rs2239222	G	A	14	73011885	0.349619	0.0110595	0.00130116	5.50047e-17	180726
Sex hormone-binding globulin levels	Bioavailable testosterone levels	rs2256657	G	A	10	65712613	0.853507	-0.0140088	0.00173169	6.29941e-18	180726
Sex hormone-binding globulin levels	Bioavailable testosterone levels	rs2259305	A	G	10	93615903	0.476995	-0.0118863	0.00122291	2.90001e-22	180726
Sex hormone-binding globulin levels	Bioavailable testosterone levels	rs2266782	A	G	1	171076966	0.416647	-0.00783493	0.00124201	4.09996e-10	180726
Sex hormone-binding globulin	Bioavailable testosterone	rs2298058	T	C	13	95248566	0.306244	0.0111891	0.0013408	2.09991e-18	180726

levels	levels										
Sex hormone-binding globulin levels	Bioavailable testosterone levels	rs2301050	T	C	3	105401507	0.275057	-0.00678906	0.00137554	4.30002e-08	180726
Sex hormone-binding globulin levels	Bioavailable testosterone levels	rs2330649	A	G	22	24295053	0.566063	-0.0100381	0.00126705	4.00037e-17	180726
Sex hormone-binding globulin levels	Bioavailable testosterone levels	rs2351958	A	C	11	16248020	0.387523	-0.0101948	0.00126369	1.29987e-17	180726
Sex hormone-binding globulin levels	Bioavailable testosterone levels	rs2575368	G	T	16	1817397	0.919707	-0.0120002	0.00227266	3.09999e-08	180726
Sex hormone-binding globulin levels	Bioavailable testosterone levels	rs2612069	G	C	12	66215292	0.096031	-0.0120066	0.0020917	8.10009e-09	180726
Sex hormone-binding globulin levels	Bioavailable testosterone levels	rs2642438	G	A	1	220970028	0.701828	0.0150028	0.00133645	3.40017e-31	180726
Sex hormone-binding globulin levels	Bioavailable testosterone levels	rs2694157	C	T	7	101907808	0.620654	0.00902024	0.00126663	7.89951e-14	180726
Sex hormone-binding globulin levels	Bioavailable testosterone levels	rs2721195	C	T	8	145677011	0.52513	-0.0112875	0.0012638	5.30029e-21	180726
Sex hormone-binding	Bioavailable testoster	rs2839486	A	G	17	47450775	0.460308	-0.029518	0.00120382	2.39883e-	180726

globulin levels	one levels	4								138	
Sex hormone-binding globulin levels	Bioavailable testosterone levels	rs28507491	A	G	4	77197651	0.375706	0.0139975	0.0012668	5.70033e-29	180726
Sex hormone-binding globulin levels	Bioavailable testosterone levels	rs28549287	A	G	1	110230138	0.774148	-0.0130821	0.00158912	4.90004e-17	180726
Sex hormone-binding globulin levels	Bioavailable testosterone levels	rs28601761	G	C	8	126500031	0.418125	-0.00644119	0.00126207	1.5e-08	180726
Sex hormone-binding globulin levels	Bioavailable testosterone levels	rs2862954	C	T	10	101912064	0.500343	0.0108541	0.00121838	3.40017e-19	180726
Sex hormone-binding globulin levels	Bioavailable testosterone levels	rs2872881	A	G	20	62342801	0.245662	0.00928822	0.00143199	4.79954e-11	180726
Sex hormone-binding globulin levels	Bioavailable testosterone levels	rs28773027	A	G	4	88076227	0.101446	0.0119206	0.00205087	1.89998e-10	180726
Sex hormone-binding globulin levels	Bioavailable testosterone levels	rs28929474	T	C	14	94844947	0.019823	0.138303	0.00441884	1e-200	180726
Sex hormone-binding globulin levels	Bioavailable testosterone levels	rs2896906	C	T	16	4647417	0.193587	-0.00970772	0.00156221	1.09999e-10	180726
Sex hormone-b	Bioavailable	rs2905	T	C	17	29524974	0.705121	0.0140	0.00130719	3.8001	1807

inding globulin levels	testosterone levels	801						121		4e-28	26
Sex hormone-binding globulin levels	Bioavailable testosterone levels	rs2914233	C	T	5	158013856	0.21093	-0.0085613	0.00151926	2.39999e-09	180726
Sex hormone-binding globulin levels	Bioavailable testosterone levels	rs2924545	A	G	11	68883281	0.429092	-0.00664564	0.00124894	5e-09	180726
Sex hormone-binding globulin levels	Bioavailable testosterone levels	rs2989330	G	A	21	40716209	0.340297	0.00743759	0.00131431	6.80002e-09	180726
Sex hormone-binding globulin levels	Bioavailable testosterone levels	rs3006593	C	G	10	31171626	0.379929	0.00767249	0.00125767	7.90005e-10	180726
Sex hormone-binding globulin levels	Bioavailable testosterone levels	rs3173833	G	T	7	150491084	0.573379	0.00768356	0.0012443	6.80002e-09	180726
Sex hormone-binding globulin levels	Bioavailable testosterone levels	rs34255979	T	C	19	46384830	0.119463	0.0272468	0.00190546	4.00037e-51	180726
Sex hormone-binding globulin levels	Bioavailable testosterone levels	rs34415062	A	G	19	59023166	0.179875	0.00876761	0.00160777	1.7e-08	180726
Sex hormone-binding globulin levels	Bioavailable testosterone levels	rs34587839	A	G	20	32300671	0.155551	-0.0125669	0.00170732	1.10002e-14	180726
Sex	Bioavail	rs3	G	C	19	1945	0.0786	-0.	0.0022	9.3	18

hormone-b	able	485				7235	55	015	9031	003	07
inding	testoster	858						798		7e-	26
globulin	one	8						7		15	
levels	levels										
Sex	Bioavail										
hormone-b	able	rs3						-0.		3.5	
inding	testoster	486						008		999	18
globulin	one	925				1009	0.4287	099	0.0012	8e-	07
levels	levels	3	C	T	4	420	35	33	5064	12	26
Sex	Bioavail										
hormone-b	able	rs3						-0.		7.2	
inding	testoster	513						007		996	18
globulin	one	529				2036	0.5190	373	0.0012	2e-	07
levels	levels	3	T	C	2	3666	66	32	2906	12	26
Sex	Bioavail										
hormone-b	able	rs3						-0.		2.3	
inding	testoster	523						007		000	18
globulin	one	433				3566	0.2574	607	0.0014	1e-	07
levels	levels	7	T	C	9	1243	71	59	1397	08	26
Sex	Bioavail										
hormone-b	able	rs3						-0.		8.0	
inding	testoster	534						010		001	18
globulin	one	608				2578	0.4438	456	0.0012	8e-	07
levels	levels	3	A	C	1	8425	54	2	3226	19	26
Sex	Bioavail										
hormone-b	able	rs3								4.4	
inding	testoster	608						0.0		998	18
globulin	one	619				1651	0.5789	167	0.0012	7e-	07
levels	levels	5	T	C	1	0894	71	429	3754	44	26
Sex	Bioavail										
hormone-b	able							-0.			
inding	testoster	rs3						018		2.1	18
globulin	one	730				6722	0.0403	867	0.0031	e-1	07
levels	levels	393	A	G	16	6405	98	8	6246	0	26
Sex	Bioavail										
hormone-b	able							-0.		1.2	
inding	testoster	rs3						015		000	18
globulin	one	768				4003	0.1971	519	0.0015	5e-	07
levels	levels	321	T	G	1	5928	78	9	4112	23	26
Sex	Bioavail										
hormone-b	able							0.0		1.5	
inding	testoster	rs3						097		000	18
globulin	one	795				3977	0.4836	741	0.0012	3e-	07
levels	levels	128	C	T	20	4163	32	7	3946	17	26

Sex	Bioavail										
hormone-b	able							-0.		1.9	
inding	testoster	rs3						011		998	18
globulin	one	890				7220	0.4422	181	0.0012	6e-	07
levels	levels	483	T	G	19	596	64	9	4431	21	26
Sex	Bioavail										
hormone-b	able							0.0		3.5	
inding	testoster	rs3						081		999	18
globulin	one	891				6584	0.2571	592	0.0014	8e-	07
levels	levels	167	G	A	18	23	63	3	729	08	26
Sex	Bioavail										
hormone-b	able							-0.		1.2	
inding	testoster	rs4						014		998	18
globulin	one	027				5580	0.7721	166	0.0014	7e-	07
levels	levels	0	C	A	5	4552	21	5	6708	22	26
Sex	Bioavail										
hormone-b	able							0.0		3.5	
inding	testoster	rs4						085		999	18
globulin	one	092				5508	0.6470	715	0.0013	8e-	07
levels	levels	465	G	A	18	0437	16	4	0236	13	26
Sex	Bioavail										
hormone-b	able							-0.		3.1	
inding	testoster	rs4						008		996	18
globulin	one	294				5854	0.5115	812	0.0012	3e-	07
levels	levels	5	G	A	16	5426	8	96	4194	13	26
Sex	Bioavail										
hormone-b	able							-0.		1.9	
inding	testoster	rs4						013		002	18
globulin	one	297				7944	0.3895	822	0.0012	e-2	07
levels	levels	769	A	G	17	048	63	2	432	8	26
Sex	Bioavail										
hormone-b	able									3.7	
inding	testoster	rs4				1576		0.0		999	18
globulin	one	327				2787	0.3198	070	0.0013	7e-	07
levels	levels	534	A	C	4	9	89	071	1672	08	26
Sex	Bioavail										
hormone-b	able							-0.		3.1	
inding	testoster	rs4				2084		008		002	18
globulin	one	675				0275	0.4601	948	0.0012	7e-	07
levels	levels	682	C	T	2	0	9	96	3031	15	26
Sex	Bioavail							-0.		2.1	
hormone-b	able	rs4						008		998	18
inding	testoster	719				1977	0.3970	013	0.0012	9e-	07
globulin	one	366	A	G	7	906	59	22	634	11	26

levels	levels										
Sex	Bioavail										
hormone-b	able							0.0			
inding	testoster	rs4						068			18
globulin	one	819				2013	0.2972	897	0.0013	5e-	07
levels	levels	867	A	C	22	6379	11	5	5593	09	26
Sex	Bioavail										
hormone-b	able									1.3	
inding	testoster	rs4						0.0		999	18
globulin	one	841				9183	0.9086	221	0.0021	1e-	07
levels	levels	133	G	A	8	664	81	33	4218	28	26
Sex	Bioavail										
hormone-b	able							-0.		5.1	
inding	testoster	rs4				1139		009		003	18
globulin	one	918				4704	0.7274	513	0.0013	5e-	07
levels	levels	722	T	C	10	0	46	18	7242	14	26
Sex	Bioavail										
hormone-b	able							-0.		4.9	
inding	testoster	rs5						017		000	18
globulin	one	407				5780	0.7544	723	0.0014	4e-	07
levels	levels	30	C	T	12	7114	18	5	2693	39	26
Sex	Bioavail										
hormone-b	able	rs5								3.1	
inding	testoster	572						0.0		996	18
globulin	one	943				8851	0.2652	104	0.0014	3e-	07
levels	levels	2	G	C	16	1548	78	733	0802	15	26
Sex	Bioavail										
hormone-b	able	rs5						-0.		3.2	
inding	testoster	586				1008		011		998	18
globulin	one	950				2567		310	0.0012	9e-	07
levels	levels	2	T	C	14	0	0.4951	8	3479	22	26
Sex	Bioavail										
hormone-b	able	rs5								1.2	
inding	testoster	598						0.0		999	18
globulin	one	740				4956	0.0725	141	0.0023	9e-	07
levels	levels	9	T	C	20	9025	38	619	8657	09	26
Sex	Bioavail										
hormone-b	able	rs5								2.8	
inding	testoster	607						0.0		000	18
globulin	one	734				4480	0.0712	136	0.0024	1e-	07
levels	levels	5	C	G	7	8650	31	246	0009	09	26
Sex	Bioavail	rs5						0.0		4.1	18
hormone-b	able	633				9671	0.2720	298	0.0013	975	07
inding	testoster	287	A	C	15	4816	43	253	9111	9e-	26

globulin	one	1								111	
levels	levels										
Sex	Bioavail										
hormone-b	able	rs5						-0.		8.7	
inding	testoster	715				1853		006		000	18
globulin	one	876				7117	0.4363	645	0.0012	1e-	07
levels	levels	1	G	A	3	2	7	62	4159	09	26
Sex	Bioavail										
hormone-b	able	rs5						0.0		2.5	
inding	testoster	841						075		999	18
globulin	one	455				3755	0.3057	129	0.0013	8e-	07
levels	levels	5	C	A	20	4152	98	3	4031	08	26
Sex	Bioavail										
hormone-b	able	rs5								8.6	
inding	testoster	920						0.0		996	18
globulin	one	358				8142	0.1581	175	0.0016	1e-	07
levels	levels	2	T	A	8	4720	08	04	9193	30	26
Sex	Bioavail										
hormone-b	able	rs6									
inding	testoster	001						0.0			18
globulin	one	814				3375	0.1210	131	0.0019	1e-	07
levels	levels	7	G	A	19	572	31	5	8109	12	26
Sex	Bioavail										
hormone-b	able							-0.		4.0	
inding	testoster	rs6						016		003	18
globulin	one	005				2910	0.6727	874	0.0013	7e-	07
levels	levels	840	G	A	22	1357	01	7	1567	39	26
Sex	Bioavail										
hormone-b	able									7.5	
inding	testoster	rs6						0.0		006	18
globulin	one	130				4305	0.4695	174	0.0012	7e-	07
levels	levels	613	T	C	20	4441	85	18	5777	45	26
Sex	Bioavail										
hormone-b	able	rs6								5.3	
inding	testoster	168						0.0		002	18
globulin	one	664				1508	0.2597	103	0.0014	9e-	07
levels	levels	4	G	A	1	819	71	828	7133	14	26
Sex	Bioavail										
hormone-b	able	rs6						-0.		1.1	
inding	testoster	201						012		000	18
globulin	one	128				6379	0.3424	613	0.0012	2e-	07
levels	levels	6	A	G	15	1125	96	6	9832	25	26
Sex	Bioavail	rs6				4938	0.4381	-0.	0.0012	2.0	18
hormone-b	able	225	A	G	3	6047	62	009	3926	999	07

inding globulin levels	testosterone levels	9939						10972		1e-13	26
Sex hormone-binding globulin levels	Bioavailable testosterone levels	rs624319	A	G	6	160772900	0.473485	0.0151961	0.00123751	5.70033e-40	180726
Sex hormone-binding globulin levels	Bioavailable testosterone levels	rs62472728	T	C	7	143105566	0.060894	0.0170313	0.00260051	9.59997e-10	180726
Sex hormone-binding globulin levels	Bioavailable testosterone levels	rs62618693	T	C	11	32956492	0.045536	0.0187921	0.00294867	5.19996e-11	180726
Sex hormone-binding globulin levels	Bioavailable testosterone levels	rs631695	G	T	11	69283303	0.582362	-0.0168256	0.00124634	8.69961e-47	180726
Sex hormone-binding globulin levels	Bioavailable testosterone levels	rs6422513	A	G	1	93032467	0.792896	0.0129507	0.00150077	3.80014e-18	180726
Sex hormone-binding globulin levels	Bioavailable testosterone levels	rs6480299	G	C	10	69631393	0.669646	-0.0109315	0.00130057	3.80014e-17	180726
Sex hormone-binding globulin levels	Bioavailable testosterone levels	rs6532796	G	A	4	100042242	0.706404	-0.0200993	0.00134644	4.70002e-51	180726
Sex hormone-binding globulin levels	Bioavailable testosterone levels	rs6541725	A	C	2	121338032	0.379609	-0.00773737	0.00120627	1.79999e-09	180726
Sex	Bioavail	rs6	C	T	2	1655	0.4029	0.0	0.0012	6.2	18

hormone-binding globulin levels	able testosterone levels	717858				39661	74	113005	4935	0012e-21	0726
Sex hormone-binding globulin levels	Bioavailable testosterone levels	rs6736913	G	A	2	42510018	0.978716	-0.0329121	0.00423623	2.99985e-15	180726
Sex hormone-binding globulin levels	Bioavailable testosterone levels	rs6758199	T	C	2	70537173	0.066765	-0.016905	0.00246789	3.50026e-12	180726
Sex hormone-binding globulin levels	Bioavailable testosterone levels	rs6792725	G	A	3	24520283	0.694054	0.0120526	0.0013744	2.09991e-21	180726
Sex hormone-binding globulin levels	Bioavailable testosterone levels	rs6900473	G	A	6	130375810	0.688161	-0.00890429	0.00133223	7.39946e-12	180726
Sex hormone-binding globulin levels	Bioavailable testosterone levels	rs6939861	A	G	6	41703041	0.261847	-0.0112806	0.00142004	1.10002e-15	180726
Sex hormone-binding globulin levels	Bioavailable testosterone levels	rs6950023	G	T	7	97915635	0.8144	0.0316741	0.00157952	9.8992e-96	180726
Sex hormone-binding globulin levels	Bioavailable testosterone levels	rs7085615	A	G	10	64768990	0.104062	0.0305713	0.00200364	9.70063e-59	180726
Sex hormone-binding globulin levels	Bioavailable testosterone levels	rs7092853	T	C	10	64429307	0.850678	-0.0108014	0.00171373	6.79986e-11	180726

Sex hormone-binding globulin levels	Bioavailable testosterone levels	rs7127472	T	C	11	61937658	0.214918	0.0089718	0.00150774	3.69999e-09	180726
Sex hormone-binding globulin levels	Bioavailable testosterone levels	rs71480542	A	C	15	59966306	0.382197	0.00688656	0.00130727	3.29997e-08	180726
Sex hormone-binding globulin levels	Bioavailable testosterone levels	rs7177179	C	T	15	40287982	0.348882	-0.00728003	0.00129819	3.89996e-08	180726
Sex hormone-binding globulin levels	Bioavailable testosterone levels	rs720130	T	G	16	11132633	0.404437	-0.00659278	0.00125987	7.79992e-09	180726
Sex hormone-binding globulin levels	Bioavailable testosterone levels	rs7225349	A	G	17	73240009	0.832522	-0.0133437	0.00160389	5.19996e-17	180726
Sex hormone-binding globulin levels	Bioavailable testosterone levels	rs72666817	A	G	4	104129141	0.204451	0.00922268	0.00151249	2.59998e-10	180726
Sex hormone-binding globulin levels	Bioavailable testosterone levels	rs72683923	C	T	14	50735947	0.019679	0.0339786	0.00443863	2.99985e-16	180726
Sex hormone-binding globulin levels	Bioavailable testosterone levels	rs72787581	A	G	2	33012808	0.023397	0.0246186	0.00409905	7.10003e-10	180726
Sex hormone-binding globulin	Bioavailable testosterone	rs72842811	T	C	17	7296899	0.033389	-0.0653578	0.00357483	3.40017e-77	180726

levels	levels										
Sex	Bioavail										
hormone-b	able	rs7								6.2	
inding	testoster	292						0.0		001	18
globulin	one	984				6168	0.0765	196	0.0023	2e-	07
levels	levels	7	A	G	1	4630	46	337	0744	18	26
Sex	Bioavail										
hormone-b	able	rs7						-0.		4.7	
inding	testoster	294				1781		012		000	18
globulin	one	811				6708	0.0948	284	0.0020	2e-	07
levels	levels	5	T	C	2	6	7	8	9101	09	26
Sex	Bioavail										
hormone-b	able									1.5	
inding	testoster	rs7				1115		0.0		000	18
globulin	one	314				2202	0.0684	301	0.0024	3e-	07
levels	levels	285	G	T	12	6	74	165	3544	41	26
Sex	Bioavail										
hormone-b	able							0.0		2.1	
inding	testoster	rs7						068		999	18
globulin	one	318				3389	0.6650	962	0.0013	9e-	07
levels	levels	39	A	G	19	9065	34	6	066	08	26
Sex	Bioavail										
hormone-b	able									1.8	
inding	testoster	rs7						0.0		001	18
globulin	one	384				4432	0.2162	309	0.0014	1e-	07
levels	levels	09	G	C	22	4727	44	602	9718	96	26
Sex	Bioavail										
hormone-b	able	rs7						-0.			
inding	testoster	455				1171		008			18
globulin	one	159				7756	0.2439	368	0.0014	1e-	07
levels	levels	8	C	A	9	6	84	83	3759	09	26
Sex	Bioavail										
hormone-b	able	rs7						-0.		3.1	
inding	testoster	513						029		002	18
globulin	one	074				2541	0.0715	602	0.0023	7e-	07
levels	levels	4	C	G	12	0741	54	8	8856	39	26
Sex	Bioavail										
hormone-b	able							0.0		1.0	
inding	testoster	rs7				2002		093		999	18
globulin	one	540				6561	0.8187	657	0.0015	9e-	07
levels	levels	115	C	A	1	8	22	9	874	09	26
Sex	Bioavail	rs7						-0.		2.1	18
hormone-b	able	563				1804	0.2169	008	0.0015	999	07
inding	testoster	164	C	T	4	9216	19	497	2246	9e-	26

globulin levels	one levels	2						96		08	
Sex hormone-binding globulin levels	Bioavailable testosterone levels	rs77483564	T	C	4	111096883	0.016978	0.0303068	0.00485249	3.69999e-10	180726
Sex hormone-binding globulin levels	Bioavailable testosterone levels	rs776746	T	C	7	99270539	0.067312	0.0152833	0.00245492	6.09958e-11	180726
Sex hormone-binding globulin levels	Bioavailable testosterone levels	rs7773423	G	A	6	116363149	0.40114	0.00749373	0.00125965	2.1e-09	180726
Sex hormone-binding globulin levels	Bioavailable testosterone levels	rs7860634	A	G	9	139089679	0.573824	0.0123675	0.00124597	6.09958e-25	180726
Sex hormone-binding globulin levels	Bioavailable testosterone levels	rs78679318	G	T	9	113033907	0.187187	0.00893484	0.0015856	2.39999e-09	180726
Sex hormone-binding globulin levels	Bioavailable testosterone levels	rs78890745	A	G	4	159834474	0.108878	0.0136529	0.00198065	8.9002e-12	180726
Sex hormone-binding globulin levels	Bioavailable testosterone levels	rs7920217	T	C	10	105668172	0.504413	0.00773882	0.00122079	1.09999e-09	180726
Sex hormone-binding globulin levels	Bioavailable testosterone levels	rs79287178	A	G	3	17229450	0.031751	-0.0397047	0.00368077	3.59998e-30	180726
Sex hormone-b	Bioavailable	rs7939	C	A	15	53739426	0.01382	-0.096	0.00536866	5.0003	1807

inding globulin levels	testosterone levels	1862						0251		5e-81	26
Sex hormone-binding globulin levels	Bioavailable testosterone levels	rs7970695	A	G	12	12146	0.62301	0.0194022	0.00127145	5.10035e-54	180726
Sex hormone-binding globulin levels	Bioavailable testosterone levels	rs79717793	A	G	10	5262267	0.155045	-0.0246105	0.00168938	2.60016e-55	180726
Sex hormone-binding globulin levels	Bioavailable testosterone levels	rs79760705	T	G	5	53298716	0.113023	-0.0175959	0.00194631	1.99986e-21	180726
Sex hormone-binding globulin levels	Bioavailable testosterone levels	rs8071650	A	G	17	73797871	0.306566	0.00918975	0.00129802	4.30031e-13	180726
Sex hormone-binding globulin levels	Bioavailable testosterone levels	rs816040	T	G	5	153421412	0.621449	-0.00773707	0.00126761	1.29999e-09	180726
Sex hormone-binding globulin levels	Bioavailable testosterone levels	rs820503	A	C	9	6667928	0.137437	-0.0112088	0.0017888	3.69999e-11	180726
Sex hormone-binding globulin levels	Bioavailable testosterone levels	rs838716	C	G	2	234294791	0.679494	-0.00731808	0.00131343	1.09999e-09	180726
Sex hormone-binding globulin levels	Bioavailable testosterone levels	rs849135	A	G	7	28196413	0.501403	0.00974471	0.00122939	2.09991e-17	180726
Sex	Bioavail	rs8	A	G	8	2287	0.5903	-0.	0.0012	2.9	18

hormone-binding globulin levels	able testosterone levels	76435				3533	37	00756735	6472	9985e-11	0726
Sex hormone-binding globulin levels	Bioavailable testosterone levels	rs9265285	C	T	6	31292157	0.835723	-0.0153547	0.00172826	5.50047e-19	180726
Sex hormone-binding globulin levels	Bioavailable testosterone levels	rs9288177	A	G	2	191585252	0.374299	-0.00898949	0.00127263	5.00035e-13	180726
Sex hormone-binding globulin levels	Bioavailable testosterone levels	rs9304665	A	T	19	47602577	0.76448	-0.0079175	0.00145512	1.29999e-08	180726
Sex hormone-binding globulin levels	Bioavailable testosterone levels	rs9379084	A	G	6	7231843	0.115346	-0.0153264	0.00198813	1e-15	180726
Sex hormone-binding globulin levels	Bioavailable testosterone levels	rs9461224	T	G	6	25936402	0.396951	0.010895	0.00126004	1.39991e-20	180726
Sex hormone-binding globulin levels	Bioavailable testosterone levels	rs9492	C	A	6	32610976	0.182992	-0.00947556	0.00176134	3.79997e-09	180726
Sex hormone-binding globulin levels	Bioavailable testosterone levels	rs9697210	A	G	9	131468740	0.145255	-0.0187441	0.00175123	1.10002e-29	180726
Sex hormone-binding globulin levels	Bioavailable testosterone levels	rs9739640	G	A	12	53783174	0.167995	-0.00948138	0.00166929	1.29999e-08	180726

Supplementary Table S8. Instruments for causal estimation from SHBG to estradiol level.

Exposure	Outcome	SNP	Effect allele	Other allele	Chromosome	Genetic position	Effect allele frequency	Beta	Standard error of beta	Pvalue	Sample size
Sex hormone-binding globulin levels	Estradiol levels	rs10027275	C	G	4	148981496	0.74126	-0.0125534	0.00141198	1.69981e-21	180726
Sex hormone-binding globulin levels	Estradiol levels	rs10041660	C	T	5	122650224	0.50476	0.00755274	0.00124541	8.19993e-10	180726
Sex hormone-binding globulin levels	Estradiol levels	rs10107182	T	C	8	59392737	0.663202	0.0124022	0.00130257	2.80027e-22	180726
Sex hormone-binding globulin levels	Estradiol levels	rs1037169	C	T	11	13361005	0.687205	-0.0115299	0.00133154	3.69999e-20	180726
Sex hormone-binding globulin levels	Estradiol levels	rs10733789	C	T	10	64948684	0.313039	0.0492531	0.00131963	1e-200	180726
Sex hormone-binding globulin levels	Estradiol levels	rs10737683	A	G	1	196894829	0.236779	-0.010124	0.00144072	4.30031e-12	180726
Sex hormone-binding globulin levels	Estradiol levels	rs10868080	A	T	9	86626769	0.744477	-0.0213279	0.00140815	1.50003e-57	180726
Sex hormone-binding	Estradiol	rs1087177	G	A	18	57851763	0.236131	-0.0098407	0.00145802	5.00035e-11	180726

globulin levels	levels	7						7			
Sex hormone-binding globulin levels	Estradiol levels	rs10881582	A	G	9	137256078	0.238037	0.0133501	0.00145058	9.30037e-21	180726
Sex hormone-binding globulin levels	Estradiol levels	rs10895277	G	A	11	102084940	0.340806	-0.00961547	0.00130251	8.9002e-15	180726
Sex hormone-binding globulin levels	Estradiol levels	rs11075253	A	C	16	15148646	0.296081	0.00922345	0.00135476	8.00018e-12	180726
Sex hormone-binding globulin levels	Estradiol levels	rs11112774	A	G	12	102838128	0.737279	-0.00731072	0.00138631	4.60002e-08	180726
Sex hormone-binding globulin levels	Estradiol levels	rs11589776	T	C	14	73230278	0.287721	0.00787242	0.00136551	2.19999e-08	180726
Sex hormone-binding globulin levels	Estradiol levels	rs11593347	T	C	14	25947436	0.680871	-0.010709	0.00132486	3.10027e-17	180726
Sex hormone-binding globulin levels	Estradiol levels	rs11752158	T	C	4	170994894	0.096496	0.0127725	0.00200799	2.80027e-11	180726
Sex hormone-binding globulin levels	Estradiol levels	rs11981233	G	T	19	50016479	0.078984	0.0261638	0.00227727	8.19974e-33	180726
Sex hormone-bi	Estradio	rs1120	T	C	13	11504746	0.231857	-0.0088	0.00146895	2.30001	180726

nding	l	359				4		12		e-10	
globulin	level	22									
levels	s										
Sex	Estr										
hormone-bi	adio	rs1									
nding	l	132						-0.0		2.90	
globulin	level	484				6610	0.0482	143	0.0029	001	180
levels	s	43	G	A	10	2542	94	674	4711	e-08	726
Sex	Estr										
hormone-bi	adio	rs1								3.49	
nding	l	141						-0.0		945	
globulin	level	653				2702	0.0231	871	0.0040	e-11	180
levels	s	49	C	G	1	1913	58	136	8954	1	726
Sex	Estr										
hormone-bi	adio	rs1									
nding	l	149				1504		0.0		4.19	
globulin	level	492				9824	0.1113	187	0.0019	952	180
levels	s	63	C	T	7	5	88	991	6067	e-25	726
Sex	Estr										
hormone-bi	adio	rs1									
nding	l	152				1848		-0.0		3.69	
globulin	level	766				6513	0.0166	285	0.0049	999	180
levels	s	19	A	T	1	2	26	399	3428	e-11	726
Sex	Estr										
hormone-bi	adio										
nding	l	rs1				1359		-0.0		4.20	
globulin	level	154				2519	0.7730	276	0.0014	049	180
levels	s	988	A	T	3	1	31	587	6575	e-88	726
Sex	Estr										
hormone-bi	adio	rs1						0.0			
nding	l	156						095		1.20	
globulin	level	472				2178	0.2387	530	0.0014	005	180
levels	s	2	T	C	11	330	4	7	6515	e-13	726
Sex	Estr										
hormone-bi	adio	rs1						0.0			
nding	l	161				1117		086			
globulin	level	025				3412	0.2166	783	0.0014	2.1e	180
levels	s	6	T	C	12	1	65	8	9523	-09	726
Sex	Estr										
hormone-bi	adio	rs1									
nding	l	162						-0.0		1.39	
globulin	level	179				2487	0.4536	127	0.0012	991	180
levels	s	2	T	C	14	1926	47	49	4244	e-25	726
Sex	Estr	rs1	T	C	13	1147	0.1707	0.0	0.0017	1.29	180

hormone-binding globulin levels	adiol levels	16338429				67040	52	0985678	2794	999e-09	726
Sex hormone-binding globulin levels	Estradiol levels	rs11666245	A	G	19	38229926	0.047306	-0.0163325	0.0029014	2.19999e-09	180726
Sex hormone-binding globulin levels	Estradiol levels	rs11734408	G	A	4	23882519	0.293256	0.00767697	0.00134935	3.29997e-10	180726
Sex hormone-binding globulin levels	Estradiol levels	rs11739158	T	C	5	72927292	0.427811	0.00829379	0.00124625	2.09991e-12	180726
Sex hormone-binding globulin levels	Estradiol levels	rs11743810	T	C	5	137802404	0.560069	0.00893646	0.00124066	1.99986e-12	180726
Sex hormone-binding globulin levels	Estradiol levels	rs117664177	T	C	12	101036562	0.01211	-0.051561	0.00573803	3.69999e-22	180726
Sex hormone-binding globulin levels	Estradiol levels	rs117734486	T	C	8	81061523	0.061081	-0.0133401	0.00261459	1.2e-08	180726
Sex hormone-binding globulin levels	Estradiol levels	rs117921873	G	A	8	81473835	0.066515	0.021022	0.00250814	4.70002e-18	180726
Sex hormone-binding globulin levels	Estradiol levels	rs118037027	T	A	12	21203424	0.040459	0.0222618	0.00313784	3.69999e-13	180726

Sex hormone-binding globulin levels	Estradiol levels	rs11856926	A	G	15	96223649	0.448829	-0.011587	0.00124664	1.9002e-22	180726
Sex hormone-binding globulin levels	Estradiol levels	rs11870307	G	A	17	7617787	0.780506	0.0526994	0.00145305	1e-200	180726
Sex hormone-binding globulin levels	Estradiol levels	rs12138136	A	T	1	149844305	0.086596	0.0152389	0.00220676	6.29941e-14	180726
Sex hormone-binding globulin levels	Estradiol levels	rs1229492	C	T	7	81564122	0.731518	-0.0114669	0.00139655	6.29941e-18	180726
Sex hormone-binding globulin levels	Estradiol levels	rs12605524	G	A	18	22073551	0.28752	-0.0076479 7	0.00137562	1.2e-08	180726
Sex hormone-binding globulin levels	Estradiol levels	rs12611046	A	G	19	14291704	0.150636	-0.00968	0.00177915	7.00003e-09	180726
Sex hormone-binding globulin levels	Estradiol levels	rs12694450	C	T	2	220019638	0.675226	-0.00706409	0.0013572	3.79997e-08	180726
Sex hormone-binding globulin levels	Estradiol levels	rs12696304	G	C	3	169481271	0.265645	0.00813109	0.00139236	1.7e-09	180726
Sex hormone-binding globulin	Estradiol level	rs12797706	A	G	11	65561369	0.234588	0.0120832	0.00146721	6.09958e-17	180726

levels	s										
Sex	Estr										
hormone-bi	adio	rs1									
nding	l	295						0.0		7.39	
globulin	level	056				1799	0.6139	110	0.0012	946	180
levels	s	2	T	C	17	5166	66	236	2815	e-21	726
Sex	Estr										
hormone-bi	adio	rs1						0.0			
nding	l	295						082		7.49	
globulin	level	308				5926	0.7950	792	0.0014	998	180
levels	s	7	T	C	17	5201	92	4	8126	e-09	726
Sex	Estr										
hormone-bi	adio	rs1						0.0			
nding	l	298				1485		079		9.59	
globulin	level	959				9123	0.2916	174	0.0013	997	180
levels	s	6	T	G	2	2	51	7	5721	e-10	726
Sex	Estr										
hormone-bi	adio	rs1									
nding	l	310						-0.0		1.20	
globulin	level	821				3443	0.6172	229	0.0012	005	180
levels	s	8	G	A	4	931	54	943	7338	e-79	726
Sex	Estr										
hormone-bi	adio	rs1									
nding	l	315						-0.0		7.59	
globulin	level	006				8820	0.4376	207	0.0012	976	180
levels	s	8	G	A	4	3828	37	329	3764	e-67	726
Sex	Estr										
hormone-bi	adio	rs1						0.0			
nding	l	337						082		2.19	
globulin	level	904				7425	0.2800	885	0.0013	999	180
levels	s	3	C	T	14	0126	09	2	9928	e-10	726
Sex	Estr										
hormone-bi	adio	rs1									
nding	l	386						-0.0		3.50	
globulin	level	115				5622	0.0768	148	0.0023	026	180
levels	s	41	C	T	5	8040	23	471	084	e-12	726
Sex	Estr										
hormone-bi	adio							-0.0			
nding	l	rs1						080			
globulin	level	388				5016	0.2152	367	0.0015	2e-0	180
levels	s	20	C	T	22	4078	84	6	0694	8	726
Sex	Estr	rs1				2197		0.0		2.69	
hormone-bi	adio	415				3000	0.3182	074	0.0013	998	180
nding	l	293	T	A	1	6	82	343	1195	e-09	726

globulin levels	levels							1			
Sex hormone-binding globulin levels	Estradiol levels	rs144647926	A	G	1	235467607	0.087356	0.0123175	0.00217179	1.40001e-09	180726
Sex hormone-binding globulin levels	Estradiol levels	rs149131600	T	C	19	35549122	0.17438	0.0205782	0.00162554	9.20026e-42	180726
Sex hormone-binding globulin levels	Estradiol levels	rs150844304	C	A	15	43726625	0.024905	-0.0745127	0.0039585	9.3994e-85	180726
Sex hormone-binding globulin levels	Estradiol levels	rs1538648	G	A	10	94820860	0.503197	-0.00757485	0.00122235	8.4004e-13	180726
Sex hormone-binding globulin levels	Estradiol levels	rs157935	G	T	7	130585553	0.303601	0.0121026	0.00134199	1.50003e-22	180726
Sex hormone-binding globulin levels	Estradiol levels	rs17036326	G	A	3	12389313	0.121798	0.0170022	0.00187533	1.20005e-18	180726
Sex hormone-binding globulin levels	Estradiol levels	rs17145750	T	C	7	73026378	0.161026	0.0107381	0.00167262	9.70063e-11	180726
Sex hormone-binding globulin levels	Estradiol levels	rs1730865	T	G	1	107605611	0.654768	-0.0260475	0.00129437	4.60045e-98	180726
Sex hormone-bi	Estradio	rs1736	A	G	19	2796021	0.287261	0.0173	0.00136123	2.29985	180726

nding globulin levels	l levels	180						849		e-41	
Sex hormone-binding globulin levels	Estradiol levels	rs174529	C	T	11	61543961	0.375889	-0.00774839	0.00127239	4.20001e-10	180726
Sex hormone-binding globulin levels	Estradiol levels	rs17580	A	T	14	94847262	0.048463	0.0304227	0.00286953	4.19952e-29	180726
Sex hormone-binding globulin levels	Estradiol levels	rs1776899	A	G	6	34193230	0.969325	-0.0226948	0.0035717	5.69994e-10	180726
Sex hormone-binding globulin levels	Estradiol levels	rs1782652	A	T	10	81074125	0.380625	-0.0127414	0.00127529	5.79963e-27	180726
Sex hormone-binding globulin levels	Estradiol levels	rs1801689	C	A	17	64210580	0.029871	-0.03343	0.00350564	1.80011e-21	180726
Sex hormone-binding globulin levels	Estradiol levels	rs1860018	A	G	17	13846723	0.443251	-0.00848468	0.00119697	2.49977e-13	180726
Sex hormone-binding globulin levels	Estradiol levels	rs187079266	A	G	17	7438801	0.013934	-0.072588	0.00515995	7.70016e-47	180726
Sex hormone-binding globulin levels	Estradiol levels	rs1871395	G	A	12	21352315	0.151789	-0.0325026	0.00171209	4.60045e-88	180726
Sex	Estr	rs1	A	G	7	1164	0.4530	-0.0	0.0012	9.60	180

hormone-binding globulin levels	adiol levels	87437				45091	37	0826966	3975	064e-14	726
Sex hormone-binding globulin levels	Estradiol levels	rs202200760	C	G	19	17346854	0.038592	0.0711034	0.00348775	3.69999e-98	180726
Sex hormone-binding globulin levels	Estradiol levels	rs2045345	G	A	1	26818894	0.194645	-0.0118503	0.00158288	1e-13	180726
Sex hormone-binding globulin levels	Estradiol levels	rs2073503	G	C	4	3536176	0.310531	0.00683324	0.00132685	2.1e-08	180726
Sex hormone-binding globulin levels	Estradiol levels	rs2123050	T	A	15	35224247	0.60582	-0.00886187	0.00126102	1.80011e-13	180726
Sex hormone-binding globulin levels	Estradiol levels	rs2138161	C	T	2	227095159	0.64815	-0.0130685	0.00128154	1.39991e-24	180726
Sex hormone-binding globulin levels	Estradiol levels	rs2239222	G	A	14	73011885	0.349619	0.0110595	0.00130116	5.50047e-17	180726
Sex hormone-binding globulin levels	Estradiol levels	rs2256657	G	A	10	65712613	0.853507	-0.0140088	0.00173169	6.29941e-18	180726
Sex hormone-binding globulin levels	Estradiol levels	rs2259305	A	G	10	93615903	0.476995	-0.0118863	0.00122291	2.90001e-22	180726

Sex hormone-binding globulin levels	Estradiol levels	rs2266782	A	G	1	171076966	0.416647	-0.00783493	0.00124201	4.09996e-10	180726
Sex hormone-binding globulin levels	Estradiol levels	rs2298058	T	C	13	95248566	0.306244	0.0111891	0.0013408	2.09991e-18	180726
Sex hormone-binding globulin levels	Estradiol levels	rs2301050	T	C	3	105401507	0.275057	-0.00678906	0.00137554	4.30002e-08	180726
Sex hormone-binding globulin levels	Estradiol levels	rs2330649	A	G	22	24295053	0.566063	-0.0100381	0.00126705	4.00037e-17	180726
Sex hormone-binding globulin levels	Estradiol levels	rs2351958	A	C	11	16248020	0.387523	-0.0101948	0.00126369	1.29987e-17	180726
Sex hormone-binding globulin levels	Estradiol levels	rs2575368	G	T	16	1817397	0.919707	-0.0120002	0.00227266	3.09999e-08	180726
Sex hormone-binding globulin levels	Estradiol levels	rs2612069	G	C	12	66215292	0.096031	-0.0120066	0.0020917	8.10009e-09	180726
Sex hormone-binding globulin levels	Estradiol levels	rs2642438	G	A	1	220970028	0.701828	0.0150028	0.00133645	3.40017e-31	180726
Sex hormone-binding globulin	Estradiol level	rs2694157	C	T	7	101907808	0.620654	0.00902024	0.00126663	7.89951e-14	180726

levels	s										
Sex	Estr										
hormone-bi	adio										
nding	l	rs2				1456		-0.0		5.30	
globulin	level	721				7701	0.5251	112	0.0012	029	180
levels	s	195	C	T	8	1	3	875	38	e-21	726
Sex	Estr										
hormone-bi	adio	rs2								2.39	
nding	l	839						-0.0		883	
globulin	level	486				4745	0.4603	295	0.0012	e-13	180
levels	s	4	A	G	17	0775	08	18	0382	8	726
Sex	Estr										
hormone-bi	adio	rs2									
nding	l	850						0.0		5.70	
globulin	level	749				7719	0.3757	139	0.0012	033	180
levels	s	1	A	G	4	7651	06	975	668	e-29	726
Sex	Estr										
hormone-bi	adio	rs2									
nding	l	854				1102		-0.0		4.90	
globulin	level	928				3013	0.7741	130	0.0015	004	180
levels	s	7	A	G	1	8	48	821	8912	e-17	726
Sex	Estr										
hormone-bi	adio	rs2						-0.0			
nding	l	860				1265		064			
globulin	level	176				0003	0.4181	411	0.0012	1.5e	180
levels	s	1	G	C	8	1	25	9	6207	-08	726
Sex	Estr										
hormone-bi	adio										
nding	l	rs2				1019		0.0		3.40	
globulin	level	862				1206	0.5003	108	0.0012	017	180
levels	s	954	C	T	10	4	43	541	1838	e-19	726
Sex	Estr										
hormone-bi	adio							0.0			
nding	l	rs2						092		4.79	
globulin	level	872				6234	0.2456	882	0.0014	954	180
levels	s	881	A	G	20	2801	62	2	3199	e-11	726
Sex	Estr										
hormone-bi	adio	rs2									
nding	l	877						0.0		1.89	
globulin	level	302				8807	0.1014	119	0.0020	998	180
levels	s	7	A	G	4	6227	46	206	5087	e-10	726
Sex	Estr	rs2						0.1			
hormone-bi	adio	892				9484	0.0198	383	0.0044	1e-2	180
nding	l	947	T	C	14	4947	23	03	1884	00	726

globulin levels	levels	4									
Sex hormone-binding globulin levels	Estradiol levels	rs2896906	C	T	16	4647417	0.193587	-0.00970772	0.00156221	1.09999e-10	180726
Sex hormone-binding globulin levels	Estradiol levels	rs2905801	T	C	17	29524974	0.705121	0.0140121	0.00130719	3.80014e-28	180726
Sex hormone-binding globulin levels	Estradiol levels	rs2914233	C	T	5	158013856	0.21093	-0.0085613	0.00151926	2.39999e-09	180726
Sex hormone-binding globulin levels	Estradiol levels	rs2924545	A	G	11	68883281	0.429092	-0.00664564	0.00124894	5e-09	180726
Sex hormone-binding globulin levels	Estradiol levels	rs2989330	G	A	21	40716209	0.340297	0.00743759	0.00131431	6.80002e-09	180726
Sex hormone-binding globulin levels	Estradiol levels	rs3006593	C	G	10	31171626	0.379929	0.00767249	0.00125767	7.90005e-10	180726
Sex hormone-binding globulin levels	Estradiol levels	rs3173833	G	T	7	150491084	0.573379	0.00768356	0.0012443	6.80002e-09	180726
Sex hormone-binding globulin levels	Estradiol levels	rs34255979	T	C	19	46384830	0.119463	0.0272468	0.00190546	4.00037e-51	180726
Sex hormone-bi	Estradio	rs3441	A	G	19	59023166	0.179875	0.0087	0.00160777	1.7e-08	180726

nding	l	506						676			
globulin	level	2						1			
levels	s										
Sex	Estr										
hormone-bi	adio	rs3									
nding	l	458						-0.0		1.10	
globulin	level	783				3230	0.1555	125	0.0017	002	180
levels	s	9	A	G	20	0671	51	669	0732	e-14	726
Sex	Estr										
hormone-bi	adio	rs3									
nding	l	485						-0.0		9.30	
globulin	level	858				1945	0.0786	157	0.0022	037	180
levels	s	8	G	C	19	7235	55	987	9031	e-15	726
Sex	Estr										
hormone-bi	adio	rs3						-0.0			
nding	l	486						080		3.59	
globulin	level	925				1009	0.4287	993	0.0012	998	180
levels	s	3	C	T	4	420	35	3	5064	e-12	726
Sex	Estr										
hormone-bi	adio	rs3						-0.0			
nding	l	513						073		7.29	
globulin	level	529				2036	0.5190	733	0.0012	962	180
levels	s	3	T	C	2	3666	66	2	2906	e-12	726
Sex	Estr										
hormone-bi	adio	rs3						-0.0			
nding	l	523						076		2.30	
globulin	level	433				3566	0.2574	075	0.0014	001	180
levels	s	7	T	C	9	1243	71	9	1397	e-08	726
Sex	Estr										
hormone-bi	adio	rs3									
nding	l	534						-0.0		8.00	
globulin	level	608				2578	0.4438	104	0.0012	018	180
levels	s	3	A	C	1	8425	54	562	3226	e-19	726
Sex	Estr										
hormone-bi	adio	rs3									
nding	l	608						0.0		4.49	
globulin	level	619				1651	0.5789	167	0.0012	987	180
levels	s	5	T	C	1	0894	71	429	3754	e-44	726
Sex	Estr										
hormone-bi	adio										
nding	l	rs3						-0.0			
globulin	level	730				6722	0.0403	188	0.0031	2.1e	180
levels	s	393	A	G	16	6405	98	678	6246	-10	726
Sex	Estr	rs3	T	G	1	4003	0.1971	-0.0	0.0015	1.20	180

hormone-binding globulin levels	adiol levels	768321				5928	78	155199	4112	005e-23	726
Sex hormone-binding globulin levels	Estradiol levels	rs3795128	C	T	20	39774163	0.483632	0.00977417	0.00123946	1.50003e-17	180726
Sex hormone-binding globulin levels	Estradiol levels	rs3890483	T	G	19	7220596	0.442264	-0.0111819	0.00124431	1.99986e-21	180726
Sex hormone-binding globulin levels	Estradiol levels	rs3891167	G	A	18	658423	0.257163	0.00815923	0.0014729	3.59998e-08	180726
Sex hormone-binding globulin levels	Estradiol levels	rs40270	C	A	5	55804552	0.772121	-0.0141665	0.00146708	1.29987e-22	180726
Sex hormone-binding globulin levels	Estradiol levels	rs4092465	G	A	18	55080437	0.647016	0.00857154	0.00130236	3.59998e-13	180726
Sex hormone-binding globulin levels	Estradiol levels	rs42945	G	A	16	58545426	0.51158	-0.00881296	0.00124194	3.19963e-13	180726
Sex hormone-binding globulin levels	Estradiol levels	rs4297769	A	G	17	7944048	0.389563	-0.0138222	0.0012432	1.9002e-28	180726
Sex hormone-binding globulin levels	Estradiol levels	rs4327534	A	C	4	157627879	0.319889	0.0070071	0.00131672	3.79997e-08	180726

Sex hormone-binding globulin levels	Estradiol levels	rs4671607	C	T	2	64905898	0.737605	-0.0156863	0.00140192	2.90001e-33	180726
Sex hormone-binding globulin levels	Estradiol levels	rs4675682	C	T	2	208402750	0.46019	-0.00894896	0.00123031	3.10027e-15	180726
Sex hormone-binding globulin levels	Estradiol levels	rs4715316	T	C	6	52628998	0.644472	0.0086613	0.00129252	1.99986e-13	180726
Sex hormone-binding globulin levels	Estradiol levels	rs4719366	A	G	7	1977906	0.397059	-0.00801322	0.0012634	2.19989e-11	180726
Sex hormone-binding globulin levels	Estradiol levels	rs4819867	A	C	22	20136379	0.297211	0.00688975	0.00135593	5e-09	180726
Sex hormone-binding globulin levels	Estradiol levels	rs4841133	G	A	8	9183664	0.908681	0.022133	0.00214218	1.39991e-28	180726
Sex hormone-binding globulin levels	Estradiol levels	rs4918722	T	C	10	113947040	0.727446	-0.00951318	0.00137242	5.10035e-14	180726
Sex hormone-binding globulin levels	Estradiol levels	rs540730	C	T	12	57807114	0.754418	-0.0177235	0.00142693	4.90004e-39	180726
Sex hormone-binding globulin	Estradiol level	rs5729432	G	C	16	88511548	0.265278	0.0104733	0.00140802	3.19963e-15	180726

levels	s										
Sex hormone-binding globulin levels	Estradiol levels	rs55869502	T	C	14	100825670	0.4951	-0.0113108	0.00123479	3.29989e-22	180726
Sex hormone-binding globulin levels	Estradiol levels	rs55987409	T	C	20	49569025	0.072538	0.0141619	0.00238657	1.29999e-09	180726
Sex hormone-binding globulin levels	Estradiol levels	rs56077345	C	G	7	44808650	0.071231	0.0136246	0.00240009	2.80001e-09	180726
Sex hormone-binding globulin levels	Estradiol levels	rs56332871	A	C	15	96714816	0.272043	0.0298253	0.00139111	4.19759e-111	180726
Sex hormone-binding globulin levels	Estradiol levels	rs57158761	G	A	3	185371172	0.43637	-0.00664562	0.00124159	8.70001e-09	180726
Sex hormone-binding globulin levels	Estradiol levels	rs58414555	C	A	20	37554152	0.305798	0.00751293	0.00134031	2.59998e-08	180726
Sex hormone-binding globulin levels	Estradiol levels	rs58912472	G	A	17	1626018	0.526709	0.00691033	0.00119919	2.5e-09	180726
Sex hormone-binding globulin levels	Estradiol levels	rs59203582	T	A	8	81424720	0.158108	0.017504	0.00169193	8.69961e-30	180726
Sex hormone-binding	Estradiol	rs6001814	G	A	19	3375572	0.121031	0.01315	0.00198109	1e-12	180726

globulin levels	levels	7									
Sex hormone-binding globulin levels	Estradiol levels	rs6005840	G	A	22	29101357	0.672701	-0.0168747	0.00131567	4.00037e-39	180726
Sex hormone-binding globulin levels	Estradiol levels	rs6088461	T	G	20	32905161	0.42567	-0.00784447	0.00125342	1.99986e-11	180726
Sex hormone-binding globulin levels	Estradiol levels	rs6130613	T	C	20	43054441	0.469585	0.017418	0.00125777	7.50067e-45	180726
Sex hormone-binding globulin levels	Estradiol levels	rs61686644	G	A	1	1508819	0.259771	0.0103828	0.00147133	5.30029e-14	180726
Sex hormone-binding globulin levels	Estradiol levels	rs62011286	A	G	15	63791125	0.342496	-0.0126136	0.00129832	1.10002e-25	180726
Sex hormone-binding globulin levels	Estradiol levels	rs62259939	A	G	3	49386047	0.438162	-0.00910972	0.00123926	2.09991e-13	180726
Sex hormone-binding globulin levels	Estradiol levels	rs624319	A	G	6	160772900	0.473485	0.0151961	0.00123751	5.70033e-40	180726
Sex hormone-binding globulin levels	Estradiol levels	rs62472728	T	C	7	143105566	0.060894	0.0170313	0.00260051	9.59997e-10	180726
Sex hormone-bi	Estradio	rs6261	T	C	11	32956492	0.045536	0.0187	0.00294867	5.19996	180726

nding globulin levels	l levels	8693						921		e-11	
Sex hormone-binding globulin levels	Estradiol levels	rs631695	G	T	11	69283303	0.582362	-0.0168256	0.00124634	8.69961e-47	180726
Sex hormone-binding globulin levels	Estradiol levels	rs6422513	A	G	1	93032467	0.792896	0.0129507	0.00150077	3.80014e-18	180726
Sex hormone-binding globulin levels	Estradiol levels	rs6480299	G	C	10	69631393	0.669646	-0.0109315	0.00130057	3.80014e-17	180726
Sex hormone-binding globulin levels	Estradiol levels	rs6532796	G	A	4	100042242	0.706404	-0.0200993	0.00134644	4.70002e-51	180726
Sex hormone-binding globulin levels	Estradiol levels	rs6541725	A	C	2	121338032	0.379609	-0.00773737	0.0012627	1.79999e-09	180726
Sex hormone-binding globulin levels	Estradiol levels	rs6717858	C	T	2	165539661	0.402974	0.0113005	0.00124935	6.20012e-21	180726
Sex hormone-binding globulin levels	Estradiol levels	rs6736913	G	A	2	42510018	0.978716	-0.0329121	0.00423623	2.99985e-15	180726
Sex hormone-binding globulin levels	Estradiol levels	rs6758199	T	C	2	70537173	0.066765	-0.016905	0.00246789	3.50026e-12	180726
Sex	Estr	rs6	G	A	3	2452	0.6940	0.0	0.0013	2.09	180

hormone-binding globulin levels	adiol levels	792725				0283	54	120526	744	991e-21	726
Sex hormone-binding globulin levels	Estradiol levels	rs6900473	G	A	6	130375810	0.688161	-0.00890429	0.00133223	7.39946e-12	180726
Sex hormone-binding globulin levels	Estradiol levels	rs6939861	A	G	6	41703041	0.261847	-0.0112806	0.00142004	1.10002e-15	180726
Sex hormone-binding globulin levels	Estradiol levels	rs6950023	G	T	7	97915635	0.8144	0.0316741	0.00157952	9.8992e-96	180726
Sex hormone-binding globulin levels	Estradiol levels	rs7085615	A	G	10	64768990	0.104062	0.0305713	0.00200364	9.70063e-59	180726
Sex hormone-binding globulin levels	Estradiol levels	rs7092853	T	C	10	64429307	0.850678	-0.0108014	0.00171373	6.79986e-11	180726
Sex hormone-binding globulin levels	Estradiol levels	rs7127472	T	C	11	61937658	0.214918	0.0089718	0.00150774	3.69999e-09	180726
Sex hormone-binding globulin levels	Estradiol levels	rs71480542	A	C	15	59966306	0.382197	0.00688656	0.00130727	3.29997e-08	180726
Sex hormone-binding globulin levels	Estradiol levels	rs7177179	C	T	15	40287982	0.348882	-0.00728003	0.00129819	3.89996e-08	180726

Sex	Estr										
hormone-bi	adio							-0.0			
nding	l	rs7						065		7.79	
globulin	level	201				1113	0.4044	927	0.0012	992	180
levels	s	30	T	G	16	2633	37	8	5987	e-09	726
Sex	Estr										
hormone-bi	adio										
nding	l	rs7						-0.0		5.19	
globulin	level	225				7324	0.8325	133	0.0016	996	180
levels	s	349	A	G	17	0009	22	437	0389	e-17	726
Sex	Estr										
hormone-bi	adio	rs7						0.0			
nding	l	266				1041		092		2.59	
globulin	level	681				2914	0.2044	226	0.0015	998	180
levels	s	7	A	G	4	1	51	8	249	e-10	726
Sex	Estr										
hormone-bi	adio	rs7									
nding	l	268						0.0		2.99	
globulin	level	392				5073	0.0196	339	0.0044	985	180
levels	s	3	C	T	14	5947	79	786	3863	e-16	726
Sex	Estr										
hormone-bi	adio	rs7									
nding	l	278						0.0		7.10	
globulin	level	758				3301	0.0233	246	0.0040	003	180
levels	s	1	A	G	2	2808	97	186	9905	e-10	726
Sex	Estr										
hormone-bi	adio	rs7									
nding	l	284						-0.0		3.40	
globulin	level	281				7296	0.0333	653	0.0035	017	180
levels	s	1	T	C	17	899	89	578	7483	e-77	726
Sex	Estr										
hormone-bi	adio	rs7									
nding	l	292						0.0		6.20	
globulin	level	984				6168	0.0765	196	0.0023	012	180
levels	s	7	A	G	1	4630	46	337	0744	e-18	726
Sex	Estr										
hormone-bi	adio	rs7									
nding	l	294				1781		-0.0		4.70	
globulin	level	811				6708	0.0948	122	0.0020	002	180
levels	s	5	T	C	2	6	7	848	9101	e-09	726
Sex	Estr										
hormone-bi	adio	rs7				1115		0.0		1.50	
nding	l	314				2202	0.0684	301	0.0024	003	180
globulin	level	285	G	T	12	6	74	165	3544	e-41	726

levels	s										
Sex hormone-binding globulin levels	Estradiol levels	rs731839	A	G	19	33899065	0.665034	0.00689626	0.0013066	2.19999e-08	180726
Sex hormone-binding globulin levels	Estradiol levels	rs738409	G	C	22	44324727	0.216244	0.0309602	0.00149718	1.80011e-96	180726
Sex hormone-binding globulin levels	Estradiol levels	rs7430950	C	A	3	196233136	0.252159	-0.00851251	0.00141712	4.20001e-10	180726
Sex hormone-binding globulin levels	Estradiol levels	rs74551598	C	A	9	117177566	0.243984	-0.00836883	0.00143759	1e-09	180726
Sex hormone-binding globulin levels	Estradiol levels	rs75130744	C	G	12	25410741	0.071554	-0.0296028	0.00238856	3.10027e-39	180726
Sex hormone-binding globulin levels	Estradiol levels	rs7540115	C	A	1	200265618	0.818722	0.00936579	0.0015874	1.09999e-09	180726
Sex hormone-binding globulin levels	Estradiol levels	rs75631642	C	T	4	18049216	0.216919	-0.00849796	0.00152246	2.19999e-08	180726
Sex hormone-binding globulin levels	Estradiol levels	rs76895963	G	T	12	4384844	0.020899	0.0554107	0.00473659	2.39994e-33	180726
Sex hormone-binding	Estradiol	rs7748356	T	C	4	111096883	0.016978	0.0303068	0.00485249	3.69999e-10	180726

globulin levels	levels	4									
Sex hormone-binding globulin levels	Estradiol levels	rs776746	T	C	7	99270539	0.067312	0.0152833	0.00245492	6.09958e-11	180726
Sex hormone-binding globulin levels	Estradiol levels	rs7773423	G	A	6	116363149	0.40114	0.00749373	0.00125965	2.1e-09	180726
Sex hormone-binding globulin levels	Estradiol levels	rs7860634	A	G	9	139089679	0.573824	0.0123675	0.00124597	6.09958e-25	180726
Sex hormone-binding globulin levels	Estradiol levels	rs78679318	G	T	9	113033907	0.187187	0.00893484	0.0015856	2.39999e-09	180726
Sex hormone-binding globulin levels	Estradiol levels	rs78890745	A	G	4	159834474	0.108878	0.0136529	0.00198065	8.9002e-12	180726
Sex hormone-binding globulin levels	Estradiol levels	rs7920217	T	C	10	105668172	0.504413	0.00773882	0.00122079	1.09999e-09	180726
Sex hormone-binding globulin levels	Estradiol levels	rs79287178	A	G	3	172294500	0.031751	-0.0397047	0.00368077	3.59998e-30	180726
Sex hormone-binding globulin levels	Estradiol levels	rs79391862	C	A	15	53739426	0.01382	-0.0960251	0.00536866	5.00035e-81	180726
Sex hormone-bi	Estradio	rs7970	A	G	12	12142337	0.62301	0.0194	0.00127145	5.10035	180726

nding globulin levels	l levels	695				6		022		e-54	
Sex hormone-binding globulin levels	Estradiol levels	rs79717793	A	G	10	5262267	0.155045	-0.0246105	0.00168938	2.60016e-55	180726
Sex hormone-binding globulin levels	Estradiol levels	rs79760705	T	G	5	53298716	0.113023	-0.0175959	0.00194631	1.99986e-21	180726
Sex hormone-binding globulin levels	Estradiol levels	rs8071650	A	G	17	73797871	0.306566	0.00918975	0.00129802	4.30031e-13	180726
Sex hormone-binding globulin levels	Estradiol levels	rs8113367	G	A	19	18388250	0.633885	0.00756423	0.00128012	2.30001e-09	180726
Sex hormone-binding globulin levels	Estradiol levels	rs816040	T	G	5	153421412	0.621449	-0.00773707	0.00126761	1.29999e-09	180726
Sex hormone-binding globulin levels	Estradiol levels	rs820503	A	C	9	6667928	0.137437	-0.0112088	0.00178888	3.69999e-11	180726
Sex hormone-binding globulin levels	Estradiol levels	rs838716	C	G	2	234294791	0.679494	-0.00731808	0.00131343	1.09999e-09	180726
Sex hormone-binding globulin levels	Estradiol levels	rs849135	A	G	7	28196413	0.501403	0.00974471	0.00122939	2.09991e-17	180726
Sex	Estr	rs8	G	A	17	7499	0.5830	-0.0	0.0012	1e-2	180

hormone-binding globulin levels	adiol levels	58526				382	99	452906	1789	00	726
Sex hormone-binding globulin levels	Estradiol levels	rs876435	A	G	8	22873533	0.590337	-0.00756735	0.00126472	2.99985e-11	180726
Sex hormone-binding globulin levels	Estradiol levels	rs9265285	C	T	6	31292157	0.835723	-0.0153547	0.00172826	5.50047e-19	180726
Sex hormone-binding globulin levels	Estradiol levels	rs9288177	A	G	2	191585252	0.374299	-0.00898949	0.00127263	5.00035e-13	180726
Sex hormone-binding globulin levels	Estradiol levels	rs9304665	A	T	19	47602577	0.76448	-0.0079175	0.00145512	1.29999e-08	180726
Sex hormone-binding globulin levels	Estradiol levels	rs9379084	A	G	6	7231843	0.115346	-0.0153264	0.00198813	1e-15	180726
Sex hormone-binding globulin levels	Estradiol levels	rs9461224	T	G	6	25936402	0.396951	0.010895	0.00126004	1.39991e-20	180726
Sex hormone-binding globulin levels	Estradiol levels	rs9492	C	A	6	32610976	0.182992	-0.00947556	0.00176134	3.79997e-09	180726
Sex hormone-binding globulin levels	Estradiol levels	rs9697210	A	G	9	131468740	0.145255	-0.0187441	0.00175123	1.10002e-29	180726

Sex hormone-binding globulin levels	Estradiol levels	rs9739640	G	A	12	53783174	0.167995	-0.00948138	0.00166929	1.29999e-08	180726

Supplementary Table S9. Instruments for causal estimation from BioT level to SHBG.

Exposure	Outcome	SNP	Effect allele	Other allele	Chromosome	Genetic position	Effect allele frequency	Beta	Standard error of beta	Pvalue	Sample size
Bioavailable testosterone levels	Sex hormone-binding globulin levels	rs10279715	G	A	7	40870935	0.462118	-0.0217388	0.00306173	3.10027e-13	178782
Bioavailable testosterone levels	Sex hormone-binding globulin levels	rs10738700	G	A	9	24973797	0.430636	-0.0204816	0.00300877	5.60015e-11	178782
Bioavailable testosterone levels	Sex hormone-binding globulin levels	rs10867084	C	T	9	140774721	0.489079	0.0226393	0.00304395	6.79986e-14	178782
Bioavailable testosterone levels	Sex hormone-binding globulin levels	rs10982156	A	T	9	117088064	0.069545	0.0477036	0.00622867	4.49987e-14	178782
Bioavailable testosterone levels	Sex hormone-binding globulin levels	rs1112195	G	A	3	24085166	0.495419	0.0182475	0.00306001	4.70002e-10	178782
Bioavailable testosterone levels	Sex hormone-binding globulin levels	rs112217694	C	G	4	104641064	0.05773	-0.0494985	0.00656267	1.20005e-14	178782
Bioavailable testosterone levels	Sex hormone-binding globulin levels	rs114492985	C	T	4	70572301	0.11256	-0.0249099	0.00483022	3.69999e-08	178782
Bioavailable	Sex hormone-b	rs1145	A	T	4	10470557	0.03578	-0.05	0.00829446	8.6000	178782

testoster	inding	488				8		28		3e-	
one	globulin	05						06		10	
levels	levels							4			
Bioavail	Sex										
able	hormone-b	rs1						-0.		2.8	
testoster	inding	152				1047		22		002	
one	globulin	602				7469	0.0119	71	0.0146	7e-	178
levels	levels	27	G	A	4	8	39	47	193	56	782
Bioavail	Sex										
able	hormone-b							0.0		1.3	
testoster	inding	rs1						26		999	
one	globulin	272				6088	0.3866	76	0.0031	1e-	178
levels	levels	131	C	T	14	6150	95	42	5783	17	782
Bioavail	Sex							-0.			
able	hormone-b	rs1						03		2.7	
testoster	inding	302						08		002	
one	globulin	847				1171	0.5535	99	0.0030	3e-	178
levels	levels	9	T	G	2	2075	1	4	7167	24	782
Bioavail	Sex							-0.			
able	hormone-b	rs1						03		8.8	
testoster	inding	306						20		003	
one	globulin	546				6166	0.1308	51	0.0045	5e-	178
levels	levels	3	A	G	3	2996	52	9	199	13	782
Bioavail	Sex										
able	hormone-b							0.0		4.9	
testoster	inding	rs1						18		000	
one	globulin	383				8905	0.4242	98	0.0030	4e-	178
levels	levels	5	A	C	15	6040	08	02	8562	10	782
Bioavail	Sex										
able	hormone-b	rs1						-0.		8.6	
testoster	inding	453						07		996	
one	globulin	599				7850	0.0241	76	0.0106	1e-	178
levels	levels	38	C	G	7	4431	11	46	751	15	782
Bioavail	Sex							-0.			
able	hormone-b	rs1						08			
testoster	inding	461						78			
one	globulin	154				2937	0.0185	74	0.0113	1e-	178
levels	levels	16	T	C	11	8978	66	2	221	15	782
Bioavail	Sex										
able	hormone-b	rs1						0.0		7.5	
testoster	inding	770						44		006	
one	globulin	388				5153	0.2552	27	0.0035	7e-	178
levels	levels	3	C	T	15	0097	65	84	0043	40	782
Bioavail	Sex	rs1	C	T	14	9000	0.1999	-0.	0.0038	5.1	178

able testosterone levels	hormone-binding globulin levels	812755				7637	44	033863	5917	0035e-19	782
Bioavailable testosterone levels	Sex hormone-binding globulin levels	rs2011425	G	T	2	234627608	0.079615	-0.0502524	0.00565644	6.4003e-20	178782
Bioavailable testosterone levels	Sex hormone-binding globulin levels	rs2090409	A	C	9	108967088	0.315943	-0.0309321	0.0032844	1.69981e-21	178782
Bioavailable testosterone levels	Sex hormone-binding globulin levels	rs2327121	C	G	20	8878250	0.65709	0.0181409	0.00322022	1.29999e-08	178782
Bioavailable testosterone levels	Sex hormone-binding globulin levels	rs2438086	G	A	2	105871129	0.57583	0.0218279	0.00307572	6.89922e-13	178782
Bioavailable testosterone levels	Sex hormone-binding globulin levels	rs2631864	G	A	8	21112084	0.103625	0.0331054	0.00501603	8.30042e-12	178782
Bioavailable testosterone levels	Sex hormone-binding globulin levels	rs2668776	T	C	18	44750365	0.530719	-0.0292238	0.00305978	1.69981e-22	178782
Bioavailable testosterone levels	Sex hormone-binding globulin levels	rs2764772	A	T	16	20060653	0.33405	0.0370436	0.003233	5.40008e-33	178782
Bioavailable testosterone levels	Sex hormone-binding globulin levels	rs2959132	T	C	11	28844928	0.556869	-0.0188414	0.00306042	8.19974e-11	178782

Bioavailable testosterone levels	Sex hormone-binding globulin levels	rs2961853	C	T	5	165932048	0.468671	0.0191152	0.00305362	3.2e-10	178782
Bioavailable testosterone levels	Sex hormone-binding globulin levels	rs3020421	A	G	6	152348122	0.327194	-0.0185099	0.0032529	1e-08	178782
Bioavailable testosterone levels	Sex hormone-binding globulin levels	rs34040779	C	T	3	107235109	0.075671	-0.0348252	0.00591069	2.19999e-09	178782
Bioavailable testosterone levels	Sex hormone-binding globulin levels	rs34192788	A	T	6	17416258	0.309674	-0.0198155	0.00332811	4.20001e-09	178782
Bioavailable testosterone levels	Sex hormone-binding globulin levels	rs3742223	C	T	13	112725196	0.096604	-0.0433217	0.00521873	1e-16	178782
Bioavailable testosterone levels	Sex hormone-binding globulin levels	rs4562360	G	A	8	61704817	0.756947	0.0323526	0.00355024	3.59998e-20	178782
Bioavailable testosterone levels	Sex hormone-binding globulin levels	rs4872310	A	G	8	25247181	0.247256	-0.0231059	0.00352473	3.69999e-12	178782
Bioavailable testosterone levels	Sex hormone-binding globulin levels	rs503542	G	A	11	118590743	0.450311	0.0183001	0.00300761	5.80003e-10	178782
Bioavailable testosterone	Sex hormone-binding globulin	rs55795858	C	T	7	146123500	0.329414	0.0179944	0.003298	1.5e-08	178782

levels	levels										
Bioavailable testosterone levels	Sex hormone-binding globulin levels	rs57606486	A	G	14	35796645	0.026698	0.0532939	0.00951357	2.1e-08	178782
Bioavailable testosterone levels	Sex hormone-binding globulin levels	rs62041532	G	T	16	73922719	0.212619	0.0233361	0.00372844	1.6e-10	178782
Bioavailable testosterone levels	Sex hormone-binding globulin levels	rs6486542	T	C	12	131252209	0.429429	-0.0261149	0.00309397	2.90001e-18	178782
Bioavailable testosterone levels	Sex hormone-binding globulin levels	rs6718154	T	C	2	180497923	0.274755	0.0322455	0.00340933	1.20005e-22	178782
Bioavailable testosterone levels	Sex hormone-binding globulin levels	rs7089031	G	T	10	101693155	0.393477	-0.0174188	0.0031234	3.2e-08	178782
Bioavailable testosterone levels	Sex hormone-binding globulin levels	rs714786	A	G	3	53789022	0.373237	-0.0213182	0.00314574	3.69999e-13	178782
Bioavailable testosterone levels	Sex hormone-binding globulin levels	rs71529289	T	C	8	77879487	0.251898	-0.0359428	0.00347805	1.99986e-25	178782
Bioavailable testosterone levels	Sex hormone-binding globulin levels	rs7265992	A	G	20	33525407	0.178841	-0.0317217	0.00402147	4.00037e-16	178782
Bioavailable testoster	Sex hormone-binding	rs7266493	C	T	1	32274901	0.575433	0.01867	0.00309656	1.7e-09	178782

one levels	globulin levels	5						69			
Bioavailable testosterone levels	Sex hormone-binding globulin levels	rs745486	T	C	9	11242155	0.281397	-0.0213054	0.00338763	1.2e-10	178782
Bioavailable testosterone levels	Sex hormone-binding globulin levels	rs76592891	C	T	6	27572715	0.048315	0.0429054	0.00718355	9.80009e-10	178782
Bioavailable testosterone levels	Sex hormone-binding globulin levels	rs7679843	G	C	4	22028079	0.094982	0.0501614	0.00533198	3.80014e-22	178782
Bioavailable testosterone levels	Sex hormone-binding globulin levels	rs7700874	C	T	5	95808483	0.401356	0.0193016	0.00310924	4e-10	178782
Bioavailable testosterone levels	Sex hormone-binding globulin levels	rs7758796	A	G	6	100105028	0.451796	0.0290903	0.00307465	5.10035e-22	178782
Bioavailable testosterone levels	Sex hormone-binding globulin levels	rs7857865	A	G	9	1957974	0.475348	0.0173108	0.00306171	5.80003e-09	178782
Bioavailable testosterone levels	Sex hormone-binding globulin levels	rs7912521	C	T	10	67262089	0.415588	0.0611199	0.00308282	6.20012e-94	178782
Bioavailable testosterone levels	Sex hormone-binding globulin levels	rs7915430	G	T	10	121660465	0.202149	-0.020841	0.00379473	9.69996e-09	178782
Bioavailable	Sex hormone-b	rs7928	T	C	11	12509522	0.721798	-0.02	0.00339392	1.1000	178782

testoster	inding	369				1		40		2e-	
one	globulin							24		13	
levels	levels							8			
Bioavail	Sex							-0.			
able	hormone-b							02			
testoster	inding	rs8						38			
one	globulin	076				7561	0.7041	70	0.0033	1e-	178
levels	levels	703	T	C	17	2643	35	9	3901	12	782
Bioavail	Sex										
able	hormone-b							0.0		2.4	
testoster	inding	rs9						40		997	
one	globulin	122				7722	0.3433	19	0.0032	7e-	178
levels	levels	02	C	G	9	5603	28	86	0718	38	782
Bioavail	Sex							-0.			
able	hormone-b							04		2.0	
testoster	inding	rs9				1053		87		999	
one	globulin	322				6959		09	0.0032	1e-	178
levels	levels	822	T	C	6	8	0.3215	2	5272	52	782
Bioavail	Sex							-0.			
able	hormone-b							03		3.1	
testoster	inding	rs9				1356		36		002	
one	globulin	507				8054	0.1373	83	0.0044	7e-	178
levels	levels	16	G	A	5	0	81	3	2472	14	782
Bioavail	Sex										
able	hormone-b							0.0		4.1	
testoster	inding	rs9						25		001	
one	globulin	824				2880	0.7208	98	0.0033	5e-	178
levels	levels	196	T	G	3	7441	96	6	9312	16	782
Bioavail	Sex										
able	hormone-b							0.0		2.8	
testoster	inding	rs9						55		002	
one	globulin	986				1501	0.5068	60	0.0030	7e-	178
levels	levels	829	A	G	7	9259	65	73	5395	76	782

Supplementary Table S10. Instruments for causal estimation from estradiol level to SHBG.

Exposure	Outcome	SNP	Effect allele	Other allele	Chromosome	Genetic position	Effect allele frequency	Beta	Standard error of beta	Pvalue	Sample size
Estradiol levels	Sex hormone-binding globulin levels	rs10006452	C	T	4	69977808	0.48021	-0.00728098	0.000844823	5.30029e-18	206927
Estradiol levels	Sex hormone-binding globulin levels	rs10491431	A	C	5	35968000	0.141	0.00774443	0.00121717	1.79999e-10	206927
Estradiol levels	Sex hormone-binding globulin levels	rs12881196	G	C	2	31982811	0.039212	0.0249518	0.00219839	5.60015e-30	206927
Estradiol levels	Sex hormone-binding globulin levels	rs13047993	T	C	18	20585399	0.068984	-0.00966159	0.00168082	8.70001e-09	206927
Estradiol levels	Sex hormone-binding globulin levels	rs17826558	T	C	22	46770756	0.033971	0.0136375	0.00243043	2.69998e-08	206927
Estradiol levels	Sex hormone-binding globulin levels	rs2547234	C	T	19	48383906	0.834781	-0.00760122	0.00113891	2.70023e-11	206927
Estradiol levels	Sex hormone-binding globulin levels	rs34019140	A	G	14	106527500	0.435748	-0.011661	0.000862566	6.89922e-42	206927
Estradiol level	Sex hormone-binding	rs3751591	G	A	15	51606710	0.166931	0.00777766	0.00113687	6.59933e-	206927

s	globulin levels									12	
Estradiol levels	Sex hormone-binding globulin levels	rs45446698	G	T	7	99332948	0.042435	-0.0156669	0.00209754	7.89951e-14	206927
Estradiol levels	Sex hormone-binding globulin levels	rs56196860	A	C	12	2908330	0.031492	0.0209011	0.0024192	2.60016e-18	206927
Estradiol levels	Sex hormone-binding globulin levels	rs657152	A	C	9	136139265	0.338866	-0.00835302	0.000899229	5.50047e-21	206927
Estradiol levels	Sex hormone-binding globulin levels	rs7173595	T	C	15	51533736	0.64871	0.0158526	0.00088621	3.59998e-72	206927
Estradiol levels	Sex hormone-binding globulin levels	rs727428	C	T	17	7537792	0.557221	0.00566325	0.000852078	1.80011e-11	206927
